# *Torpedo californica* acetylcholinesterase is stabilized by binding of a divalent metal ion to a novel and versatile 4D motif

**DOI:** 10.1002/pro.4061

**Published:** 2021-03-29

**Authors:** Israel Silman, Valery L. Shnyrov, Yacov Ashani, Esther Roth, Anne Nicolas, Joel L. Sussman, Lev Weiner

**Affiliations:** ^1^ Department of Neurobiology Weizmann Institute of Science Rehovot Israel; ^2^ Department of Biochemistry and Molecular Biology Universidad de Salamanca Salamanca Spain; ^3^ Department of Biomolecular Sciences Weizmann Institute of Science Rehovot Israel; ^4^ Department of Chemical and Structural Biology Weizmann Institute of Science Rehovot Israel; ^5^ Structural Proteomics Unit Weizmann Institute of Science Rehovot Israel; ^6^ Department of Chemical Research Support Weizmann Institute of Science Rehovot Israel

**Keywords:** crystal structures, differential scanning calorimetry, divalent metal ion, electron paramagnetic resonance, thermal inactivation

## Abstract

Stabilization of *Torpedo californica* acetylcholinesterase by the divalent cations Ca^+2^, Mg^+2^, and Mn^+2^ was investigated. All three substantially protect the enzyme from thermal inactivation. Electron paramagnetic resonance revealed one high‐affinity binding site for Mn^+2^ and several much weaker sites. Differential scanning calorimetry showed a single irreversible thermal transition. All three cations raise both the temperature of the transition and the activation energy, with the transition becoming more cooperative. The crystal structures of the Ca^+2^ and Mg^+2^ complexes with *Torpedo* acetylcholinesterase were solved. A principal binding site was identified. In both cases, it consists of four aspartates (a 4D motif), within which the divalent ion is embedded, together with several water molecules. It makes direct contact with two of the aspartates, and indirect contact, via waters, with the other two. The 4D motif has been identified in 31 acetylcholinesterase sequences and 28 butyrylcholinesterase sequences. Zebrafish acetylcholinesterase also contains the 4D motif; it, too, is stabilized by divalent metal ions. The ASSAM server retrieved 200 other proteins that display the 4D motif, in many of which it is occupied by a divalent cation. It is a very versatile motif, since, even though tightly conserved in terms of RMSD values, it can contain from one to as many as three divalent metal ions, together with a variable number of waters. This novel motif, which binds primarily divalent metal ions, is shared by a broad repertoire of proteins. An animated Interactive 3D Complement (I3DC) is available in Proteopedia at http://proteopedia.org/w/Journal:Protein_Science:3.

## Abstract 2

PDB‐ID(s): 7B38, 7B8E and 7B2W;

**Abbreviations**
ACh: acetylcholine
ATC: acetylthiocholine
AChE: acetylcholinesterase
BChE: butyrylcholinesterase
CAS: catalytic anionic site
DSC: differential scanning calorimetry
*Ee*: *Electrophorus electricus*
EPR: electron paramagnetic resonance
GPI: glycophosphatidylinositol
Hu: human
kDa: kilodalton
MES: 2‐morpholinoethanesulfonic acid
Mo: mouse
PAS: peripheral anionic site
PEG: polyethylene glycol
PDB: Protein Data Bank
PI: phosphatidylinositol
*Tc*: *Torpedo californica*
*Tm*: *Torpedo marmorata*

## 1 INTRODUCTION

The presence of metal ions in proteins is usually considered in the context of their involvement in the catalytic action of enzymes, whether as individual ions complexed with amino acid side chains, or as parts of prosthetic groups, as in the case of the heme proteins.^1^ However, metal ions may also assist in the folding and stabilization of protein structures.^2^, ^3^, ^4^, ^5^, ^6^ Furthermore, since the charged amino acids with which they interact are unlikely to be deeply buried, they can even work as “molecular staples,” on the exterior of proteins, to assist in the binding of ligands.^7^ Well known metal‐binding motifs include the EF‐hand, which binds Ca^+2^ ions,^8^ and the zinc finger.^9^

Acetylcholinesterase (AChE) is a powerful enzyme, which hydrolyzes the neurotransmitter acetylcholine (ACh) at a rate that approaches the limit of diffusion control.^10^, ^11^ Its catalytic subunit contains over 530 amino acid residues, and there is evidence for involvement in its catalytic activity of residues as far apart as Tyr70, at the peripheral anionic site (PAS), and Tyr442, at the backdoor (*Torpedo californica* [*Tc*] AChE numbering).^12^, ^13^ Upon thermal denaturation *Tc*AChE unfolds irreversibly from its native state (N) to a molten globule (MG) in a two‐state transition.^14^, ^15^ Thermal inactivation and denaturation is retarded in the presence of the divalent cations, Ca^+2^, Mg^+2^, and Mn^+2^, and it was indeed suggested that the effects observed involve a specific binding site for the divalent ions.^16^ The presence of an EF‐hand has been reported in *Tc*AChE.^17^

In one of the heavy atom derivatives of *Tc*AChE used to solve its crystal structure, a UO_2_^+2^ ion is located in a pocket containing four aspartate residues at a locus remote from the active site.^18^ In the following, we present biochemical, biophysical, and structural evidence that this heavy‐atom site serves as a specific binding site for the divalent cations that were earlier shown to stabilize the enzyme,^16^ and present bioinformatic evidence that this 4D motif is a conserved motif that serves as a divalent metal ion binding site in diverse proteins.

## 2 RESULTS AND DISCUSSION

### 2.1 *Kinetic measurements*

As seen in Table 1, Mg^+2^, at concentrations up to 10 mM, only slightly modifies the *k*_cat_ and *K*_*m*_ values for *Tc*AChE acting on acetylthiocholine (ATC). This results in a correspondingly small effect on *k*_cat_/*K*_*m*_, which increases by less than 50% on going from 0 to 10 mM MgCl_2_. The Standard Errors of the Mean (SEM) for K_m_ and *k*_*cat*_ are less than 10% of the shown values in Table.1. They were obtained from the regression line fitted to Michaelis‐Menten plots using GraphPad Prism software (ver. 8). The K_m_ values are in the range of those previously reported for the *Torpedo* enzyme.^19^, ^20^

**TABLE 1:** Kinetic constants for action of *Tc*AChE on ATC in the presence and absence of MgCl_2_

MgCl_2_, mM	K_m_, mM	*k*_*cat*_ (×10^5^), min^−1^	*k*_*cat*_/K_m_ (×10^9^), M^−1^ min^−1^
0	0.093	3.20	3.44
0	0.081	3.00	3.70
0	0.088	2.90	3.29
0.02	0.076	3.64	4.79
0.20	0.087	4.10	4.71
2.00	0.079	4.01	5.07
5.00	0.090	4.10	4.55
10.00	0.087	4.25	4.89

### 2.2 *Thermal inactivation*

**FIGURE 1:**
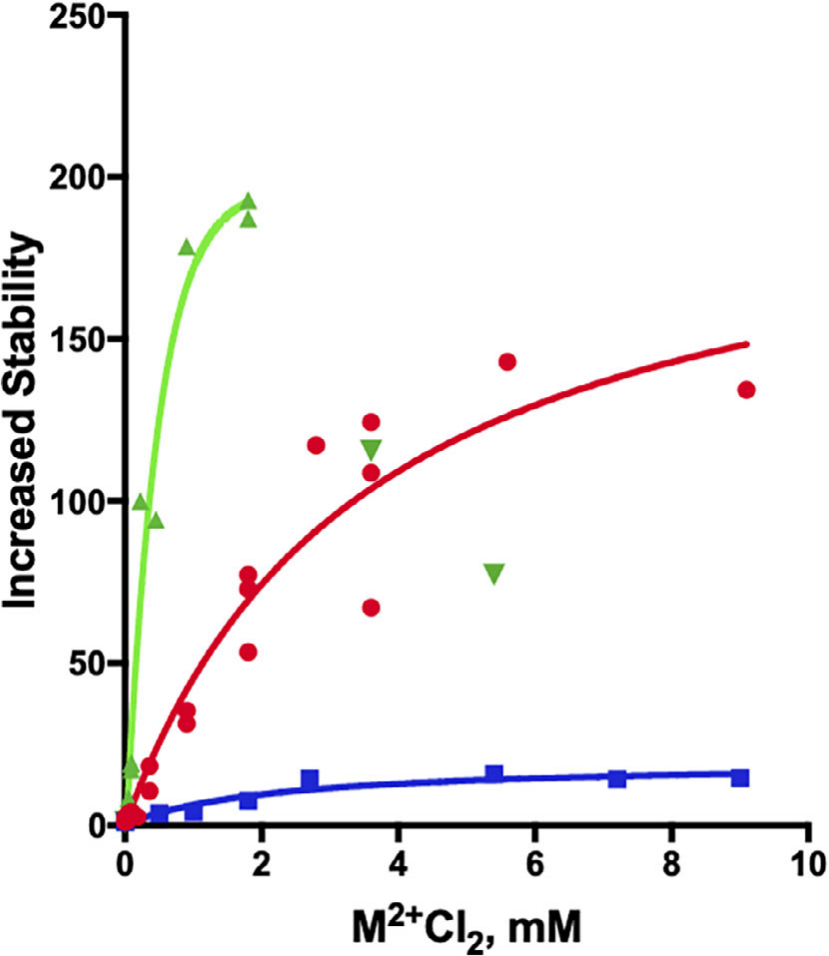
Thermal inactivation of *Tc*AChE in the absence and presence of divalent metal ions. The curves show the fold increase in stability at 39°C, in terms of the half‐life for loss of activity, as a function of the concentrations of the divalent ions, relative to the value of 1 in their absence. 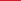, MgCl_2_; 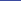, CaCl_2_; 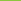, MnCl_2_

It was earlier shown that *Tc*AChE is quite sensitive to thermal inactivation, losing ~50% of its activity within ~5 min at 41°C.^15^ It was subsequently demonstrated that the divalent cations, Ca^+2^, Mg^+2^, and Mn^+2^, stabilize the enzyme considerably at millimolar concentrations.^16^ Figure 1 shows that, at concentrations approaching 10 mM, Mg^+2^ stabilizes *Tc*AChE by 150‐fold at 39°C, achieving fourfold stabilization already at 0.1 mM. The effect of Ca^+2^ is much less dramatic, stabilization plateauing at 10‐fold at ~4 mM. Mn^+2^ stabilizes even more effectively than Mg^+2^, approaching 200‐fold stabilization at 1.5 mM. However, at 3 mM Mn^+2^ and above, activity decreases (see unconnected data points). Two plausible reasons for this deactivation can be offered. One is that the Mn^+2^ ion attacks the buried Cys231, as has been observed for HgCl_2_ and organomercurials.^21^ Another possibility is that the MnCl_2_ employed may contain traces of Mn^+3^ or Mn^+4^, as described by Hem,^22^ which could produce deactivation by oxidizing Cys231 and/or other residues.

We went on to examine whether stabilization by divalent cations is unique to *Torpedo* AChE, or also holds true for other AChEs. The three AChEs examined were zebrafish, *Danio rerio*, AChE (*Dr*AChE), *Electrophorus electricus* (*Ee*) AChE, and HuAChE. Since all three are substantially more heat‐stable than *Tc*AChE, the thermal deactivation experiments were performed at 47°C for *Dr*AChE, and at 49°C for *Ee*AChE and HuAChE. Both Mg^+2^ and Mn^+2^, at 1 mM, substantially protected *Dr*AChE against thermal inactivation. Thus, whereas in their absence, ~90% inactivation occurred within 5 min, in the presence of either divalent ion, only ~10% inactivation occurred within 10 min (not shown). However, neither Mg^+2^ nor Mn^+2^ provided significant protection against thermal inactivation of either HuAChE or *Ee*AChE (not shown). As was observed for *Tc*AChE, Mn^+2^, in this case at concentrations above 1.8 mM, inactivates *Ee*AChE. The *Electrophorus* enzyme, unlike *Tc*AChE, is devoid of free sulfhydryl groups^23^; thus, its inactivation at high Mn^+2^ concentrations can most likely be ascribed to oxidation of other residues by traces of Mn^+3^ or Mn^+4^.

### 2.3 *Electron paramagnetic resonance measurements*

**FIGURE 2:**
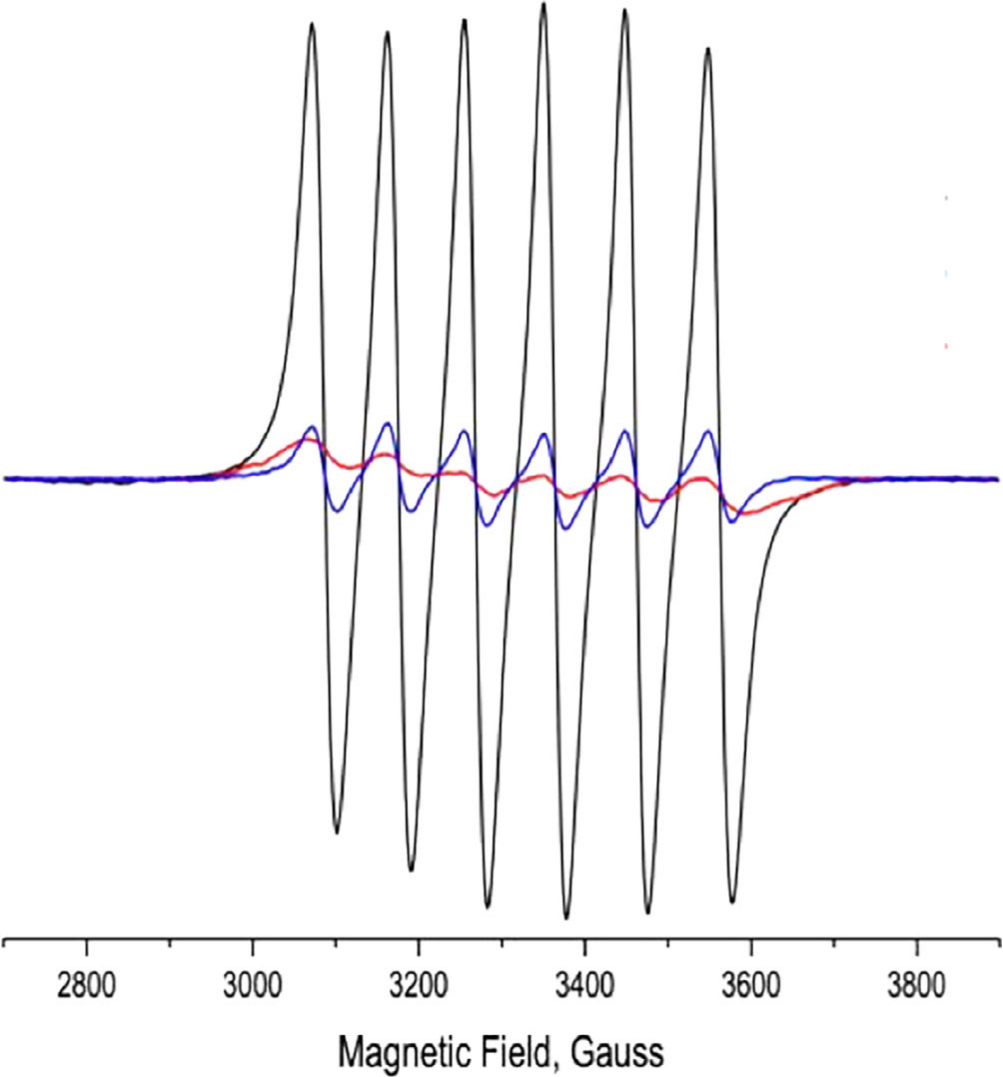
Effect of *Tc*AChE on the electron paramagnetic resonance (EPR) spectrum of Mn^+2^. 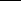, 10^−5^ M Mn^+2^; 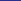, 10^−5^ M Mn^+2^ + 7×10^−6^ M *Tc*AChE; 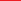, 10^−5^ M Mn^+2^ + 3×10^−5^ M *Tc*AChE

Figure 2 shows the electron paramagnetic resonance (EPR) spectrum of 10 μM MnCl_2_ in 0.1 M potassium phosphate, pH 7.4, at room temperature, and the spectra obtained when the solution contained, in addition, two different concentrations of *Tc*AChE. It can be seen that the intensity of the EPR signal of Mn^+2^ decreases dramatically upon addition of the enzyme. At saturation, the signal is ~5% of the value for the free metal ion.

**FIGURE 3:**
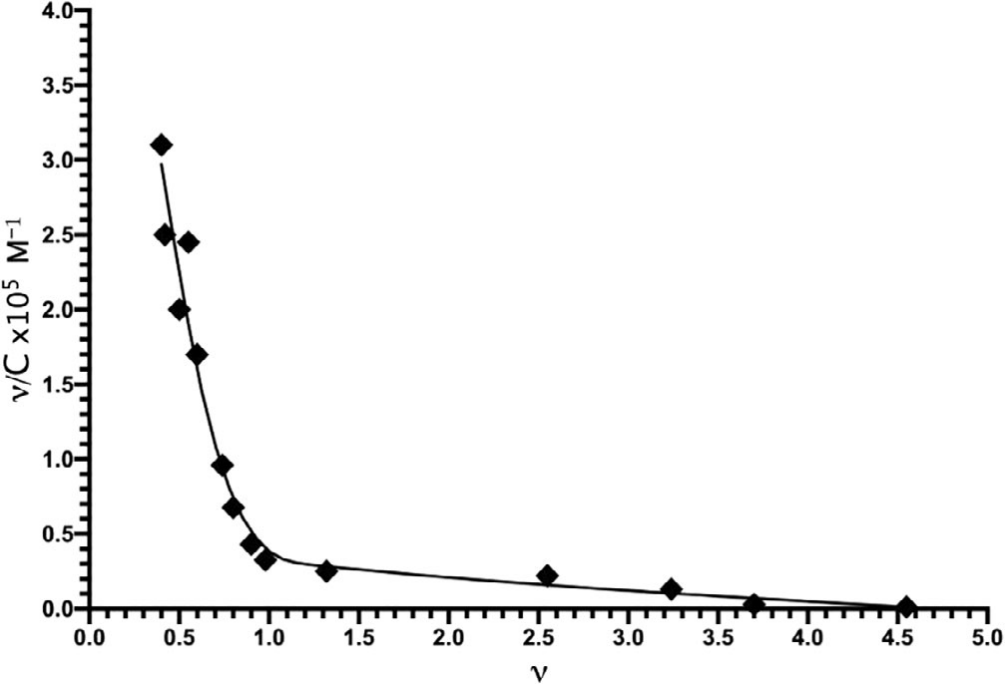
Scatchard plot of the interaction of Mn^+2^ with *Tc*AChE constructed on the basis of the titration of the metal ion with the enzyme as monitored by electron paramagnetic resonance (EPR), in accordance with Equation (1). The data points displayed were acquired in four separate experiments. *ν* = [Mn^+2^]_bound_/[*Tc*AChE]; *ν*/C = [Mn^+2^]_bound_/([Mn^+2^]_free_[*Tc*AChE])×10^5^; C is the molar concentration of the free Mn^+2^

In order to obtain a Scatchard plot, a solution of 20 μM *Tc*AChE in 0.1 M potassium phosphate, pH 7.4, was titrated with 4–280 μM MnCl_2_ in the same buffer, at room temperature. The Scatchard plot shown in Figure 3 is a combination of two segments that appear to display multitype binding. The steeper segment was constructed by linear regression of seven data points spanning *ν* values of 0.4–0.9. Its extrapolated intersection with the abscissa yields a value of *n* = 0.97. Thus, there is a single high‐affinity binding site, with an association constant, K_ass_, of 6×10^5^ M^−1^. The second segment, which extrapolates to *ν* = 3.5–4.5 on the abscissa, indicates an interaction with four to five weaker binding sites per *Tc*AChE catalytic subunit, with K_ass_ values in the range of 7×10^3^ M^−1^. It should be noted that the Mn^+2^ concentration required to confer 50% of maximal protection against thermal inactivation of *Tc*AChE was ~400 μM (Figure 1). This relatively high value suggests involvement of its low affinity binding sites, in addition to the high affinity site, in the observed stabilization by Mn^+2^. More experiments will be required to clarify this issue.

In studies on the interaction of the PAS probe, propidium, with *Tc*AChE, it was shown that Ca^+2^ and Mg^+2^ both compete with propidium, displaying association constants, K_ass_, of 2.30×10^3^ and 1.33×10^3^ M^−1^, respectively.^24^ Their affinities are thus much lower than that for the high‐affinity binding site revealed by the Scatchard plot, but their site of interaction might be one of the weak affinity sites.^24^

### 2.4 *DSC measurements*

**FIGURE 4:**
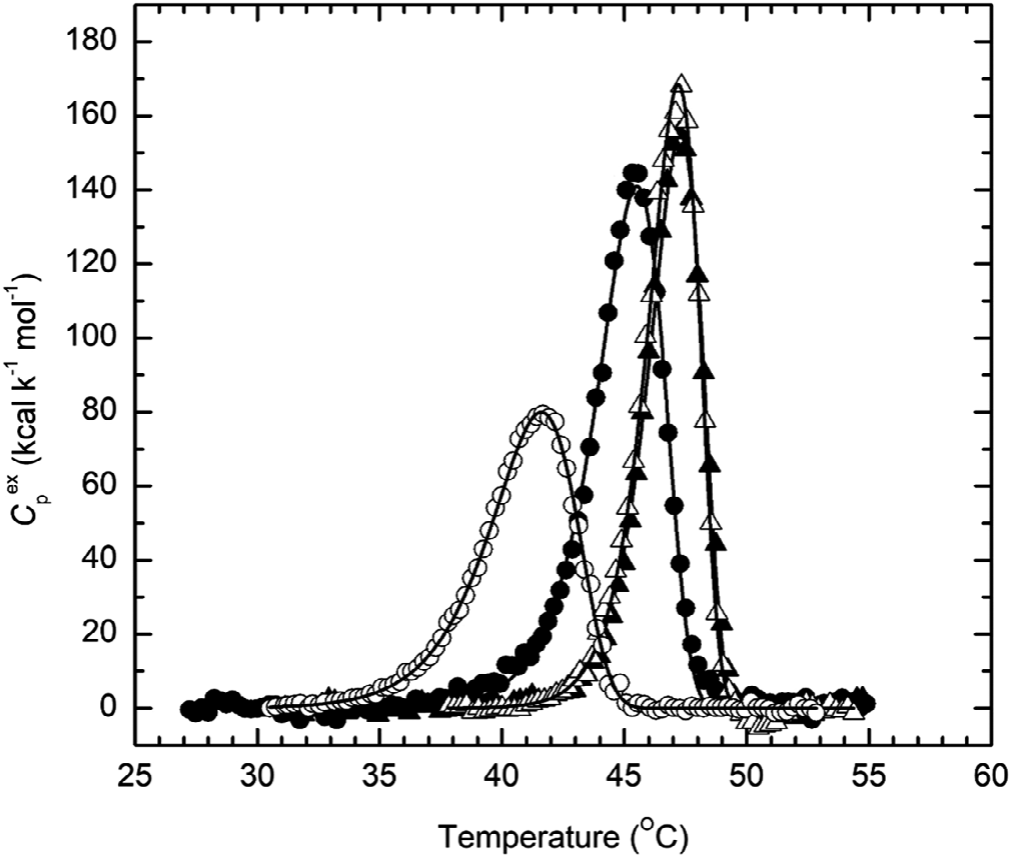
Temperature dependence of the excess molar heat capacity of *Tc*AChE in the absence and presence of divalent metal ions. ○‐○‐○, apo*Tc*AChE; ●‐●‐●, *Tc*AChE + 10 mM CaCl_2_; Δ‐Δ‐Δ, *Tc*AChE + 10 mM MgCl_2_; ▴ ‐ ▴ ‐ ▴, *Tc*AChE + 10 mM MnCl_2_. The scan rate was 1.0 K/min. Solid lines are the best fits obtained by applying Equation (3) to the experimental curves. The *Tc*AChE concentration was 6.3 μM in 0.1 M NaCl/10 mM HEPES, pH 7.5

Stabilization by divalent cations was further characterized by high‐sensitivity DSC. Figure 4 shows the excess molar heat capacities obtained by DSC in the presence and absence of the three divalent cations studied, Ca^+2^, Mg^+2^, and Mn^+2^. The apparent *T*_m_, namely, the temperature at the maximum of the heat capacity profile, is found to be raised in the presence of all three divalent cations, and it is seen that the thermal transition becomes more cooperative in the presence of the cations than in their absence.

2.5

Whether in the presence or absence of the divalent cation, denaturation is always calorimetrically irreversible, because no thermal effect is observed on heating the enzyme solution a second time (not shown). The effect of scan rate on all three calorimetric profiles (not shown) clearly indicates that they represent irreversible, kinetically controlled transitions, as earlier shown for DSC in the absence of divalent ions.^15^ For this reason, analysis of DSC transitions on the basis of equilibrium thermodynamics was ruled out,^25^ and was performed as described earlier,^15^, ^26^ using the simple two‐state irreversible model (see Section 4). As already mentioned, all three divalent cations strongly increase the thermostability of the *Tc*AChE (Figures 1 and 4). Furthermore, the Arrhenius activation energy for *Tc*AChE in the presence of the divalent cations (Table 2) is much higher than in their absence, and the thermodynamic parameters for the thermal transition are substantially increased (Table 3).

**TABLE 2:** Arrhenius equation parameter estimates for *Tc*AChE in the presence and absence of divalent cations assuming a two‐state irreversible model

	apo *Tc*AChE	+ 10 mM CaCl_2_	+10 mM MnCl_2_	+10 mM MgCl_2_
T*, K	315.6	319.0	320.6	320.3
*E*_A_, kcal mol^−1^	111.1	148.6	191.1	198.4
*r* ^a^	0.9992	0.9978	0.9986	0.999

^a^ The correlation coefficient (*r*) was calculated as $r=\sqrt{1-\sum_{i=1}^{n}{\left(y_{i}-y_{i}^{\text{calc}}\right)}^{2}/\sum_{i=1}^{n}{\left(y_{i}-y_{i}^{m}\right)}^{2}}$, where $y_{i}$ and $\overset{\mathrm{calc}}{\underset{i}{y}}$ are, respectively, the experimental and calculated values of the excess heat capacity, $\overset{\mathrm{ex}}{\underset{p}{C}}$, $\overset{m}{\underset{i}{y}}$ is the mean of the experimental values of $\overset{\mathrm{ex}}{\underset{p}{C}}$, and *n* is the number of points.

**TABLE 3:** Eyring equation parameter estimates for a two‐state irreversible model of thermal denaturation of native *Tc*AChE and of its complexes with divalent metal ions at 25°C

	∆*H* ^*#*^ (kcal mol^−1^)	∆*S* ^#^ (cal K^−1^ mol^−1^)	∆*G* ^#^ (kcal mol^−1^)
apo *Tc*AChE	110.5	291.1	23.6
+10 mM CaCl_2_	148.0	405.1	27.2
+10 mM MnCl_2_	190.5	535.6	30.8
+10 mM MgCl_2_	197.8	558.7	31.2

Although Mn^+2^ stabilizes *Tc*AChE considerably more than Mg^+2^ in the thermal denaturation experiments displayed in Figure 1, the values of *E*_*A*_, *∆H* ^*#*^, *∆S* ^*#*^, and *∆G* ^*#*^ shown for the two ions in Tables 2 and 3 are quite similar. The calorimetric experiments were performed at 10mM concentrations of both Mg^+2^ and Mn^+2^. Such high concentrations of Mn^+2^ were shown to inactivate *Tc*AChE (see above). It is thus possible that traces of Mn^+3^ and/or Mn^+4^ convert the native enzyme to a partially unfolded state by oxidation already prior to commencement of the calorimetric scan, as was found to occur under oxidative stress.^27^

### 2.6 *Structural studies*

**FIGURE 5:**
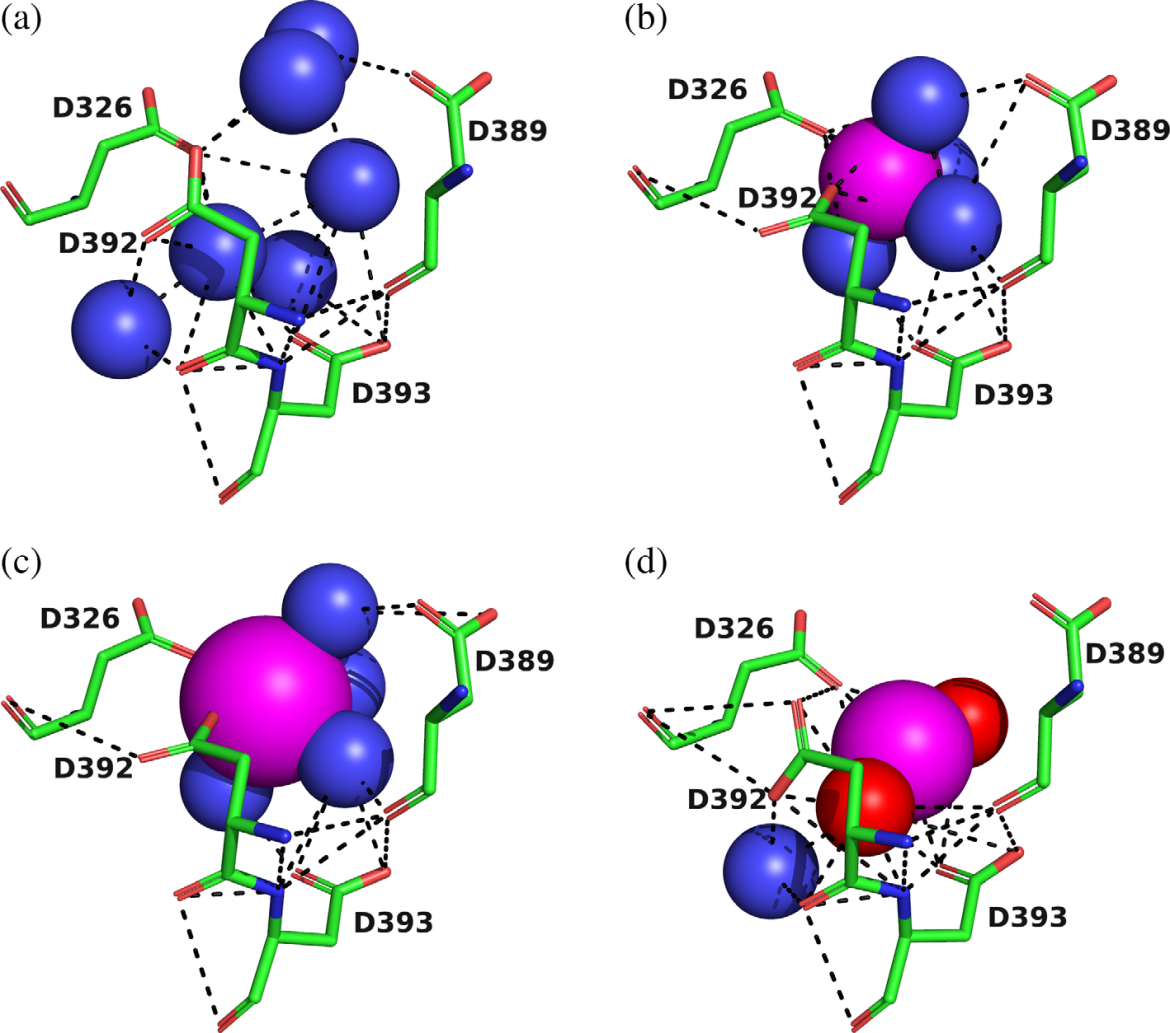
4D motif in *Tc*AChE. The four Asp residues, D326, D389, D392, and D393, are shown as sticks, with carbons in green, oxygens in red, and nitrogens in blue. Solvent waters are shown as blue spheres, and the metal ions as magenta spheres, with their sizes proportionate to their Van der Waals radii; the oxygens of the uranyl moiety are shown as red spheres. Noncovalent hydrogen bonds and ionic bonds are shown as dashed black lines. (a) Apo *Tc*AChE; (b) Mg^+2^/*Tc*AChE; (c) Ca^+2^/*Tc*AChE; and (d) UO_2_^+2^/*T*cAChE

In order to investigate the structural basis for the strong stabilization of *Tc*AChE by divalent ions, trigonal crystals of the complexes of the enzyme with Mg^+2^ and Ca^+2^ were obtained by crystallization of the native enzyme, using conditions containing high concentrations of magnesium acetate and calcium acetate, respectively (see Section 4). Solution of the structures by molecular replacement reveals, in both cases, a divalent cation located in a negatively charged pocket that is formed by the side chains of four aspartate residues, D326, D389, D392, and D393, which we call the 4D motif. Interestingly, this binding site is identical with that for the UO_2_^+2^ ion in the heavy atom derivative of the trigonal crystal form that was one of two used to solve the structure of *Tc*AChE.^18^ The Mg^+2^/*Tc*AChE complex contained, in addition, 3 Mg^+2^ ions and a single Zn^+2^ ion, all located on the surface of the enzyme, remote from the 4D site. This is in agreement with the ESR data that revealed one high affinity site for Mn^+2^, and three to five low affinity sites (Figure 3). Figure 5 shows the negatively charged pocket in the native enzyme, and in the UO_2_^+2^, Mg^+2^, and Ca^+2^ complexes. In the native crystal structure, several waters can be detected that form H‐bonds with the four Asp residues and with each other (Figure 5a). In the uranyl complex, the UO_2_^+2^ ion H‐bonds, via its oxygen atoms, to D326, D392, and D393, and indirectly, via a water to D389. In both the Mg^+2^ and Ca^+2^ complexes, the divalent ion is surrounded by several waters, and a complex array of ionic interactions and H‐bonds is formed, which is consistent with the large thermal stabilization observed experimentally. In both cases, the metal ion interacts with two of the Asp residues directly, D326 and D392, and with two indirectly, via waters, D389 and D393. It should be noted that this binding site for divalent cations is different from the EF‐hand identified in *Tc*AChE, which it had earlier been suggested might serve as a binding site for Ca^+2^ ions.^17^

**FIGURE 6:**
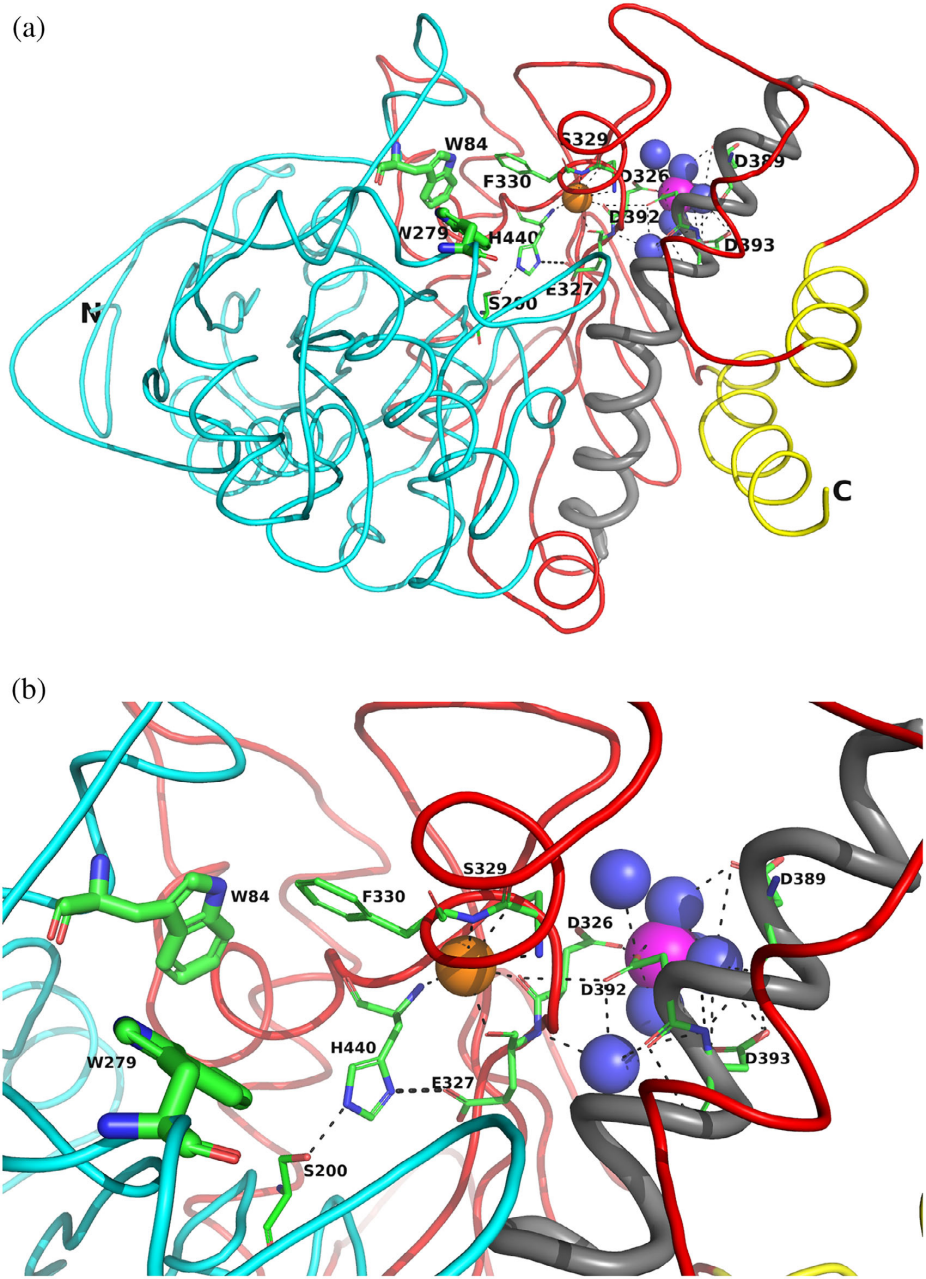
Overall views of the Mg^+2^/*Tc*AChE complex. (a) Ribbon diagram of the Mg^+2^/*Tc*AChE complex. The representation shows the entire structure, with the first subdomain, residues 4–305, in cyan, and the second, residues 306–535, in red. It is oriented looking into the active‐site gorge, with W279, in the peripheral anionic site (PAS), at the top of the gorge, and W84, in the catalytic anionic site (CAS) toward the back, adjacent to the catalytic triad, S200‐E327‐H440. All these residues are depicted as sticks. The long α‐helix, N383‐K413, against which the 4D pocket is glued, is in grey, and the two helices that contribute to the four‐helix bundle of the dimer, D365‐Y375 and V518‐T535, are in yellow. The Mg^+2^ in the 4D pocket is in magenta, and is surrounded by four waters in blue. A conserved water H‐bonds with D326, of the 4D motif, with E327 and H440, in the catalytic triad, and with the main‐chain nitrogen of F330, which, in turn, contributes to the CAS. This water which is homologous to water 623 in Koellner et al.,^29^ is shown as an orange sphere. (b) Close up, with the same orientation, showing the interactions of the active site, the 4D pocket, and the conserved water, shown as an orange sphere

However, looking at the broader picture, one can ask how does the presence of the divalent ion stabilize the whole structure, including the active‐site gorge, and, within it, the active site itself, which is, at first glance, quite distant from it. Thus, the distance from the divalent ion, Mg^+2^, to S200*O*γ is 16.4 Å. In Figure 6, the *Tc*AChE monomer is oriented such that the active‐site gorge is vertical, and the catalytic triad residues are near the bottom at the right, and beyond them, further to the right, the Ca^+2^/Mg^+2^ binding pocket. On one face of the pocket, D389 and D393 are glued to the long bent α‐helix that stretches from residue N383 to residue K413. This long helix is, in turn, linked to the four‐helix bundle at the dimer interface. On the other side of the pocket, one of the four Asp residues, D326, is adjacent to E327, which points away from the binding pocket into the active‐site gorge, being part of the catalytic triad, S200‐E327‐H440.^18^ Since S200 is in the first subdomain of the enzyme,^28^ there is a chain of interactions stretching from the four‐helix bundle via the divalent ion binding pocket across the active‐site gorge. An important stabilizing element is a conserved water, water 623 in the study of Koellner et al.,^29^ which makes H‐bonds with D326, of the 4D motif, with E327 and H440, in the catalytic triad, and with the main‐chain nitrogen of F330, which, in turn, contributes to the CAS that binds ACh. It should be noted that both Mg^+2^ and Mn^+2^ produce much larger thermal stabilization than Ca^+2^. This may be ascribed to their smaller radii, 1.73 Å, and 1.97 Å, respectively, as compared to 2.31 Å for Ca^+2^, resulting in formation of a more tightly packed complex within the pocket formed by the four Asp residues.

Already in our earlier study,^16^ we suggested that the divalent metal ion might be acting as a chaperone that would assist the folding of the large AChE polypeptide chain, just as pharmacological chaperones, acting at lower concentrations,^30^ and chemical chaperones, acting at much higher concentrations,^31^ both promote folding. Similarly, it was suggested that reversible AChE inhibitors could serve as pharmacological chaperones to promote folding of the enzyme.^32^ Intracellular Mg^+2^ concentrations are, in general, of the order of 30 mM, though much of the ion is complexed.^33^ It is thus plausible that Mg^+2^ may assist the folding of newly synthesized *Tc*AChE. The purified *Tc*AChE dimer employed in this study is derived from the GPI‐anchored dimer by solubilization with bacterial PI‐specific phospholipase C, followed by purification by affinity chromatography.^34^, ^35^ *In situ*, in the electric organ of *T*. *californica*, it is anchored to the presynaptic membrane on the outer surface of the electroplaque.^36^ Electrophysiological elasmobranch medium contains 4.4 mM CaCl_2_ and 1.3 mM MgCl_2_.^37^ Thus, it is plausible that the 4D motif is still partially or fully occupied by one or the other of the divalent metal ions. However, the thermal inactivation experiments described above indicated affinities for both Ca^+2^ and Mg^+2^ in the millimolar range. Thus, it is not surprising that the native dimer, which is purified by affinity chromatography in their absence, displays an empty 4D pocket (Figure 5a).

Since occupancy of the 4D pocket by a divalent ion stabilizes the protein, we considered the possibility that engineering a 4D motif would allow stabilization of the protein by addition of a divalent metal ion. However, insertion of the 4D motif itself should result in destabilization due to electrostatic repulsion within the motif, so that no gain in stabilization would be produced by the metal ion. We examined the putative destabilization by the 4D motif by applying to native *Tc*AChE the PROSS algorithm, which predicts mutations that will enhance the stability of a given protein.^38^ It was found that D392 is mutated to Ile in all nine designed mutant proteins generated by PROSS, indicating that the 4D motif is a destabilizing entity in the absence of a divalent metal ion.

### 2.7 *Bioinformatic analysis*

**FIGURE 7:**
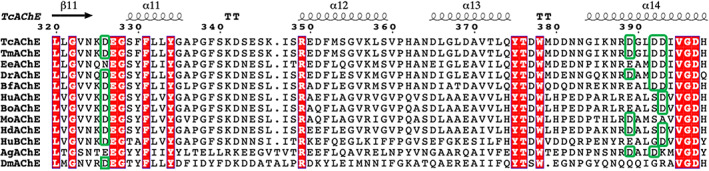
Sequence alignments of residues 320–400 in several AChEs and in HuBChE. The numbering used is that of *Tc*AChE. Fully conserved residues are in white on a red background. The columns for the four residues corresponding to the 4D motif in *Tc*AChE and zebrafish AChE are framed in green, and it can be seen that the motif is conserved only in these three AChEs. *Tc*AChE, *Torpedo californica* AChE; *Tm*AChE, *Torpedo marmorata* AChE; *Ee*AChE, *Electrophorus electricus* AChE; *Dr*AChE, *Danio rerio* AChE; *Bf*AChE, *Bungarus fasciatus* AChE; HuAChE, human AChE; BoAChE, bovine AChE; MoAChE, mouse AChE; HdAChE, designed HuAChE, D4 variant^38^; HuBChE, human BChE; AgAChE, *Anopheles gambiae* AChE; *Dm*AChE, *Drosophila melanogaster* AChE

Sequence alignment, utilizing MultAlin^39^ and ESPript,^40^ shows that, among several AChE and BChE sequences compared, the 4D motif, consisting of the four Asp residues ‐ D326, D389, D392, and D393 ‐ involved in binding the divalent metal ions, is seen in *Tc*AChE, in the closely homologous *Torpedo marmorata* AChE, and in zebrafish (*D*. *rerio*) AChE (Figure 7). Overall, 531 AChE sequences and 90 BChE sequences are present in the ESTHER database <http://bioweb.supagro.inra.fr/ESTHER/general?what=index>.^41^ The DDDD motif was identified in 31 AChE sequences, and in 28 BChE sequences.

The thermal stability data presented above for *Dr*AChE, *Ee*AChE and HuAChE showed that while *Dr*AChE was significantly stabilized by Mg^+2^ or Mn^+2^, the other two were not. Thus, it is plausible that binding of the divalent metal ions by the 4D motif present in *Dr*AChE is responsible for the stabilization observed, as is the case for *Tc*AChE. In *Ee*AChE, the four corresponding residues are D, D, E and N, and in HuAChE D, H, E, and D (in both cases using *Torpedo* numbering). Thus, it is not surprising that no significant thermal stabilization is produced in either by the divalent metal ions. In *Bungarus fasciatus* (*Bf*) AChE, the corresponding residues are D, E, D, and D (again, using *Torpedo* numbering), and it will be interesting to check whether it is stabilized by divalent metal ions.

**FIGURE 8:**
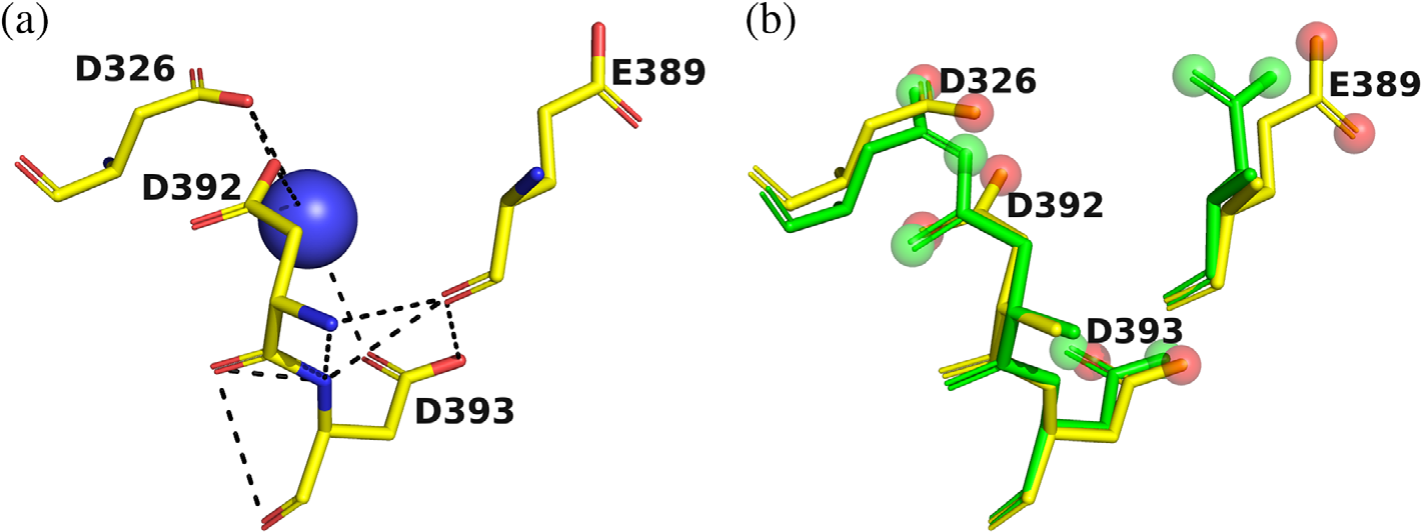
Pocket in *Bf*AChE that is homologous to the 4D pocket in *Tc*AChE. (a) Crystal structure^43^ (PDB‐ID 4qww), showing three Asp residues, a Glu residue, and a water. (b) Overlay of the *Bf*AChE pocket (yellow sticks) on the *Tc*AChE pocket (green sticks) with the distal oxygens displayed as red and green balls, respectively

It was recently reported that neither Ca^+2^ nor Mg^+2^ could be detected in crystals of AChE, despite their presence in the mother liquor (Pascale Marchot, personal data, quoted in Comoletti et al.^42^). Since these data were obtained by Marchot, they most likely refer to soaking trials performed on crystals of MoAChE, or possibly of *Ee*AChE or of *Bf*AChE. The alignments displayed in Figure 7 show that the residues in MoAChE and *Ee*AChE corresponding to the 4D motif in *Torpedo* are DDSA and NEDD, respectively. Thus, MoAChE, in particular, and *Ee*AChE, are unlikely to bind the divalent metal ions. As just pointed out, the most similar motif is that in *Bf*AChE, DEDD, for which the crystal structure is available.^43^ If we overlay this motif on the 4D motif in *Tc*AChE, we see that E389 (*Torpedo* and *Bungarus* numbering are serendipitously identical for these residues) points away from the pocket, and would not interact with a divalent ion if present (Figure 8). However, the remaining 3D motif has the potential to bind divalent metal ions, and this issue should be examined experimentally.

We wished to find out whether the 4D motif also serves as a divalent metal ion binding motif in other proteins. Numerous groups have adopted proteomic approaches to identify or categorize binding motifs and sites for metal ions in proteins; for some examples, see References ^44^, ^45^, ^46^, ^47^, ^48^, ^49^, ^50^ However, we chose to address the issue by searching for the 4D motif making use of the ASSAM server (http://27.126.156.175/assam), ASSAM being the acronym for “Amino acid pattern Search for Substructures And Motifs.”^51^, ^52^ The 4D motif is chiral. Consequently, when we interrogated the ASSAM server with the 4D motif from the apo *Tc*AChE structure [PDB‐ID 1ea5], using as the search motif the four Asp residues, D326, D389, D392, D393, it searched for both right‐handed and left‐handed superpositions. In each case, it retrieved the first 100 structures from the PDB, ordered starting with the lowest RMSD (Table S1). In the right‐handed superposition search, the first hit (PDB‐ID 6g1u) was that of the complex of *Tc*AChE with an analog of tacrine,^53^ the first AChE inhibitor approved for treatment of Alzheimer's disease.^54^ No additional *Tc*AChE structures were retrieved, since the option to exclude redundant structures was chosen while using ASSAM. It is of interest that, in the list of left‐handed superpositions, ASSAM again retrieved PDB‐ID 6g1u, in this case as the third hit, that is, with the third lowest RMSD, but with the residues matching in a different order, namely, D326, D393, D392, D389, rather than D326, D389, D392, D393, which was the order in the search motif (Table S1). It should be stressed that the ASSAM algorithm was used since it optimizes the fit of the side chains of the search motif, independent of the position and orientation of the main‐chain atoms. This is due to the representation of each amino acid as a vector between two pseudo‐atoms within the side chain.^52^ In the case of aspartate, the pseudo‐atoms representing the side chain go from Cβ to the midpoint of Oδ1/Oδ2. This is preferable to an all atom representation, inasmuch as ASSAM is able to identify many 4D motifs in which the carboxylates align well with the 4D motif of *Tc*AChE, without requiring all the atoms of the main chain to align so well.^52^

As just mentioned, each superposition retrieved 100 proteins, with a broad repertoire of structures and functions (Table S1). In many of these structures (>60%), a divalent metal ion is present within the 4D motif, mostly Ca^+2^, Mg^+2^, Mn^+2^, and sometimes Zn^+2^; in a few cases it contains a K^+^ or Na^+^ ion, and in about 7% it is denoted as unoccupied. However, ASSAM also retrieves structures in which the 4D motif is present, together with a ligand, but the ligand is not associated with the motif. These include, for example, the *Tc*AChE structure at the top of our list (PDB‐ID 6g1u), two structures that contain sulfate ions (PDB‐IDs 5iz5 and 5ghr), and a structure that contains flavin adenine dinucleotide (PDB‐ID 5i3d). So the percentage of protein structures in which the 4D motif is present, but is not occupied, is actually far higher than 7%. It should also be noted that in some of the PDB structures retrieved, even when a divalent ion is present, not all four members of the 4D motif are involved in interaction with it, whether directly or through waters.

**FIGURE 9:**
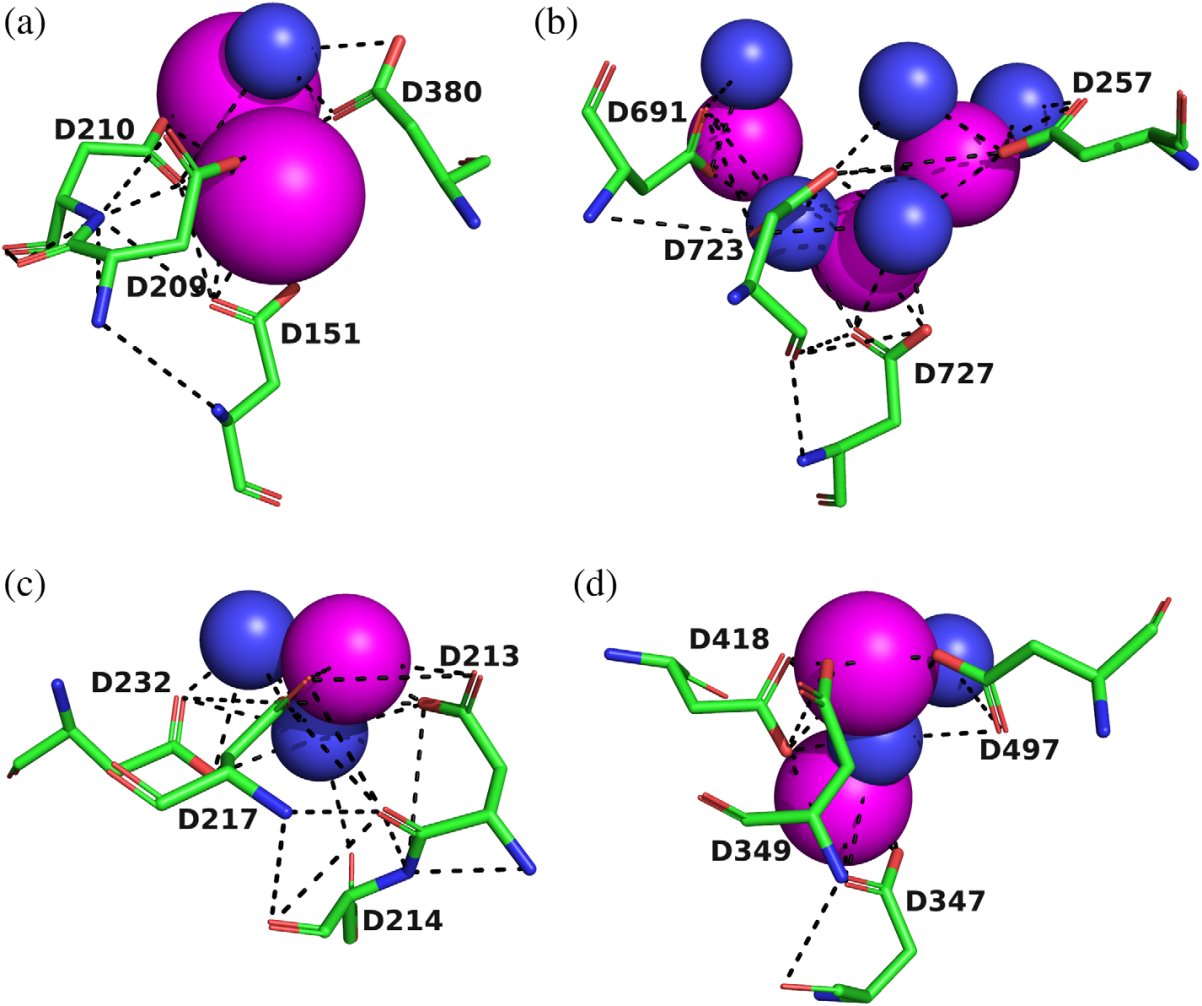
4D motifs in four proteins retrieved from the ASSAM server. The four Asp residues are shown as sticks, with carbons in green, oxygens in red, and nitrogens in blue. Solvent waters are shown as blue spheres, and the metal ions as magenta spheres, with their sizes proportionate to their Van der Waals radii. Noncovalent H‐bonds and ionic bonds are displayed as dashed black lines. (a) *Bacillus subtilis* phosphodiesterase PhoD,^55^ containing two Ca^+2^ ions (PDB‐ID 2yeq); (b) proton pyrophosphatase,^56^ containing three Mg^+^ ions (PDB‐ID 4a01); (c) *Streptococcus uberis* geranylgeranyl diphosphate synthase,^57^ containing one Mg^+2^ ion (PDB‐ID 4lfg); (d) phosphodiesterase acting on cyclic dinucleotides,^58^ containing two Mn^+2^ ions (PDB‐ID 5xsp)

As stated above, multiple structures were identified that contain one of the three divalent ions that were shown to interact with *Tc*AChE, namely, Ca^+2^, Mg^+2^, and Mn^+2^. The 4D motifs of four such structures are shown in Figure 9. These are of *Bacillus subtilis* phosphodiesterase PhoD, containing two Ca^+2^ ions^55^ (PDB‐ID 2yeq, Figure 9a; proton pyrophosphatase, containing 3 Mg^+2^ ions^56^ (PDB‐ID 4a01, Figure 9b); *Streptococcus uberis* geranylgeranyl diphosphate synthase, containing one Mg^+2^ ion^57^ (PDB‐ID 4lfg, Figure 9c); and phosphodiesterase acting on cyclic dinucleotides, containing two Mn^+2^ ions^58^ (PDB‐ID 5xsp, Figure 9d).

**FIGURE 10:**
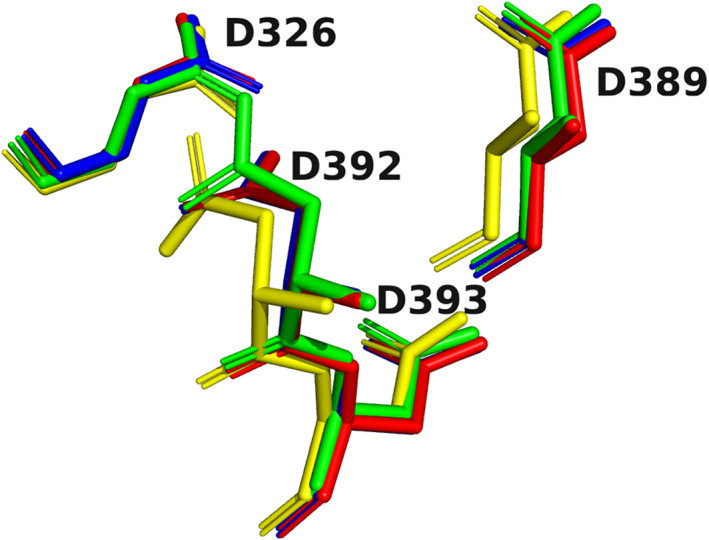
Overlays of the 4D motifs displayed in Figure 5. All metal ions and waters are removed, and only the Asp residues are displayed in stick format. Apo *Tc*AChE in green; Ca^+2^/*Tc*AChE in red; Mg^+2^/*Tc*AChE in blue; UO_2_^+2^/*Tc*AChE in yellow

**FIGURE 11:**
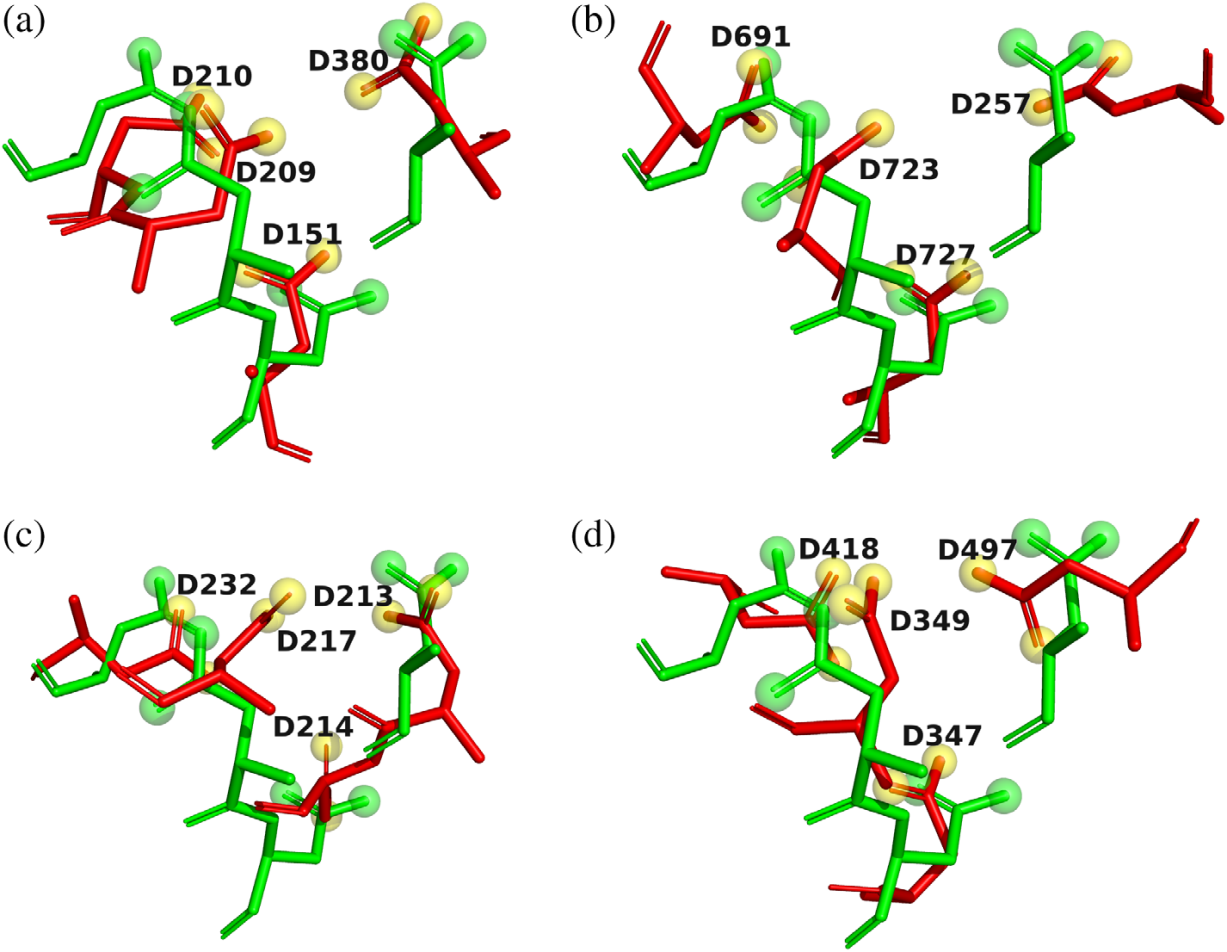
Overlays on Apo *Tc*AChE of the 4D motifs of the four proteins retrieved from the ASSAM server displayed in Figure 8. All metal ions and waters are removed, and only the Asp residues are displayed in stick format. Apo *Tc*AChE is displayed as green sticks, and the retrieved proteins as red sticks, with the distal oxygens shown as green and yellow balls, respectively. (a) *Bacillus subtilis* phosphodiesterase PhoD (PDB‐ID 2yeq); (b) proton pyrophosphatase (PDB‐ID 4a01); (c) *Streptococcus uberis* geranylgeranyl diphosphate synthase (PDB‐ID 4lfg); and (d) phosphodiesterase acting on cyclic dinucleotides (PDB‐ID 5xsp)

Already in the crystal structures of the complexes of the divalent cations with *Tc*AChE, and of that with the uranyl ion, as mentioned above, there is not a simple situation in which the metal ion interacts directly with all four Asp residues. In each case, there are not only direct interactions, but also indirect interactions, via water molecules. The cases shown in Figure 9 display even greater diversity. Thus, for example, in the proton pyrophosphatase crystal structure, three Mg^+2^ ions and five waters are embedded within the motif, even though it maintains essentially the same dimensions, and a very similar conformation, as when it contains a single divalent ion and less waters, or is only occupied by waters. This point is emphasized in Figures 10 and 11. In Figure 10, the four 4D pockets for *Tc*AChE shown in Figure 5 are overlayed on each other as stick models, with the metal ions and waters removed, so as to emphasize how well they superimpose. In Figure 11a–d, the pockets of each of the four proteins displayed in Figure 9 are overlayed on the 4D pocket of Apo *Tc*AChE (Figure 5a). Again, only the four Asp residues, in stick format, are retained, for both *Tc*AChE and for the other four proteins. The great similarity is reflected in the RMSD values of the four vectors between the pseudo‐atoms of each Asp side chain, relative to *Tc*AChE. These are 1.21, 1.20, 1.24, and 1.40 Å, respectively, for the four pockets overlayed on that of native *Tc*AChE in Figure 11. Thus, the 4D motif differs greatly from the two well‐known motifs referred to in the Introduction, the Ca^+2^‐binding EF hand motif, and the zinc finger. Both these motifs are compact structures, in which the sequence folds back on itself, the amino residues involved are adjacent to each other, *that is*, relatively close in the sequence, and only the divalent ion involved is bound. In the case of the 4D motif, residues may be recruited that are quite distant from each other ‐ for example, D326, D389, D392, and D393 in *Tc*AChE; D257, D691, D723, and D727 in proton pyrophosphatase; D347, D349, D418, and D497 in phosphodiesterase acting on cyclic nucleotides. Thus, the 4D motif displays great versatility in having the capacity to bind a repertoire of combinations of metal ions and waters, while retaining its own conformation, with only minor rotations of the carboxyl groups occurring. To the best of our knowledge, a structural motif displaying such versatility has not been described before.

One of the referees suggested that we should extend our bioinformatic analysis to, for example, (3D)E motifs. Such an example already exists ‐ the DEDD motif in *Bf*AChE, which was described above (Figure 8). If we perform an ASSAM search using the DEDD motif in *Bf*AChE, about 40% of the 200 structures retrieved contain a divalent metal ion (Table S2). As is the case for the 4D motif, these are mostly Ca^+2^, Mg^+2^, or Mn^+2^. In many of these structures, the side chain of the Glu residue points away from the metal ion, as was seen in the *Bf*AChE structure (Figure 8). Thus, effectively, what we have is a 3D divalent metal ion‐binding motif, which we are currently investigating.

## 3 CONCLUSIONS

The divalent metal ions, Mg^+2^, Ca^+2^, and Mn^+2^, were shown to stabilize the enzymic activity of *Tc*AChE against thermal deactivation. EPR spectroscopy revealed a single strong binding site for Mn^+2^ in the enzyme. Differential scanning calorimetry (DSC) revealed that all three divalent ions raise both the activation energy and temperature of the thermal transition, and increase its cooperativity.

Solution of the crystal structures of the complexes of *Tc*AChE with Mg^+2^ and Ca^+2^ revealed a binding pocket for both these ions which contains four aspartate residues, and is, therefore, called the 4D motif. Both complexes include also four waters in the binding pocket. The bound Mg^+2^ and Ca^+2^ ions make direct contacts with two of the Asp residues, and indirect contacts, via waters, with the other two. Sequence alignment identified the 4D motif also in zebrafish AChE (*Dr*AChE). Zebrafish AChE (*D*rAChE) was shown to be similarly stabilized by divalent metal ions, in contrast to other AChEs that lack the motif.

Use of the ASSAM program, which identifies homologous motifs, retrieved ~200 proteins bearing 4D motifs among the crystal structures deposited in the PDB. In a substantial percentage of these structures, the motif is occupied by a divalent metal ion. Interestingly, even though the motif is strongly conserved in terms of RMSD values, it displays a versatile binding capacity, being seen to contain varying numbers of metal ions, 1–3, and one or several waters, in various crystal structures examined. Furthermore, the residues are not all adjacent within the primary sequence, often being widely separated. Thus, the 4D motif is a novel structural entity for binding divalent metal ions, with characteristics that differ greatly from those of such motifs as the zinc finger and EF‐hand, which were described previously.

## 4 MATERIALS AND METHODS

### 4.1 *Materials*

*Tc*AChE is the dimeric (G_2_) form prepared as described previously.^34^, ^35^ For the EPR experiments, the samples were first passed over a Chelex‐100 column to remove all traces of heavy metal ions.

*Electrophorus electricus* AChE (*Ee*AChE), Type V‐S (Catalog #2888), was purchased from Sigma (St. Louis, MO).

Human AChE (HuAChE) is a water‐soluble monomeric G_1_ form, expressed in HEK293T cells, from which it was secreted. The principal step in purification of the secreted enzyme involved an affinity column in which *m*‐[*ε*‐aminocaproyl‐*ε*‐aminocaproyl)‐aminophenyl‐trimethylammonium] was coupled to Sepharose 2B, the same affinity resin used for purification of G_2_ *Tc*AChE (see above and^35^). The purified enzyme has a specific activity of 420 units/nmol when assayed by the Ellman procedure at 25°C.

Adult zebrafish tissue, kindly provided by Takashi Kawashima (Department of Neurobiology, Weizmann Institute of Science), was stored at −20°C, and thawed immediately before use. The thermal inactivation experiments on zebrafish (*Dania rerio*) AChE, *Dr*AChE, utilized extracts of zebrafish tissue homogenized on ice in 0.5 M NaCl/50 mM Tris, pH 8.0, containing 0.1% Tergitol. These extracts displayed activity of ~3–4 units/ml when tested on ATC.

ATC iodide, 5,5′‐dithiobis(2‐nitrobenzoic acid), and Tergitol (Type NP‐10) were purchased from Sigma. Analytical grade MgCl_2_ and MnCl_2_ were purchased from Merck KgaA (Darmstadt, Germany), and analytical grade CaCl_2_ from JT Baker Chemicals (Phillipsburg, NJ). All other salts and buffers employed were also analytical grade.

### 4.2 *Assay methods*

AChE concentrations were determined spectrophotometrically, using a value of *ε*_280 nm_ (1 mg/ml) = 17.4.^59^ The *Tc*AChE concentration is expressed as the concentration of the dimer (molecular weight 130,000), assuming a subunit molecular weight of 65,000.^35^ AChE activity was monitored by the Ellman procedure,^60^ using ATC as the substrate. Measurements were performed in 0.01% sodium azide/0.1% Tergitol/200 mM NaCl/50 mM Tris, pH 8.0, at 25°C. The ATC concentration was determined by measurement of the absorption of the Ellman reagent after complete hydrolysis of the substrate by *Tc*AChE. It was about 80% of the nominal concentration based on weight.

Thermal inactivation experiments were performed as follows: *Tc*AChE samples, at 2×10^−9^ M, were incubated in 0.01% sodium azide/0.1% Tergitol/200 mM NaCl/50 mM Tris, pH 8.0, containing the appropriate concentration of the divalent metal ion. Loss of enzyme activity was monitored for greater than two half‐lives of inactivation, by 50‐ to 100‐fold dilution of aliquots into the Ellman reaction mixture, and assaying at 25°C, as above. The data points for the time‐course of residual activity were fitted to a mono‐exponential decay function (not shown), and the increases in *t*_1/2_ values were used to calculate the degree of enhancement of stability. Thermal inactivation of the other three AChEs was similarly monitored by dilution of suitable aliquots into the incubation buffer, with or without the divalent metal ion, which had been pre‐equilibrated at the appropriate reaction temperature. Thermal inactivation of *Tc*AChE was monitored at 39°C, of HuAChE and *Ee*AChE at 49°C, and of *Dr*AChE at 47°C.

### 4.3 *Electron paramagnetic resonance spectroscopy*

EPR spectra were recorded on a Bruker CW EPR spectrometer ELEXSYS‐500 at room temperature in a 110 μl flat quartz cell. The concentration utilized is that of the monomer, *i.e*., of the active sites. The EPR signals seen were primarily of the free Mn^2+^ ion, since the amplitude of the signal of the bound Mn^2+^ is ∼5% of that of an identical concentration of the free ion. A reference value was obtained by measuring the signal of free Mn^2+^, at a concentration of 1×10^−5^ M, at the start of each titration. The free Mn^2+^ concentration, and the number of bound equivalents, were calculated from the EPR signal intensity at each point, making use of a calibration curve obtained for the free Mn^2+^ ion.

The binding constant of Mn^+2^ for *Tc*AChE was calculated by constructing a Scatchard plot,^61^ according to the procedure of Danchin,^62^, ^63^ using the following equation:

(1) $$\nu /c=\left(n–\nu \right)K=\mathrm{nK}–\mathrm{\nu K}$$

where *ν* is the number of ligand molecules bound to the macromolecule, *c* is the concentration of the free ligand, *n* is the number of binding sites on the macromolecule, and *K* is the association constant of the ligand with the macromolecule.

If there is a single class of binding sites, a plot of *ν*/*c* versus *ν* should give a straight line, with a slope of –*K*, and the intercept on the x‐axis should correspond to the number of ligand‐binding sites on the macromolecule.

In our specific case, *ν* is the number of Mn^+2^ ions bound per *Tc*AChE catalytic subunit, *c* is the concentration of free Mn^+2^, *n* is the number of binding sites on the catalytic subunit, and *K* is the association constant.

### 4.4 *Differential scanning calorimetry*

DSC experiments were performed on a MicroCal MC‐2D differential scanning microcalorimeter (MicroCal Inc., Northampton, MA) with cell volumes of 1.19 ml, interfaced with a personal computer (IBM‐compatible) as described previously.^64^ Before measurement, sample and reference solutions were degassed in an evacuated chamber for 5 min at room temperature, and carefully loaded into the cells to avoid bubble formation. An overpressure of 2 atm of dry nitrogen was maintained over the liquids in the cells throughout the scans to prevent any degassing during heating. The reversibility of the thermal transitions was checked by examining the reproducibility of the calorimetric trace in a second heating of the sample immediately after cooling subsequent to the first scan. The experimental calorimetric traces were corrected for the effect of instrument response time using the procedure described previously.^65^

The excess molar heat capacity functions were plotted after normalization, taking the molecular mass of the *Tc*AChE dimer as 130 kDa,^35^ and chemical base line subtraction, using the Windows‐based software package (Origin) supplied by MicroCal.

In all cases, the thermal denaturation was found to be irreversible. In accordance with our earlier studies,^15^, ^16^, ^32^ only one model was considered in the analysis of the process of AChE denaturation. This was the simplest model, $N\overset{k}{\to }D$, which considers only two significantly populated macroscopic states, the initial or native state (*N*), and the final or denatured state (*D*), where *k* is a first‐order kinetic constant that changes with temperature, as given by the Arrhenius equation:

(2) $$k=\exp\left[\frac{E_{A}}{R}\left(\frac{1}{T^{*}}-\frac{1}{T}\right)\right]$$

where *E*_*A*_ is the activation energy of the denaturation process, *R* is the gas constant, and T* is the temperature at which *k* is equal to 1 min^−1^.

In this case, the excess heat capacity is given by the following equation^26^:

(3) $$C_{p}^{\mathrm{ex}}=\frac{1}{\nu }\mathrm{\Delta H}\;\exp\left\{\frac{E_{A}}{R}\left(\frac{1}{T^{*}}-\frac{1}{T}\right)\right\}\times \exp\left\{-\frac{1}{\nu }\int_{T_{o}}^{T}\exp\left[\frac{E_{A}}{R}\left(\frac{1}{T^{*}}-\frac{1}{T}\right)\right]\mathrm{dT}\right\}$$

where *ν* = *dT*/*dt* (K/min) is the scan rate value, and Δ*H* is the difference in enthalpy between the denatured and native states.

Thus, the thermal denaturation of *Tc*AChE can be described by a first‐order reaction. It can also be analyzed by use of the rate equation derived on the basis of conventional transition state theory.^66^ According to this theory, the rate constant is given by:

(4) $$k=\left(k_{B}T/h\right)\;\exp\left(\Delta S^{\#}/R\right)\;\exp\left(-\Delta H^{\#}/\mathrm{RT}\right)=\left(k_{B}T/h\right)\;\exp\left(-\Delta G^{\#}/\mathrm{RT}\right)$$

where *k*_*B*_ is the Boltzmann constant; *h* is the Planck constant; and Δ*S* ^*#*^, Δ*H* ^*#*^, and Δ*G* ^*#*^ are, respectively, the entropy, enthalpy, and standard molar Gibbs free energy of activation. Although the transition state theory is, strictly speaking, limited to gas‐phase reactions, plausible values for the effects of the divalent metal ions on Δ*G* ^*#*^ can be determined assuming a constant value of *k*_*B*_ *T*/*h*. Together with the values of *E*_A_ and T*, Δ*G* ^*#*^ calculated at any given temperature provides a satisfactory estimate of the thermal stability of the protein studied.

### 4.5 *Crystallography*

*Tc*AChE was purified as described under Materials. Trigonal crystals of space group P3_2_21 of the Mg^+2^/*Tc*AChE complex were obtained by vapor diffusion in hanging drops, at 20°C, by mixing 2 μl of protein (10–13 mg/ml in 0.1 M NaCl/0.1 M 2‐morpholinoethanesulfonic acid [MES]/0.02% sodium azide, pH 5.8) with 2 μl of precipitant solution (0.2 M magnesium acetate/10–15% (vol/vol) polyethylene glycol 5000 monomethyl ether, 0.1 M MES, pH 6.5), thus yielding crystals of the Mg^+2^/*Tc*AChE complex. Trigonal crystals of the Ca^+2^/*Tc*AChE complex, in the same space group, P3_2_21, were obtained under identical conditions, but using 0.2 M calcium acetate. Information concerning the native *Tc*AChE crystals, in space group P3_1_21, was presented earlier,^67^ as was the information for the UO_2_^+2^/*Tc*AChE complex, also in space group P3_1_21.^18^

Table 4 summarizes the statistics for data collection and structure refinement. For the Mg^+2^/*Tc*AChE and Ca^+2^/*Tc*AChE^*2*^ complexes, molecular replacement was done using the program AMoRE,^68^ taking the native *Tc*AChE structure (PDB‐ID 1EA5) as a starting model, after having omitted sugar and solvent molecules. Energy minimization, simulated annealing, individual thermal B‐factor refinement, and electron density map calculation were done using the program CNS.^69^ The graphic analyses were performed using TURBO‐FRODO.^70^ Further refinement of these two structures, and of the UO_2_^+2^/*Tc*AChE complex, were performed with Refmac,^71^ Coot^72^, ^73^ in the CCP4 software suite,^74^ and Phenix.^75^ In addition, the structures were checked and corrected with PDB_REDO.^76^

**TABLE 4:** Crystallographic data, data collection, and structure determination

	Mg^+2^/*Tc*AChE	Ca^+2^/*Tc*AChE	UO_2_^+2^/*Tc*AChE
Space group	P3_2_21	P3_2_21	P3_1_21
*a*, *b* (Å)	138.22	139.07	110.88
*c* (Å)	71.38	71.32	134.67
Resolution range (Å)	34.83–1.85	20.13–2.24	39.09–2.65
Beamline	ID14–EH2 of the ESRF	ID14–EH2 of the ESRF	RU‐300/Xentronics^a^
Temperature of data collection	155 K	155 K	RT (~20°C)
Unique reflections	66,953	38,704	23,867
Completeness (%) overall and (final shell)	99.6 (99.5)	99.35 (96.5)	84.23 (53.56)
Mean ((I)/sd(I)) overall and (final shell)	10.6 (2.5)	11.6 (2.5)	13.6 (1.44)
R‐factor^b^ (Rfree)	20.2 (24.1)	17.8 (22.5)	16.2 (21.2)
RMS deviation from ideality			
Bonds (Å)	0.007	0.008	0.012
Angles (°)	0.811	0.870	1.322
Average B‐factors (Å^2^)			
Protein	35.18	43.32	35.32
Water	40.14	44.37	26.32
PDB‐ID	7B38	7B8E	7B2W

Abbreviation: ESRF, European Synchrotron Radiation Facility.

^a^ X‐ray data were collected on a home source, a Rigaku RU‐300 rotating anode with a Xentronics area detector.

^b^ R‐factor = ∑_hkl_∥F_obs_∣‐∣F_calc_∥∕∑_hkl_∣F_obs_∣.

### 4.6 *Bioinformatics*

Multiple sequence alignment was performed utilizing *MultAlin* ^39^ together with *ESPript*.^40^ In order to search for 3D structural motifs the ASSAM server (http://27.126.156.175/assam) was employed, ASSAM being the acronym for “Amino Acid Pattern Search for Substructures And Motifs.”^51^, ^52^ Searches were done examining all 3D structures in the non‐redundant PDB (NR‐PDB) at 30% sequence identity cut‐off (excluding mutant structures).

For calculating RMSD values for motifs in two structures, ASSAM displays each amino acid as a vector for two representative pseudo‐atoms within the side chain. In the case of aspartate, the pseudo‐atoms representing the side chain go from Cβ to the midpoint of Oδ1/Oδ2.

### 4.7 *Databases*

The atomic coordinates and structure factors have been deposited in the Protein Data Bank, http://www.rcsb.org, for complexes of *T*. *californica* AChE with Ca^+2^, Mg^+2^, and UO_2_^+2^ with PDB‐IDs: 7B8E, 7B38, and 7B2W, respectively.

## CONFLICT OF INTEREST

The authors declare no conflict of interest.

## AUTHOR CONTRIBUTIONS

**Israel Silman**: Designed experiments and wrote the paper. **Valery L. Shynrov**: Performed the calorimetric experiments. **Yacov Ashani**: Performed the kinetic and thermal deactivation experiments. **Esther Roth**: Purified the *Torpedo* AChE. **Anne Nicolas**: Crystallized the enzyme complexes, collected the X‐ray data, and determined the crystal structures. **Joel L. Sussman**: Refined the crystal structures, performed the bioinformatic analyses, and wrote the paper. **Lev Weiner**: Designed the experiments, performed the electron paramagnetic resonance measurements, and wrote the paper.

## Supporting information


**Table S1** Output from the ASSAM server of proteins whose 3D structures contain the 4D motif, based on the crystal structure of apo *Tc*AChE.

**Table S2** Output from the ASSAM server of proteins whose 3D structures contains the 3D1E motif based on the crystal structure of *Bf*AChE.

**Table S3** List of all AChE and BChE sequences that contain the 4D motif.
