# Supplementary material for: Torpedo californica acetylcholinesterase is stabilized by binding of a divalent metal ion to a novel and versatile 4D motif
Source: Protein Sci. 2021 Mar 29;30(5):966–81. doi: 10.1002/pro.4061 (PMC8040873; doi:10.1002/pro.4061)
Supplement: Supplementary file 1 — Table S1 Output from the ASSAM server of proteins whose 3D structures contain the 4D motif, based on the crystal structure of apo TcAChE. [file PRO-30-966-s002.pdf]

GRaph theoretic Applications for Structure Searching

Molecular Function Regulation Lab.
M. Firdaus Raih Research Group
Universiti Kebangsaan Malaysia
The National University of Malaysia

KREBS INSTITUTE
The University Of Sheffield.

ASSAM
Amino acid pattern Search for Substructures And Motifs

IMAAAGINE SPRITE ASSAM NASSAM About GrASS Contact us HOME

Results of ASSAM search and righthanded superposition for 1ea5_4D
→ Download text version of the ASSAM output

Matches found in 1ea5_4D (PDB ID)	Description	Residues	Residue Matches Query		Database Hits	Heteroatoms Notes in Database hit	RMSD	Viewer
6g1u<br>PDB<br>PDBsum	ACETYLCHOLINESTERASE	D<br>D<br>D<br>D	A 326<br>A 389<br>A 392<br>A 393	matches<br>matches<br>matches<br>matches	A 326<br>A 389<br>A 392<br>A 393	11.9 A from NAOAE1K A 607<br>16.2 A from CLA BE1K A 608<br>10.7 A from CLA BE1K A 608<br>15.8 A from C4 PEG A 605	0.06 A	Submit
1h6g<br>PDB<br>PDBsum	ALPHA-1 CATENIN	D<br>D<br>D<br>D	A 326<br>A 389<br>A 392<br>A 393	matches<br>matches<br>matches<br>matches	A 510<br>A 500<br>A 503<br>A 504	4.3 A from CA CA A1632<br>-4.2 A from CA CA A1632s<br>6.0 A from CA CA A1632<br>4.1 A from CA CA A1632	0.66 A	Submit
3a3o<br>PDB<br>PDBsum	TK-SUBTILISIN	D<br>D<br>D<br>D	A 326<br>A 389<br>A 392<br>A 393	matches<br>matches<br>matches<br>matches	A 212<br>A 224<br>A 225<br>A 222	-3.2 A from CA CA A 3s<br>5.4 A from CA CA A 4<br>-3.2 A from CA CA A 3s<br>2.2 A from CA CA A 4s	0.99 A	Submit
3eqz<br>PDB<br>PDBsum	RESPONSE REGULATOR	D<br>D<br>D<br>D	A 326<br>A 389<br>A 392<br>A 393	matches<br>matches<br>matches<br>matches	A 52<br>A 11<br>A 9<br>A 10	none<br>none<br>none<br>none	1.06 A	Submit
5xso<br>PDB<br>PDBsum	RESPONSE REGULATOR FIXJ	D<br>D<br>D<br>D	A 326<br>A 389<br>A 392<br>A 393	matches<br>matches<br>matches<br>matches	A 55<br>A 13<br>A 11<br>A 12	6.2 A from O1 FMT A 405<br>7.1 A from O1 FMT A 407<br>7.5 A from O2 FMT A 405<br>-2.9 A from O1 FMT A 405s	1.08 A	Submit
3ilh<br>PDB<br>PDBsum	TWO COMPONENT RESPONSE REGULATOR	D<br>D<br>D<br>D	A 326<br>A 389<br>A 392<br>A 393	matches<br>matches<br>matches<br>matches	A 65<br>A 16<br>A 14<br>A 15	none<br>none<br>none<br>none	1.13 A	Submit
3nsj<br>PDB<br>PDBsum	PERFORIN-1	D<br>D<br>D<br>D	A 326<br>A 389<br>A 392<br>A 393	matches<br>matches<br>matches<br>matches	A 483<br>A 485<br>A 491<br>A 490	-2.9 A from CA CA A 702s<br>-5.6 A from CA CA A 702s<br>6.1 A from CA CA A 702<br>3.0 A from CA CA A 701s	1.14 A	Submit
4y1s<br>PDB<br>PDBsum	PERFORIN-1	D<br>D<br>D<br>D	A 326<br>A 389<br>A 392<br>A 393	matches<br>matches<br>matches<br>matches	A 435<br>A 485<br>A 483<br>A 490	9.8 A from CA CA A 601<br>-6.3 A from CA CA A 601s<br>9.4 A from CA CA A 601<br>-3.1 A from CA CA A 601s	1.15 A	Submit
1p2f<br>PDB<br>PDBsum	RESPONSE REGULATOR	D<br>D<br>D<br>D	A 326<br>A 389<br>A 392<br>A 393	matches<br>matches<br>matches<br>matches	A 50<br>A 11<br>A 9<br>A 10	none<br>none<br>none<br>none	1.15 A	Submit
5iz5<br>PDB<br>PDBsum	CYTOSOLIC PHOSPHOLIPASE A2 DELTA	D<br>D<br>D<br>D	A 326<br>A 389<br>A 392<br>A 393	matches<br>matches<br>matches<br>matches	A 44<br>A 96<br>A 94<br>A 101	22.1 A from O3 SO4 A 903<br>21.3 A from O3 SO4 A 903<br>19.8 A from O3 SO4 A 903<br>14.6 A from O3 SO4 A 903	1.18 A	Submit
2yeq<br>PDB<br>PDBsum	ALKALINE PHOSPHATASE D	D<br>D<br>D<br>D	A 326<br>A 389<br>A 392<br>A 393	matches<br>matches<br>matches<br>matches	A 210<br>A 380<br>A 209<br>A 151	-2.8 A from CA CA A1526s<br>3.4 A from CA CA A1525s<br>-3.4 A from CA CA A1525s<br>-2.4 A from CA CA A1525s	1.21 A	Submit
4a01	PROTON PYROPHOSPHATASE	D	A 326	matches	A 691	2.7 A from MG MG A1771s	1.21 A	Submit

PDB<br>PDBsum		D<br>D<br>D	A 389<br>A 392<br>A 393	matches<br>matches<br>matches	A 257<br>A 723<br>A 727	-2.5 A from MG MG A1767s<br>3.9 A from MG MG A1769<br>2.5 A from MG MG A1769s		
3pko<br>PDB<br>PDBsum	GERANYLGERANYL PYROPHOSPHATE SYNTHASE	D<br>D<br>D<br>D	A 326<br>A 389<br>A 392<br>A 393	matches<br>matches<br>matches<br>matches	A 234<br>A 215<br>A 219<br>A 216	12.9 A from O3 CIT A 326<br>7.7 A from C4 CIT A 326<br>12.5 A from O3 CIT A 326<br>11.9 A from O4 CIT A 326	1.23 A	Submit
1t8s<br>PDB<br>PDBsum	AMP NUCLEOSIDASE	D<br>D<br>D<br>D	A 326<br>A 389<br>A 392<br>A 393	matches<br>matches<br>matches<br>matches	A 332<br>A 327<br>A 328<br>A 379	16.9 A from C2 FMP A 501<br>11.7 A from C2 FMP A 501<br>11.7 A from O2' FMP A 501<br>10.8 A from C2 FMP A 501	1.23 A	Submit
4rj9<br>PDB<br>PDBsum	C2 DOMAIN-CONTAINING PROTEIN-LIKE	D<br>D<br>D<br>D	A 326<br>A 389<br>A 392<br>A 393	matches<br>matches<br>matches<br>matches	A 28<br>A 76<br>A 74<br>A 80	-3.4 A from K K A 201s<br>4.6 A from K K A 202<br>-2.2 A from K K A 201s<br>7.1 A from K K A 201	1.23 A	Submit
5osw<br>PDB<br>PDBsum	ALBUMIN	D<br>D<br>D<br>D	A 326<br>A 389<br>A 392<br>A 393	matches<br>matches<br>matches<br>matches	A 13<br>A 1<br>A 254<br>A 258	15.5 A from I2 DIU A 601<br>17.4 A from I2 DIU A 601<br>12.7 A from I2 DIU A 601<br>10.1 A from I2 DIU A 601	1.24 A	Submit
3w56<br>PDB<br>PDBsum	C2 DOMAIN PROTEIN	D<br>D<br>D<br>D	A 326<br>A 389<br>A 392<br>A 393	matches<br>matches<br>matches<br>matches	A 41<br>A 90<br>A 88<br>A 94	none<br>none<br>none<br>none	1.24 A	Submit
4jzx<br>PDB<br>PDBsum	FARNESYL PYROPHOSPHATE SYNTHASE	D<br>D<br>D<br>D	A 326<br>A 389<br>A 392<br>A 393	matches<br>matches<br>matches<br>matches	A 268<br>A 250<br>A 254<br>A 251	0.6 A from CG ASP A 268<br>-0.5 A from CG ASP A 250s<br>0.6 A from CG ASP A 254<br>0.6 A from CG ASP A 251	1.24 A	Submit
4lfg<br>PDB<br>PDBsum	GERANYLGERANYL DIPHOSPHATE SYNTHASE	D<br>D<br>D<br>D	A 326<br>A 389<br>A 392<br>A 393	matches<br>matches<br>matches<br>matches	A 232<br>A 213<br>A 217<br>A 214	4.1 A from MG MG A 305<br>-2.6 A from MG MG A 305s<br>4.6 A from MG MG A 305s<br>6.1 A from MG MG A 305	1.24 A	Submit
5ahu<br>PDB<br>PDBsum	FARNESYL PYROPHOSPHATE SYNTHASE, PUTATIVE	D<br>D<br>D<br>D	A 326<br>A 389<br>A 392<br>A 393	matches<br>matches<br>matches<br>matches	B 273<br>B 255<br>B 259<br>B 256	-3.6 A from MG MG B1371s<br>3.0 A from MG MG B1371s<br>-4.2 A from MG MG B1371s<br>5.5 A from MG MG B1371	1.25 A	Submit
3nsj<br>PDB<br>PDBsum	PERFORIN-1	D<br>D<br>D<br>D	A 326<br>A 389<br>A 392<br>A 393	matches<br>matches<br>matches<br>matches	A 435<br>A 485<br>A 483<br>A 490	-3.3 A from CA CA A 702s<br>-5.6 A from CA CA A 702s<br>-2.9 A from CA CA A 702s<br>3.0 A from CA CA A 701s	1.25 A	Submit
4kl0<br>PDB<br>PDBsum	PUTATIVE UNCHARACTERIZED PROTEIN	D<br>D<br>D<br>D	A 326<br>A 389<br>A 392<br>A 393	matches<br>matches<br>matches<br>matches	A 116<br>A 399<br>A 123<br>A 120	-3.1 A from CA CA A 501s<br>-2.9 A from CA CA A 501s<br>-2.4 A from CA CA A 501s<br>4.9 A from CA CA A 501	1.25 A	Submit
5cux<br>PDB<br>PDBsum	ACIDOCALCISOMAL PYROPHOSPHATASE	D<br>D<br>D<br>D	A 326<br>A 389<br>A 392<br>A 393	matches<br>matches<br>matches<br>matches	A 296<br>A 291<br>A 328<br>A 323	6.9 A from O5 POP A 802<br>7.1 A from O5 POP A 802<br>6.4 A from O POP A 802<br>-2.8 A from O3 POP A 802s	1.26 A	Submit
4py9<br>PDB<br>PDBsum	PUTATIVE EXOPOLYPHOSPHATASE-RELATED PROTEIN	D<br>D<br>D<br>D	A 326<br>A 389<br>A 392<br>A 393	matches<br>matches<br>matches<br>matches	A 99<br>A 177<br>A 35<br>A 33	5.0 A from O3 PO4 A 401<br>2.5 A from O3 PO4 A 402s<br>-5.5 A from NA NA A 406s<br>-3.1 A from NA NA A 406s	1.26 A	Submit
6cgj<br>PDB<br>PDBsum	EFFECTOR PROTEIN LEM4 (LPG1101)	D<br>D<br>D<br>D	A 326<br>A 389<br>A 392<br>A 393	matches<br>matches<br>matches<br>matches	A 25<br>A 158<br>A 156<br>A 157	-2.5 A from MG MG A 301s<br>6.6 A from MG MG A 301<br>4.5 A from MG MG A 301<br>-2.5 A from MG MG A 301s	1.26 A	Submit
1rqj<br>PDB<br>PDBsum	GERANYLTRANSTRANSFERASE	D<br>D<br>D<br>D	A 326<br>A 389<br>A 392<br>A 393	matches<br>matches<br>matches<br>matches	A 263<br>A 244<br>A 248<br>A 245	3.9 A from MG MG A 908<br>2.7 A from MG MG A 908s<br>4.7 A from MG MG A 908<br>5.7 A from MG MG A 908	1.27 A	Submit
3x17<br>PDB<br>PDBsum	ENDOGLUCANASE	D<br>D<br>D	A 326<br>A 389<br>A 392	matches<br>matches	A 356<br>A 354<br>A 357	-3.0 A from CA CA A 603s<br>7.3 A from CA CA A 603<br>-2.8 A from CA CA A 603s	1.28 A	Submit

		D	A 393	matches<br>matches	A 351	-2.0 A from CA CA A 603s		
5hn9<br>PDB<br>PDBsum	FARNESYL PYROPHOSPHATE SYNTHASE, PUTATIVE	D<br>D<br>D<br>D	A 326<br>A 389<br>A 392<br>A 393	matches<br>matches<br>matches<br>matches	A 305<br>A 287<br>A 291<br>A 288	10.1 A from CAV 04W A 401<br>4.5 A from OAX 04W A 401<br>9.4 A from OAD 04W A 401<br>9.7 A from CAV 04W A 401	1.28 A	Submit
3jzy<br>PDB<br>PDBsum	INTERSECTIN 2	D<br>D<br>D<br>D	A 326<br>A 389<br>A 392<br>A 393	matches<br>matches<br>matches<br>matches	A1612<br>A1618<br>A1610<br>A1617	6.4 A from UNK UNX A 5<br>3.6 A from UNK UNX A 1<br>8.4 A from UNK UNX A 1<br>7.4 A from UNK UNX A 3	1.29 A	Submit
3aqb<br>PDB<br>PDBsum	COMPONENT A OF HEXAPRENYL DIPHOSPHATE SYNTHASE	D<br>D<br>D<br>D	A 326<br>A 389<br>A 392<br>A 393	matches<br>matches<br>matches<br>matches	B 230<br>B 211<br>B 215<br>B 212	4.0 A from MG MG B 327<br>-3.0 A from MG MG B 327s<br>5.5 A from MG MG B 327<br>5.5 A from MG MG B 327	1.29 A	Submit
6cgj<br>PDB<br>PDBsum	EFFECTOR PROTEIN LEM4 (LPG1101)	D<br>D<br>D<br>D	A 326<br>A 389<br>A 392<br>A 393	matches<br>matches<br>matches<br>matches	A 161<br>A 157<br>A 156<br>A 25	6.1 A from MG MG A 301<br>-2.5 A from MG MG A 301s<br>4.5 A from MG MG A 301<br>-2.5 A from MG MG A 301s	1.30 A	Submit
4y1s<br>PDB<br>PDBsum	PERFORIN-1	D<br>D<br>D<br>D	A 326<br>A 389<br>A 392<br>A 393	matches<br>matches<br>matches<br>matches	A 483<br>A 485<br>A 491<br>A 490	9.4 A from CA CA A 601<br>-6.3 A from CA CA A 601s<br>10.8 A from CA CA A 601<br>-3.1 A from CA CA A 601s	1.30 A	Submit
5m1p<br>PDB<br>PDBsum	TERMINASE LARGE SUBUNIT	D<br>D<br>D<br>D	A 326<br>A 389<br>A 392<br>A 393	matches<br>matches<br>matches<br>matches	A 300<br>A 294<br>A 429<br>A 428	-3.4 A from CA CA A 501s<br>-3.1 A from CA CA A 501s<br>-3.4 A from CA CA A 501s<br>7.2 A from CA CA A 501	1.30 A	Submit
4xlt<br>PDB<br>PDBsum	RESPONSE REGULATOR RECEIVER PROTEIN	D<br>D<br>D<br>D	A 326<br>A 389<br>A 392<br>A 393	matches<br>matches<br>matches<br>matches	A 12<br>A 8<br>A 7<br>A 57	none<br>none<br>none<br>none	1.30 A	Submit
1ux6<br>PDB<br>PDBsum	THROMBOSPONDIN-1	D<br>D<br>D<br>D	A 326<br>A 389<br>A 392<br>A 393	matches<br>matches<br>matches<br>matches	A 881<br>A 877<br>A 879<br>A 887	2.8 A from CA CA A2004s<br>-2.2 A from CA CA A2006s<br>3.1 A from CA CA A2004s<br>-3.1 A from CA CA A2008s	1.30 A	Submit
5c2v<br>PDB<br>PDBsum	HYDRAZINE SYNTHASE ALPHA SUBUNIT	D<br>D<br>D<br>D	A 326<br>A 389<br>A 392<br>A 393	matches<br>matches<br>matches<br>matches	A 384<br>A 404<br>A 403<br>A 409	-4.2 A from CA CA A 903s<br>-6.7 A from CA CA A 903s<br>-2.2 A from CA CA A 903s<br>-3.0 A from CA CA A 903s	1.30 A	Submit
1y9i<br>PDB<br>PDBsum	LOW TEMPERATURE REQUIREMENT C PROTEIN	D<br>D<br>D<br>D	A 326<br>A 389<br>A 392<br>A 393	matches<br>matches<br>matches<br>matches	A 98<br>A 121<br>A 145<br>A 144	2.3 A from CA CA A 501s<br>6.1 A from MG MG A 601<br>-2.6 A from MG MG A 601s<br>-2.7 A from MG MG A 601s	1.30 A	Submit
5ghr<br>PDB<br>PDBsum	SSDNA-SPECIFIC EXONUCLEASE	D<br>D<br>D<br>D	A 326<br>A 389<br>A 392<br>A 393	matches<br>matches<br>matches<br>matches	A 83<br>A 166<br>A 36<br>A 34	8.6 A from O1 SO4 A 501<br>12.7 A from O1 SO4 A 501<br>12.4 A from O1 SO4 A 501<br>10.7 A from O1 SO4 A 501	1.30 A	Submit
4av3<br>PDB<br>PDBsum	K(+)-STIMULATED PYROPHOSPHATE-ENERGIZED SODIUM PUMP	D<br>D<br>D<br>D	A 326<br>A 389<br>A 392<br>A 393	matches<br>matches<br>matches<br>matches	A 660<br>A 202<br>A 692<br>A 696	-3.1 A from CA CA A 728s<br>5.3 A from CA CA A 728<br>-2.8 A from CA CA A 728s<br>6.6 A from CA CA A 728	1.31 A	Submit
2dew<br>PDB<br>PDBsum	PROTEIN-ARGININE DEIMINASE TYPE IV	D<br>D<br>D<br>D	A 326<br>A 389<br>A 392<br>A 393	matches<br>matches<br>matches<br>matches	X 165<br>X 157<br>X 176<br>X 173	3.3 A from CA CA X 901s<br>2.3 A from CA CA X 902s<br>-3.4 A from CA CA X 901s<br>8.2 A from CA CA X 904	1.31 A	Submit
1u02<br>PDB<br>PDBsum	TREHALOSE-6-PHOSPHATE PHOSPHATASE RELATED PROTEIN	D<br>D<br>D<br>D	A 326<br>A 389<br>A 392<br>A 393	matches<br>matches<br>matches<br>matches	A 7<br>A 180<br>A 183<br>A 179	-2.5 A from MG MG A 240s<br>4.4 A from MG MG A 240<br>4.5 A from MG MG A 240<br>-2.5 A from MG MG A 240s	1.31 A	Submit
5afx<br>PDB<br>PDBsum	FARNESYL PYROPHOSPHATE SYNTHASE	D<br>D<br>D<br>D	A 326<br>A 389<br>A 392<br>A 393	matches<br>matches<br>matches<br>matches	A 273<br>A 255<br>A 259<br>A 256	3.6 A from MG MG A1368<br>3.1 A from MG MG A1368s<br>-3.8 A from MG MG A1368s<br>5.7 A from MG MG A1368	1.32 A	Submit

2zxr<br>PDB<br>PDBsum	SINGLE-STRANDED DNA SPECIFIC EXONUCLEASE RECJ	D<br>D<br>D<br>D	A 326<br>A 389<br>A 392<br>A 393	matches<br>matches<br>matches<br>matches	A 136<br>A 221<br>A 84<br>A 82	-2.3 A from MG MG A 667s<br>-3.1 A from MG MG A 667s<br>-3.3 A from MG MG A 667s<br>4.8 A from MG MG A 667	1.33 A	Submit
3apz<br>PDB<br>PDBsum	GERANYL DIPHOSPHATE SYNTHASE	D<br>D<br>D<br>D	A 326<br>A 389<br>A 392<br>A 393	matches<br>matches<br>matches<br>matches	A 253<br>A 234<br>A 238<br>A 235	none<br>none<br>none<br>none	1.33 A	Submit
5iz5<br>PDB<br>PDBsum	CYTOSOLIC PHOSPHOLIPASE A2 DELTA	D<br>D<br>D<br>D	A 326<br>A 389<br>A 392<br>A 393	matches<br>matches<br>matches<br>matches	A 96<br>A 94<br>A 101<br>A 102	21.3 A from O3 SO4 A 903<br>19.8 A from O3 SO4 A 903<br>14.6 A from O3 SO4 A 903<br>18.4 A from O3 SO4 A 903	1.33 A	Submit
3wiu<br>PDB<br>PDBsum	TK-SUBTILISIN	D<br>D<br>D<br>D	A 326<br>A 389<br>A 392<br>A 393	matches<br>matches<br>matches<br>matches	A 216<br>A 224<br>A 214<br>A 225	2.9 A from CA CA A1003s<br>-3.4 A from CA CA A1004s<br>2.9 A from CA CA A1004s<br>-3.3 A from CA CA A1003s	1.34 A	Submit
1ys7<br>PDB<br>PDBsum	TRANSCRIPTIONAL REGULATORY PROTEIN PRRA	D<br>D<br>D<br>D	A 326<br>A 389<br>A 392<br>A 393	matches<br>matches<br>matches<br>matches	A 58<br>A 16<br>A 14<br>A 15	-2.7 A from MG MG A1002s<br>7.1 A from MG MG A1002<br>4.3 A from MG MG A1002<br>-2.5 A from MG MG A1002s	1.34 A	Submit
4exr<br>PDB<br>PDBsum	PUTATIVE LIPOPROTEIN	D<br>D<br>D<br>D	A 326<br>A 389<br>A 392<br>A 393	matches<br>matches<br>matches<br>matches	A 102<br>A 80<br>A 101<br>A 78	17.3 A from NA NA A 301<br>12.1 A from NA NA A 301<br>16.2 A from NA NA A 301<br>18.1 A from NA NA A 301	1.35 A	Submit
6aok<br>PDB<br>PDBsum	CEG4	D<br>D<br>D<br>D	A 326<br>A 389<br>A 392<br>A 393	matches<br>matches<br>matches<br>matches	A 162<br>A 158<br>A 157<br>A 9	5.1 A from CL CL A 303<br>-2.6 A from MG MG A 301s<br>4.7 A from MG MG A 301<br>2.5 A from MG MG A 301s	1.35 A	Submit
4rjw<br>PDB<br>PDBsum	PORIN O	D<br>D<br>D<br>D	A 326<br>A 389<br>A 392<br>A 393	matches<br>matches<br>matches<br>matches	A 177<br>A 213<br>A 175<br>A 212	24.4 A from O15 C8E A 505<br>24.7 A from O15 C8E A 505<br>20.5 A from O15 C8E A 505<br>21.0 A from O15 C8E A 505	1.36 A	Submit
2yvy<br>PDB<br>PDBsum	MG2+ TRANSPORTER MGTE	D<br>D<br>D<br>D	A 326<br>A 389<br>A 392<br>A 393	matches<br>matches<br>matches<br>matches	A 247<br>A 92<br>A 91<br>A 95	-2.7 A from MG MG A 276s<br>4.4 A from MG MG A 276<br>-2.6 A from MG MG A 276s<br>-2.0 A from MG MG A 277s	1.36 A	Submit
1ux6<br>PDB<br>PDBsum	THROMBOSPONDIN-1	D<br>D<br>D<br>D	A 326<br>A 389<br>A 392<br>A 393	matches<br>matches<br>matches<br>matches	A 879<br>A 880<br>A 881<br>A 868	3.1 A from CA CA A2004s<br>-2.2 A from CA CA A2008s<br>2.8 A from CA CA A2004s<br>2.7 A from CA CA A2007s	1.36 A	Submit
3n45<br>PDB<br>PDBsum	FARNESYL PYROPHOSPHATE SYNTHASE	D<br>D<br>D<br>D	A 326<br>A 389<br>A 392<br>A 393	matches<br>matches<br>matches<br>matches	F 261<br>F 243<br>F 247<br>F 244	4.1 A from MG MG F 2<br>2.7 A from MG MG F 2s<br>4.8 A from MG MG F 2<br>5.5 A from O12 ZOL F 354	1.36 A	Submit
4v29<br>PDB<br>PDBsum	AT3G17980	D<br>D<br>D<br>D	A 326<br>A 389<br>A 392<br>A 393	matches<br>matches<br>matches<br>matches	A 39<br>A 87<br>A 85<br>A 92	-3.5 A from CA CA A1179s<br>2.3 A from CA CA A1178s<br>2.4 A from CA CA A1179s<br>7.0 A from CA CA A1179	1.37 A	Submit
5gne<br>PDB<br>PDBsum	LEUCINE AMINOPEPTIDASE	D<br>D<br>D<br>D	A 326<br>A 389<br>A 392<br>A 393	matches<br>matches<br>matches<br>matches	A 288<br>A 225<br>A 227<br>A 226	-1.9 A from ZN ZN A 402s<br>8.2 A from ZN ZN A 402<br>4.8 A from ZN ZN A 402<br>2.8 A from ZN ZN A 402s	1.37 A	Submit
6b04<br>PDB<br>PDBsum	FARNESYL DIPHOSPHATE SYNTHASE	D<br>D<br>D<br>D	A 326<br>A 389<br>A 392<br>A 393	matches<br>matches<br>matches<br>matches	A 305<br>A 287<br>A 291<br>A 288	4.2 A from MG MG A 404<br>2.6 A from MG MG A 404s<br>4.9 A from MG MG A 404<br>5.6 A from MG MG A 404	1.37 A	Submit
1yo8<br>PDB<br>PDBsum	THROMBOSPONDIN-2	D<br>D<br>D<br>D	A 326<br>A 389<br>A 392<br>A 393	matches<br>matches<br>matches<br>matches	A 901<br>A 897<br>A 899<br>A 907	2.8 A from CA CA A1185s<br>-2.1 A from CA CA A1187s<br>3.3 A from CA CA A1185s<br>-2.9 A from CA CA A1189s	1.37 A	Submit
3gin	SODIUM/CALCIUM EXCHANGER	D	A 326	matches	A 499	-2.8 A from CA CA A 2s	1.37 A	Submit

PDB<br>PDBsum	1	D<br>D<br>D	A 389<br>A 392<br>A 393	matches<br>matches<br>matches	A 448<br>A 446<br>A 447	5.8 A from CA CA A 2<br>-3.3 A from CA CA A 2s<br>-3.0 A from CA CA A 7s		
3wiu<br>PDB<br>PDBsum	TK-SUBTILISIN	D<br>D<br>D<br>D	A 326<br>A 389<br>A 392<br>A 393	matches<br>matches<br>matches<br>matches	A 121<br>A 314<br>A 119<br>A 315	16.5 A from CA CA A1001<br>17.6 A from CA CA A1001<br>13.9 A from CA CA A1001<br>12.9 A from CA CA A1001	1.37 A	Submit
1tlq<br>PDB<br>PDBsum	HYPOTHETICAL PROTEIN YPJQ	D<br>D<br>D<br>D	A 326<br>A 389<br>A 392<br>A 393	matches<br>matches<br>matches<br>matches	A 122<br>A 144<br>A 145<br>A 99	5.7 A from CA CA A 190<br>8.3 A from CA CA A 190<br>5.0 A from CA CA A 190<br>-2.3 A from CA CA A 190s	1.37 A	Submit
2c40<br>PDB<br>PDBsum	INOSINE-URIDINE PREFERRING NUCLEOSIDE HYDROLASE FAM	D<br>D<br>D<br>D	A 326<br>A 389<br>A 392<br>A 393	matches<br>matches<br>matches<br>matches	A 247<br>A 38<br>A 14<br>A 9	2.5 A from O3' RIB A1312s<br>-3.8 A from C1' RIB A1312s<br>2.3 A from CA CA A1311s<br>-3.0 A from CA CA A1311s	1.38 A	Submit
3irp<br>PDB<br>PDBsum	URO-ADHERENCE FACTOR A	D<br>D<br>D<br>D	A 326<br>A 389<br>A 392<br>A 393	matches<br>matches<br>matches<br>matches	X 775<br>X 803<br>X 778<br>X 780	3.1 A from K K X 2s<br>5.1 A from K K X 1<br>2.8 A from K K X 2s<br>2.2 A from K K X 1s	1.38 A	Submit
2zxr<br>PDB<br>PDBsum	SINGLE-STRANDED DNA SPECIFIC EXONUCLEASE RECJ	D<br>D<br>D<br>D	A 326<br>A 389<br>A 392<br>A 393	matches<br>matches<br>matches<br>matches	A 221<br>A 82<br>A 84<br>A 136	-3.1 A from MG MG A 667s<br>4.8 A from MG MG A 667<br>-3.3 A from MG MG A 667s<br>-2.3 A from MG MG A 667s	1.38 A	Submit
5wjm<br>PDB<br>PDBsum	CADHERIN-23	D<br>D<br>D<br>D	A 326<br>A 389<br>A 392<br>A 393	matches<br>matches<br>matches<br>matches	A1856<br>A1825<br>A1854<br>A1907	3.3 A from CA CA A2003s<br>7.5 A from CA CA A2004<br>-2.2 A from CA CA A2004s<br>-3.4 A from CA CA A2004s	1.39 A	Submit
3a3o<br>PDB<br>PDBsum	TK-SUBTILISIN	D<br>D<br>D<br>D	A 326<br>A 389<br>A 392<br>A 393	matches<br>matches<br>matches<br>matches	A 224<br>A 212<br>A 222<br>A 225	5.4 A from CA CA A 4<br>-3.2 A from CA CA A 3s<br>2.2 A from CA CA A 4s<br>-3.2 A from CA CA A 3s	1.39 A	Submit
4kl0<br>PDB<br>PDBsum	PUTATIVE UNCHARACTERIZED PROTEIN	D<br>D<br>D<br>D	A 326<br>A 389<br>A 392<br>A 393	matches<br>matches<br>matches<br>matches	A 399<br>A 120<br>A 123<br>A 116	-2.9 A from CA CA A 501s<br>4.9 A from CA CA A 501<br>-2.4 A from CA CA A 501s<br>-3.1 A from CA CA A 501s	1.39 A	Submit
1h6g<br>PDB<br>PDBsum	ALPHA-1 CATENIN	D<br>D<br>D<br>D	A 326<br>A 389<br>A 392<br>A 393	matches<br>matches<br>matches<br>matches	A 500<br>A 510<br>A 504<br>A 503	-4.2 A from CA CA A1632s<br>4.3 A from CA CA A1632<br>4.1 A from CA CA A1632<br>6.0 A from CA CA A1632	1.39 A	Submit
3akb<br>PDB<br>PDBsum	PUTATIVE CALCIUM BINDING PROTEIN	D<br>D<br>D<br>D	A 326<br>A 389<br>A 392<br>A 393	matches<br>matches<br>matches<br>matches	A 26<br>A 18<br>A 29<br>A 20	2.2 A from CA CA A 174s<br>-3.2 A from CA CA A 171s<br>-2.2 A from CA CA A 171s<br>-2.8 A from CA CA A 171s	1.39 A	Submit
4py9<br>PDB<br>PDBsum	PUTATIVE EXOPOLYPHOSPHATASE-RELATED PROTEIN	D<br>D<br>D<br>D	A 326<br>A 389<br>A 392<br>A 393	matches<br>matches<br>matches<br>matches	A 177<br>A 33<br>A 35<br>A 99	2.5 A from O3 PO4 A 402s<br>-3.1 A from NA NA A 406s<br>-5.5 A from NA NA A 406s<br>5.0 A from O3 PO4 A 401	1.39 A	Submit
1u02<br>PDB<br>PDBsum	TREHALOSE-6-PHOSPHATE PHOSPHATASE RELATED PROTEIN	D<br>D<br>D<br>D	A 326<br>A 389<br>A 392<br>A 393	matches<br>matches<br>matches<br>matches	A 180<br>A 9<br>A 179<br>A 7	4.4 A from MG MG A 240<br>-3.6 A from NA NA A 241s<br>-2.5 A from MG MG A 240s<br>-2.5 A from MG MG A 240s	1.40 A	Submit
4rj9<br>PDB<br>PDBsum	C2 DOMAIN-CONTAINING PROTEIN-LIKE	D<br>D<br>D<br>D	A 326<br>A 389<br>A 392<br>A 393	matches<br>matches<br>matches<br>matches	A 76<br>A 81<br>A 74<br>A 80	4.6 A from K K A 202<br>-3.1 A from K K A 202s<br>-2.2 A from K K A 201s<br>7.1 A from K K A 201	1.40 A	Submit
1tlq<br>PDB<br>PDBsum	HYPOTHETICAL PROTEIN YPJQ	D<br>D<br>D<br>D	A 326<br>A 389<br>A 392<br>A 393	matches<br>matches<br>matches<br>matches	A 99<br>A 122<br>A 145<br>A 144	-2.3 A from CA CA A 190s<br>5.7 A from CA CA A 190<br>5.0 A from CA CA A 190<br>8.3 A from CA CA A 190	1.40 A	Submit
5l3d<br>PDB<br>PDBsum	LYSINE-SPECIFIC HISTONE DEMETHYLASE 1A	D<br>D<br>D	A 326<br>A 389<br>A 392	matches<br>matches	A 553<br>A 555<br>A 556	18.6 A from O2 FAD A 901<br>11.2 A from O2 FAD A 901<br>14.6 A from O2 FAD A 901	1.40 A	Submit

		D	A 393	matches<br>matches	A 557	18.9 A from O2 FAD A 901		
5xsp<br>PDB<br>PDBsum	PHOSPHODIESTERASE ACTING ON CYCLIC DINUCLEOTIDES	D<br>D<br>D<br>D	A 326<br>A 389<br>A 392<br>A 393	matches<br>matches<br>matches<br>matches	A 418<br>A 497<br>A 349<br>A 347	2.4 A from MN MN A 701s<br>-2.8 A from MN MN A 701s<br>-3.3 A from MN MN A 701s<br>-2.5 A from MN MN A 702s	1.40 A	Submit
5dkx<br>PDB<br>PDBsum	ALPHA GLUCOSIDASE-LIKE PROTEIN	D<br>D<br>D<br>D	A 326<br>A 389<br>A 392<br>A 393	matches<br>matches<br>matches<br>matches	A 867<br>A 870<br>A 865<br>A 864	25.8 A from C1 TRS A1001<br>29.9 A from CL CL A1003<br>27.3 A from CL CL A1003<br>21.5 A from CL CL A1003	1.40 A	Submit
4i71<br>PDB<br>PDBsum	INOSINE-ADENOSINE-GUANOSINE-NUCLEOSIDE HYDROLASE	D<br>D<br>D<br>D	A 326<br>A 389<br>A 392<br>A 393	matches<br>matches<br>matches<br>matches	A 261<br>A 40<br>A 15<br>A 10	2.5 A from O3' AGV A 401s<br>-2.9 A from N3 AGV A 401s<br>2.2 A from CA CA A 402s<br>-3.0 A from CA CA A 402s	1.41 A	Submit
1yo8<br>PDB<br>PDBsum	THROMBOSPONDIN-2	D<br>D<br>D<br>D	A 326<br>A 389<br>A 392<br>A 393	matches<br>matches<br>matches<br>matches	A 743<br>A 744<br>A 745<br>A 732	3.2 A from CA CA A1205s<br>-2.4 A from CA CA A1208s<br>2.6 A from CA CA A1205s<br>2.6 A from CA CA A1206s	1.41 A	Submit
3gin<br>PDB<br>PDBsum	SODIUM/CALCIUM EXCHANGER 1	D<br>D<br>D<br>D	A 326<br>A 389<br>A 392<br>A 393	matches<br>matches<br>matches<br>matches	A 447<br>A 499<br>A 500<br>A 498	-3.0 A from CA CA A 7s<br>-2.8 A from CA CA A 2s<br>2.4 A from CA CA A 2s<br>-2.3 A from CA CA A 1s	1.41 A	Submit
5ghr<br>PDB<br>PDBsum	SSDNA-SPECIFIC EXONUCLEASE	D<br>D<br>D<br>D	A 326<br>A 389<br>A 392<br>A 393	matches<br>matches<br>matches<br>matches	A 166<br>A 34<br>A 36<br>A 83	12.7 A from O1 SO4 A 501<br>10.7 A from O1 SO4 A 501<br>12.4 A from O1 SO4 A 501<br>8.6 A from O1 SO4 A 501	1.41 A	Submit
3mfi<br>PDB<br>PDBsum	DNA POLYMERASE ETA	D<br>D<br>D<br>D	A 326<br>A 389<br>A 392<br>A 393	matches<br>matches<br>matches<br>matches	A 289<br>A 232<br>A 234<br>A 235	18.7 A from O2 SO4 A 517<br>13.7 A from O2 SO4 A 517<br>17.2 A from O2 SO4 A 517<br>15.9 A from O1 SO4 A 517	1.42 A	Submit
5cvw<br>PDB<br>PDBsum	BIFUNCTIONAL HEMOLYSIN/ADENYLATE CYCLASE	D<br>D<br>D<br>D	A 326<br>A 389<br>A 392<br>A 393	matches<br>matches<br>matches<br>matches	A1599<br>A1560<br>A1579<br>A1580	-2.1 A from CA CA A1703s<br>5.2 A from MG MG A1715<br>2.4 A from CA CA A1702s<br>4.5 A from MG MG A1715	1.42 A	Submit
3nsj<br>PDB<br>PDBsum	PERFORIN-1	D<br>D<br>D<br>D	A 326<br>A 389<br>A 392<br>A 393	matches<br>matches<br>matches<br>matches	A 485<br>A 483<br>A 490<br>A 491	-5.6 A from CA CA A 702s<br>-2.9 A from CA CA A 702s<br>3.0 A from CA CA A 701s<br>6.1 A from CA CA A 702	1.42 A	Submit
2f3o<br>PDB<br>PDBsum	PYRUVATE FORMATE-LYASE 2	D<br>D<br>D<br>D	A 326<br>A 389<br>A 392<br>A 393	matches<br>matches<br>matches<br>matches	A 597<br>A 595<br>A 600<br>A 596	31.1 A from O1 GOL A 778<br>29.5 A from O1 GOL A 778<br>27.0 A from O1 GOL A 778<br>32.6 A from O1 GOL A 778	1.42 A	Submit
1wpn<br>PDB<br>PDBsum	MANGANESE-DEPENDENT INORGANIC PYROPHOSPHATASE	D<br>D<br>D<br>D	A 326<br>A 389<br>A 392<br>A 393	matches<br>matches<br>matches<br>matches	A 75<br>A 149<br>A 15<br>A 13	2.6 A from MN MN A 601s<br>-2.6 A from MN MN A 602s<br>-3.1 A from MN MN A 602s<br>-2.1 A from MN MN A 601s	1.42 A	Submit
4rj9<br>PDB<br>PDBsum	C2 DOMAIN-CONTAINING PROTEIN-LIKE	D<br>D<br>D<br>D	A 326<br>A 389<br>A 392<br>A 393	matches<br>matches<br>matches<br>matches	A 23<br>A 81<br>A 74<br>A 76	4.5 A from K K A 201<br>-3.1 A from K K A 202s<br>-2.2 A from K K A 201s<br>4.6 A from K K A 202	1.42 A	Submit
1ulv<br>PDB<br>PDBsum	GLUCODEXTRANASE	D<br>D<br>D<br>D	A 326<br>A 389<br>A 392<br>A 393	matches<br>matches<br>matches<br>matches	A 782<br>A 807<br>A 786<br>A 990	2.4 A from CA CA A2006s<br>-3.3 A from CA CA A2005s<br>3.0 A from CA CA A2006s<br>-3.9 A from CA CA A2005s	1.42 A	Submit
3vay<br>PDB<br>PDBsum	HAD-SUPERFAMILY HYDROLASE	D<br>D<br>D<br>D	A 326<br>A 389<br>A 392<br>A 393	matches<br>matches<br>matches<br>matches	A 184<br>A 180<br>A 185<br>A 8	7.4 A from MG MG A 301<br>-2.8 A from MG MG A 301s<br>4.7 A from MG MG A 301<br>-2.4 A from MG MG A 301s	1.43 A	Submit
3pdd<br>PDB<br>PDBsum	GLYCOSIDE HYDROLASE, FAMILY 9	D<br>D<br>D<br>D	A 326<br>A 389<br>A 392<br>A 393	matches<br>matches<br>matches<br>matches	A 121<br>A 163<br>A 119<br>A 164	-2.9 A from CA CA A 193s<br>-3.2 A from CA CA A 193s<br>-2.1 A from CA CA A 193s<br>5.4 A from CA CA A 193	1.43 A	Submit

2wtf<br>PDB<br>PDBsum	DNA POLYMERASE ETA	D<br>D<br>D<br>D	A 326 matches A 289<br>A 389 matches A 232<br>A 392 matches A 234<br>A 393 matches A 235	-2.7 A from CA CA A1514s<br>5.7 A from CA CA A1514<br>4.0 A from CA CA A1514<br>5.1 A from CA CA A1514	1.43 A	Submit
1ulv<br>PDB<br>PDBsum	GLUCODEXTRANASE	D<br>D<br>D<br>D	A 326 matches A 782<br>A 389 matches A 786<br>A 392 matches A 990<br>A 393 matches A 807	2.4 A from CA CA A2006s<br>3.0 A from CA CA A2006s<br>-3.9 A from CA CA A2005s<br>-3.3 A from CA CA A2005s	1.43 A	Submit
5t5i<br>PDB<br>PDBsum	TUNGSTEN FORMYLMETHANOFURAN DEHYDROGENASE SUBUNIT F	D<br>D<br>D<br>D	A 326 matches A 136<br>A 389 matches A 499<br>A 392 matches A 466<br>A 393 matches A 464	16.0 A from O2 GOL A 609<br>15.1 A from O2 GOL A 609<br>16.4 A from O2 GOL A 609<br>20.7 A from O2 GOL A 609	1.43 A	Submit
2eb0<br>PDB<br>PDBsum	MANGANESE-DEPENDENT INORGANIC PYROPHOSPHATASE	D<br>D<br>D<br>D	A 326 matches A 66<br>A 389 matches A 147<br>A 392 matches A 13<br>A 393 matches A 11	2.6 A from MN MN A 502s<br>-2.6 A from MN MN A 502s<br>-3.2 A from MN MN A 502s<br>-2.5 A from MN MN A 501s	1.44 A	Submit
1yo8<br>PDB<br>PDBsum	THROMBOSPONDIN-2	D<br>D<br>D<br>D	A 326 matches A 745<br>A 389 matches A 741<br>A 392 matches A 743<br>A 393 matches A 751	2.6 A from CA CA A1205s<br>-2.4 A from CA CA A1207s<br>3.2 A from CA CA A1205s<br>-3.2 A from CA CA A1208s	1.44 A	Submit
3epw<br>PDB<br>PDBsum	IAG-NUCLEOSIDE HYDROLASE	D<br>D<br>D<br>D	A 326 matches A 261<br>A 389 matches A 40<br>A 392 matches A 15<br>A 393 matches A 10	2.6 A from O3' JMQ A1002s<br>-3.0 A from N3 JMQ A1002s<br>2.2 A from CA CA A1001s<br>-3.0 A from CA CA A1001s	1.44 A	Submit
3tc1<br>PDB<br>PDBsum	OCTAPRENYL PYROPHOSPHATE SYNTHASE	D<br>D<br>D<br>D	A 326 matches A 219<br>A 389 matches A 200<br>A 392 matches A 204<br>A 393 matches A 201	4.0 A from MG MG A 500<br>-2.9 A from MG MG A 500s<br>5.6 A from MG MG A 500<br>5.5 A from MG MG A 500	1.44 A	Submit
1yo8<br>PDB<br>PDBsum	THROMBOSPONDIN-2	D<br>D<br>D<br>D	A 326 matches A 899<br>A 389 matches A 900<br>A 392 matches A 901<br>A 393 matches A 888	3.3 A from CA CA A1185s<br>-2.4 A from CA CA A1189s<br>2.8 A from CA CA A1185s<br>2.6 A from CA CA A1188s	1.44 A	Submit
5f56<br>PDB<br>PDBsum	SINGLE-STRANDED-DNA-SPECIFIC EXONUCLEASE	D<br>D<br>D<br>D	A 326 matches A 135<br>A 389 matches A 223<br>A 392 matches A 83<br>A 393 matches A 81	2.6 A from MN MN A 805s<br>-2.6 A from MN MN A 805s<br>-3.3 A from MN MN A 805s<br>-1.9 A from MN MN A 804s	1.44 A	Submit
5m1p<br>PDB<br>PDBsum	TERMINASE LARGE SUBUNIT	D<br>D<br>D<br>D	A 326 matches A 428<br>A 389 matches A 300<br>A 392 matches A 429<br>A 393 matches A 294	7.2 A from CA CA A 501<br>-3.4 A from CA CA A 501s<br>-3.4 A from CA CA A 501s<br>-3.1 A from CA CA A 501s	1.45 A	Submit
5wtl<br>PDB<br>PDBsum	OMPA FAMILY PROTEIN	D<br>D<br>D<br>D	A 326 matches A 281<br>A 389 matches A 279<br>A 392 matches A 290<br>A 393 matches A 287	2.8 A from CA CA A1008s<br>-3.3 A from CA CA A1007s<br>2.1 A from CA CA A1008s<br>5.4 A from CA CA A1007	1.45 A	Submit
4aqo<br>PDB<br>PDBsum	COLLAGENASE	D<br>D<br>D	A 326 matches A 825<br>A 389 matches A 864<br>A 392 matches A 823	-2.9 A from CA CA A1881s<br>-3.3 A from CA CA A1881s<br>-2.1 A from CA CA A1881s	1.45 A	Submit

Results of ASSAM search and lefthanded superposition for 1ea5_4D
➔ Download text version of the ASSAM output

Matches found in 1ea5_4D (PDB ID)	Description	Residues	Residue Matches<br>Query Database Hits	Heteroatoms Notes in Database hit	RMSD	
3jzy<br>PDB<br>PDBsum	INTERSECTIN 2	D<br>D<br>D	A 326 matches A1612<br>A 389 matches A1617<br>A 392 matches A1610<br>matches	6.4 A from UNK UNX A 5<br>7.4 A from UNK UNX A 3<br>8.4 A from UNK UNX A 1	1.02 A	Submit

		D	A 393		A1618	3.6 A from UNK UNX A 1		
5iz5<br>PDB<br>PDBsum	CYTOSOLIC PHOSPHOLIPASE A2 DELTA	D<br>D<br>D<br>D	A 326<br>A 389<br>A 392<br>A 393	matches<br>matches<br>matches<br>matches	A 96<br>A 102<br>A 101<br>A 94	21.3 A from O3 SO4 A 903<br>18.4 A from O3 SO4 A 903<br>14.6 A from O3 SO4 A 903<br>19.8 A from O3 SO4 A 903	1.13 A	Submit
6g1u<br>PDB<br>PDBsum	ACETYLCHOLINESTERASE	D<br>D<br>D<br>D	A 326<br>A 389<br>A 392<br>A 393	matches<br>matches<br>matches<br>matches	A 326<br>A 393<br>A 392<br>A 389	11.9 A from NAOAE1K A 607<br>15.8 A from C4 PEG A 605<br>10.7 A from CLA BE1K A 608<br>16.2 A from CLA BE1K A 608	1.17 A	Submit
4iik<br>PDB<br>PDBsum	ADENOSINE MONOPHOSPHATE-PROTEIN HYDROLASE SIDD	D<br>D<br>D<br>D	A 326<br>A 389<br>A 392<br>A 393	matches<br>matches<br>matches<br>matches	A 327<br>A 91<br>A 92<br>A 110	5.5 A from MG MG A 401<br>5.0 A from MG MG A 401<br>5.3 A from MG MG A 401<br>2.8 A from MG MG A 401s	1.18 A	Submit
2zxr<br>PDB<br>PDBsum	SINGLE-STRANDED DNA SPECIFIC EXONUCLEASE RECJ	D<br>D<br>D<br>D	A 326<br>A 389<br>A 392<br>A 393	matches<br>matches<br>matches<br>matches	A 221<br>A 136<br>A 84<br>A 82	-3.1 A from MG MG A 667s<br>-2.3 A from MG MG A 667s<br>-3.3 A from MG MG A 667s<br>4.8 A from MG MG A 667	1.18 A	Submit
4c7i<br>PDB<br>PDBsum	GLYCYLPEPTIDE N-TETRADECANOYLTRANSFERASE	D<br>D<br>D<br>D	A 326<br>A 389<br>A 392<br>A 393	matches<br>matches<br>matches<br>matches	A 396<br>A 85<br>A 84<br>A 83	-3.4 A from N JJ1 A1424s<br>-3.7 A from N JJ1 A1424s<br>-4.7 A from O3 JJ1 A1424s<br>-3.9 A from N JJ1 A1424s	1.22 A	Submit
4wk0<br>PDB<br>PDBsum	INTEGRIN ALPHA-5	D<br>D<br>D<br>D	A 326<br>A 389<br>A 392<br>A 393	matches<br>matches<br>matches<br>matches	A 297<br>A 300<br>A 293<br>A 301	-2.9 A from CA CA A 502s<br>7.5 A from CA CA A 502<br>-3.3 A from CA CA A 502s<br>-2.3 A from CA CA A 502s	1.23 A	Submit
4v29<br>PDB<br>PDBsum	AT3G17980	D<br>D<br>D<br>D	A 326<br>A 389<br>A 392<br>A 393	matches<br>matches<br>matches<br>matches	A 34<br>A 92<br>A 85<br>A 93	2.3 A from CA CA A1179s<br>7.0 A from CA CA A1179<br>2.4 A from CA CA A1179s<br>4.5 A from CA CA A1178	1.24 A	Submit
5m1p<br>PDB<br>PDBsum	TERMINASE LARGE SUBUNIT	D<br>D<br>D<br>D	A 326<br>A 389<br>A 392<br>A 393	matches<br>matches<br>matches<br>matches	A 300<br>A 428<br>A 429<br>A 294	-3.4 A from CA CA A 501s<br>7.2 A from CA CA A 501<br>-3.4 A from CA CA A 501s<br>-3.1 A from CA CA A 501s	1.25 A	Submit
5olr<br>PDB<br>PDBsum	RHAMNOGALACTURONAN LYASE	D<br>D<br>D<br>D	A 326<br>A 389<br>A 392<br>A 393	matches<br>matches<br>matches<br>matches	A 280<br>A 250<br>A 247<br>A 215	3.1 A from CA CA A 602s<br>2.8 A from O3 PO4 A 601s<br>3.4 A from CA CA A 603s<br>-2.2 A from CA CA A 603s	1.25 A	Submit
6byc<br>PDB<br>PDBsum	BETA-MANNOSIDASE	D<br>D<br>D<br>D	A 326<br>A 389<br>A 392<br>A 393	matches<br>matches<br>matches<br>matches	A 235<br>A 335<br>A 333<br>A 331	20.7 A from O1 GOL A 902<br>24.8 A from O1 GOL A 902<br>24.8 A from O1 GOL A 902<br>22.8 A from O1 GOL A 902	1.27 A	Submit
4iik<br>PDB<br>PDBsum	ADENOSINE MONOPHOSPHATE-PROTEIN HYDROLASE SIDD	D<br>D<br>D<br>D	A 326<br>A 389<br>A 392<br>A 393	matches<br>matches<br>matches<br>matches	A 326<br>A 91<br>A 327<br>A 92	-3.6 A from MG MG A 402s<br>5.0 A from MG MG A 401<br>5.5 A from MG MG A 401<br>5.3 A from MG MG A 401	1.27 A	Submit
5ghr<br>PDB<br>PDBsum	SSDNA-SPECIFIC EXONUCLEASE	D<br>D<br>D<br>D	A 326<br>A 389<br>A 392<br>A 393	matches<br>matches<br>matches<br>matches	A 166<br>A 83<br>A 36<br>A 34	12.7 A from O1 SO4 A 501<br>8.6 A from O1 SO4 A 501<br>12.4 A from O1 SO4 A 501<br>10.7 A from O1 SO4 A 501	1.27 A	Submit
4py9<br>PDB<br>PDBsum	PUTATIVE EXOPOLYPHOSPHATASE-RELATED PROTEIN	D<br>D<br>D<br>D	A 326<br>A 389<br>A 392<br>A 393	matches<br>matches<br>matches<br>matches	A 177<br>A 99<br>A 35<br>A 33	2.5 A from O3 PO4 A 402s<br>5.0 A from O3 PO4 A 401<br>-5.5 A from NA NA A 406s<br>-3.1 A from NA NA A 406s	1.28 A	Submit
3kwu<br>PDB<br>PDBsum	MUNC13-1	D<br>D<br>D<br>D	A 326<br>A 389<br>A 392<br>A 393	matches<br>matches<br>matches<br>matches	A 705<br>A 775<br>A 757<br>A 776	2.2 A from CA CA A 901s<br>-3.7 A from O3 AGOL A 921s<br>2.7 A from CA CA A 901s<br>-3.3 A from CA CA A 902s	1.29 A	Submit
4xlt<br>PDB<br>PDBsum	RESPONSE REGULATOR RECEIVER PROTEIN	D<br>D<br>D<br>D	A 326<br>A 389<br>A 392<br>A 393	matches<br>matches<br>matches<br>matches	A 12<br>A 57<br>A 7<br>A 8	none<br>none<br>none<br>none	1.30 A	Submit

5cux PDB PDBsum	ACIDOCALCISOMAL PYROPHOSPHATASE	D D D D	A 326 matches A 296<br>A 389 matches A 323<br>A 392 matches A 328<br>A 393 matches A 291	6.9 A from O5 POP A 802<br>-2.8 A from O3 POP A 802s<br>6.4 A from O POP A 802<br>7.1 A from O5 POP A 802	1.30 A	Submit
4pkf PDB PDBsum	TUTD	D D D D	A 326 matches A 671<br>A 389 matches A 673<br>A 392 matches A 676<br>A 393 matches A 672	28.4 A from CL CL A 902<br>31.8 A from CL CL A 902<br>25.0 A from CL CL A 902<br>31.1 A from CL CL A 902	1.30 A	Submit
3psf PDB PDBsum	TRANSCRIPTION ELONGATION FACTOR SPT6	D D D D	A 326 matches A 358<br>A 389 matches A 412<br>A 392 matches A 413<br>A 393 matches A 416	none<br>none<br>none<br>none	1.30 A	Submit
2yvy PDB PDBsum	MG2+ TRANSPORTER MGTE	D D D D	A 326 matches A 247<br>A 389 matches A 95<br>A 392 matches A 91<br>A 393 matches A 92	-2.7 A from MG MG A 276s<br>-2.0 A from MG MG A 277s<br>-2.6 A from MG MG A 276s<br>4.4 A from MG MG A 276	1.31 A	Submit
1tlq PDB PDBsum	HYPOTHETICAL PROTEIN YPJQ	D D D D	A 326 matches A 99<br>A 389 matches A 144<br>A 392 matches A 145<br>A 393 matches A 122	-2.3 A from CA CA A 190s<br>8.3 A from CA CA A 190<br>5.0 A from CA CA A 190<br>5.7 A from CA CA A 190	1.32 A	Submit
5cvw PDB PDBsum	BIFUNCTIONAL HEMOLYSIN/ADENYLATE CYCLASE	D D D D	A 326 matches A1560<br>A 389 matches A1599<br>A 392 matches A1579<br>A 393 matches A1580	5.2 A from MG MG A1715<br>-2.1 A from CA CA A1703s<br>2.4 A from CA CA A1702s<br>4.5 A from MG MG A1715	1.32 A	Submit
4rj9 PDB PDBsum	C2 DOMAIN-CONTAINING PROTEIN-LIKE	D D D D	A 326 matches A 23<br>A 389 matches A 76<br>A 392 matches A 74<br>A 393 matches A 81	4.5 A from K K A 201<br>4.6 A from K K A 202<br>-2.2 A from K K A 201s<br>-3.1 A from K K A 202s	1.32 A	Submit
5ovo PDB PDBsum	ADP-RIBOSYL-(DINITROGEN REDUCTASE) HYDROLASE	D D D D	A 326 matches A 60<br>A 389 matches A 245<br>A 392 matches A 61<br>A 393 matches A 21	3.7 A from MG MG A 302<br>-2.6 A from MG MG A 302s<br>4.1 A from MG MG A 302<br>7.3 A from MG MG A 302	1.32 A	Submit
5l3d PDB PDBsum	LYSINE-SPECIFIC HISTONE DEMETHYLASE 1A	D D D D	A 326 matches A 553<br>A 389 matches A 557<br>A 392 matches A 556<br>A 393 matches A 555	18.6 A from O2 FAD A 901<br>18.9 A from O2 FAD A 901<br>14.6 A from O2 FAD A 901<br>11.2 A from O2 FAD A 901	1.32 A	Submit
2di4 PDB PDBsum	CELL DIVISION PROTEIN FTSH HOMOLOG	D D D D	A 326 matches A 465<br>A 389 matches A 433<br>A 392 matches A 431<br>A 393 matches A 432	20.6 A from HG HG A1001<br>18.2 A from HG HG A1001<br>20.3 A from HG HG A1001<br>13.0 A from HG HG A1001	1.33 A	Submit
3x17 PDB PDBsum	ENDOGLUCANASE	D D D D	A 326 matches A 351<br>A 389 matches A 354<br>A 392 matches A 357<br>A 393 matches A 356	-2.0 A from CA CA A 603s<br>7.3 A from CA CA A 603<br>-2.8 A from CA CA A 603s<br>-3.0 A from CA CA A 603s	1.33 A	Submit
3mvs PDB PDBsum	CADHERIN-23	D D D D	A 326 matches A 38<br>A 389 matches A 86<br>A 392 matches A 40<br>A 393 matches A 36	0.6 A from CG ASP A 38s<br>-0.5 A from CG ASP A 86s<br>0.5 A from CG ASP A 40s<br>-0.6 A from CG ASP A 36s	1.34 A	Submit
3gin PDB PDBsum	SODIUM/CALCIUM EXCHANGER 1	D D D D	A 326 matches A 500<br>A 389 matches A 446<br>A 392 matches A 448<br>A 393 matches A 447	2.4 A from CA CA A 2s<br>-3.3 A from CA CA A 2s<br>5.8 A from CA CA A 2<br>-3.0 A from CA CA A 7s	1.34 A	Submit
2f3o PDB PDBsum	PYRUVATE FORMATE-LYASE 2	D D D D	A 326 matches A 597<br>A 389 matches A 595<br>A 392 matches A 596<br>A 393 matches A 600	31.1 A from O1 GOL A 778<br>29.5 A from O1 GOL A 778<br>32.6 A from O1 GOL A 778<br>27.0 A from O1 GOL A 778	1.35 A	Submit
2yn5 PDB PDBsum	PUTATIVE INNER MEMBRANE PROTEIN	D D D D	A 326 matches A5256<br>A 389 matches A5294<br>A 392 matches A5343<br>A 393 matches A5296	-2.2 A from CA CA A6364s<br>6.0 A from CA CA A6364<br>-3.3 A from CA CA A6364s<br>-3.0 A from CA CA A6364s	1.35 A	Submit
1tlq	HYPOTHETICAL PROTEIN YPJQ	D	A 326 matches A 122	5.7 A from CA CA A 190	1.35 A	Submit

PDB<br>PDBsum		D<br>D<br>D	A 389<br>A 392<br>A 393	matches<br>matches<br>matches	A 99<br>A 145<br>A 144	-2.3 A from CA CA A 190s<br>5.0 A from CA CA A 190<br>8.3 A from CA CA A 190		
4kl0<br>PDB<br>PDBsum	PUTATIVE UNCHARACTERIZED PROTEIN	D<br>D<br>D<br>D	A 326<br>A 389<br>A 392<br>A 393	matches<br>matches<br>matches<br>matches	A 399<br>A 116<br>A 123<br>A 120	-2.9 A from CA CA A 501s<br>-3.1 A from CA CA A 501s<br>-2.4 A from CA CA A 501s<br>4.9 A from CA CA A 501	1.35 A	Submit
3pdd<br>PDB<br>PDBsum	GLYCOSIDE HYDROLASE, FAMILY 9	D<br>D<br>D<br>D	A 326<br>A 389<br>A 392<br>A 393	matches<br>matches<br>matches<br>matches	A 165<br>A 119<br>A 163<br>A 164	5.1 A from CA CA A 193<br>-2.1 A from CA CA A 193s<br>-3.2 A from CA CA A 193s<br>5.4 A from CA CA A 193	1.35 A	Submit
3hfw<br>PDB<br>PDBsum	PROTEIN ADP-RIBOSYLARGININE HYDROLASE	D<br>D<br>D<br>D	A 326<br>A 389<br>A 392<br>A 393	matches<br>matches<br>matches<br>matches	A 55<br>A 304<br>A 56<br>A 15	-2.9 A from MG MG A 361s<br>-2.7 A from MG MG A 361s<br>-2.9 A from MG MG A 361s<br>4.6 A from MG MG A 361	1.35 A	Submit
2wtf<br>PDB<br>PDBsum	DNA POLYMERASE ETA	D<br>D<br>D<br>D	A 326<br>A 389<br>A 392<br>A 393	matches<br>matches<br>matches<br>matches	A 289<br>A 232<br>A 235<br>A 234	-2.7 A from CA CA A1514s<br>5.7 A from CA CA A1514<br>5.1 A from CA CA A1514<br>4.0 A from CA CA A1514	1.36 A	Submit
4kl0<br>PDB<br>PDBsum	PUTATIVE UNCHARACTERIZED PROTEIN	D<br>D<br>D<br>D	A 326<br>A 389<br>A 392<br>A 393	matches<br>matches<br>matches<br>matches	A 116<br>A 120<br>A 123<br>A 399	-3.1 A from CA CA A 501s<br>4.9 A from CA CA A 501<br>-2.4 A from CA CA A 501s<br>-2.9 A from CA CA A 501s	1.36 A	Submit
2yzv<br>PDB<br>PDBsum	ADP-RIBOSYLGLYCOHYDROLASE	D<br>D<br>D<br>D	A 326<br>A 389<br>A 392<br>A 393	matches<br>matches<br>matches<br>matches	A 57<br>A 253<br>A 58<br>A 22	-3.2 A from MG MG A 502s<br>2.3 A from MG MG A 501s<br>-2.8 A from MG MG A 502s<br>4.3 A from MG MG A 502	1.36 A	Submit
5xsp<br>PDB<br>PDBsum	PHOSPHODIESTERASE ACTING ON CYCLIC DINUCLEOTIDES	D<br>D<br>D<br>D	A 326<br>A 389<br>A 392<br>A 393	matches<br>matches<br>matches<br>matches	A 497<br>A 418<br>A 349<br>A 347	-2.8 A from MN MN A 701s<br>2.4 A from MN MN A 701s<br>-3.3 A from MN MN A 701s<br>-2.5 A from MN MN A 702s	1.36 A	Submit
2r25<br>PDB<br>PDBsum	PHOSPHORELAY INTERMEDIATE PROTEIN YPD1	D<br>D<br>D<br>D	A 326<br>A 389<br>A 392<br>A 393	matches<br>matches<br>matches<br>matches	A 26<br>A 21<br>A 24<br>A 22	24.8 A from MG MG B 1<br>23.0 A from F3 BEF B 2<br>28.0 A from F3 BEF B 2<br>26.5 A from F3 BEF B 2	1.36 A	Submit
3akb<br>PDB<br>PDBsum	PUTATIVE CALCIUM BINDING PROTEIN	D<br>D<br>D<br>D	A 326<br>A 389<br>A 392<br>A 393	matches<br>matches<br>matches<br>matches	A 18<br>A 26<br>A 29<br>A 20	-3.2 A from CA CA A 171s<br>2.2 A from CA CA A 174s<br>-2.2 A from CA CA A 171s<br>-2.8 A from CA CA A 171s	1.36 A	Submit
5t5i<br>PDB<br>PDBsum	TUNGSTEN FORMYLMETHANOFURAN DEHYDROGENASE SUBUNIT F	D<br>D<br>D<br>D	A 326<br>A 389<br>A 392<br>A 393	matches<br>matches<br>matches<br>matches	A 136<br>A 464<br>A 466<br>A 499	16.0 A from O2 GOL A 609<br>20.7 A from O2 GOL A 609<br>16.4 A from O2 GOL A 609<br>15.1 A from O2 GOL A 609	1.37 A	Submit
3wiu<br>PDB<br>PDBsum	TK-SUBTILISIN	D<br>D<br>D<br>D	A 326<br>A 389<br>A 392<br>A 393	matches<br>matches<br>matches<br>matches	A 216<br>A 224<br>A 222<br>A 225	2.9 A from CA CA A1003s<br>-3.4 A from CA CA A1004s<br>2.3 A from CA CA A1004s<br>-3.3 A from CA CA A1003s	1.37 A	Submit
3mfi<br>PDB<br>PDBsum	DNA POLYMERASE ETA	D<br>D<br>D<br>D	A 326<br>A 389<br>A 392<br>A 393	matches<br>matches<br>matches<br>matches	A 289<br>A 232<br>A 235<br>A 234	18.7 A from O2 SO4 A 517<br>13.7 A from O2 SO4 A 517<br>15.9 A from O1 SO4 A 517<br>17.2 A from O2 SO4 A 517	1.37 A	Submit
5o6h<br>PDB<br>PDBsum	GLYCYLPEPTIDE N-TETRADECANOYLTRANSFERASE 1	D<br>D<br>D<br>D	A 326<br>A 389<br>A 392<br>A 393	matches<br>matches<br>matches<br>matches	A 471<br>A 185<br>A 184<br>A 183	6.6 A from C17 9M2 A2005<br>10.3 A from C17 9M2 A2005<br>7.0 A from C20 9M2 A2005<br>5.9 A from N3 9M2 A2005	1.37 A	Submit
1y9i<br>PDB<br>PDBsum	LOW TEMPERATURE REQUIREMENT C PROTEIN	D<br>D<br>D<br>D	A 326<br>A 389<br>A 392<br>A 393	matches<br>matches<br>matches<br>matches	A 98<br>A 144<br>A 145<br>A 121	2.3 A from CA CA A 501s<br>-2.7 A from MG MG A 601s<br>-2.6 A from MG MG A 601s<br>6.1 A from MG MG A 601	1.37 A	Submit
3mi6<br>PDB<br>PDBsum	ALPHA-GALACTOSIDASE	D<br>D<br>D	A 326<br>A 389<br>A 392	matches<br>matches	A 130<br>A 128<br>A 129	none<br>none<br>none	1.38 A	Submit

		D	A 393	matches<br>matches	A 125	none		
4exr<br>PDB<br>PDBsum	PUTATIVE LIPOPROTEIN	D<br>D<br>D<br>D	A 326<br>A 389<br>A 392<br>A 393	matches<br>matches<br>matches<br>matches	A 102<br>A 78<br>A 101<br>A 80	17.3 A from NA NA A 301<br>18.1 A from NA NA A 301<br>16.2 A from NA NA A 301<br>12.1 A from NA NA A 301	1.38 A	Submit
6c33<br>PDB<br>PDBsum	5'-3' EXONUCLEASE	D<br>D<br>D<br>D	A 326<br>A 389<br>A 392<br>A 393	matches<br>matches<br>matches<br>matches	A 208<br>A 125<br>A 148<br>A 146	-2.9 A from MN MN A 403s<br>2.7 A from MN MN A 401s<br>1.9 A from MN MN A 403s<br>-2.7 A from MN MN A 402s	1.38 A	Submit
4pkf<br>PDB<br>PDBsum	TUTD	D<br>D<br>D<br>D	A 326<br>A 389<br>A 392<br>A 393	matches<br>matches<br>matches<br>matches	A 673<br>A 671<br>A 672<br>A 676	31.8 A from CL CL A 902<br>28.4 A from CL CL A 902<br>31.1 A from CL CL A 902<br>25.0 A from CL CL A 902	1.38 A	Submit
2gfh<br>PDB<br>PDBsum	HALOACID DEHALOGENASE-LIKE HYDROLASE DOMAIN CONTAIN	D<br>D<br>D<br>D	A 326<br>A 389<br>A 392<br>A 393	matches<br>matches<br>matches<br>matches	A 194<br>A 14<br>A 12<br>A 189	4.6 A from O3 PO4 A 251<br>3.6 A from O2 PO4 A 251s<br>3.4 A from O3 PO4 A 251s<br>-2.8 A from NA NA A 249s	1.39 A	Submit
1ir6<br>PDB<br>PDBsum	EXONUCLEASE RECJ	D<br>D<br>D<br>D	A 326<br>A 389<br>A 392<br>A 393	matches<br>matches<br>matches<br>matches	A 221<br>A 136<br>A 84<br>A 82	-2.7 A from MN MN A1434s<br>-2.4 A from MN MN A1434s<br>-3.4 A from MN MN A1434s<br>4.4 A from MN MN A1434	1.39 A	Submit
5grq<br>PDB<br>PDBsum	DEATH DOMAIN-ASSOCIATED PROTEIN 6	D<br>D<br>D<br>D	A 326<br>A 389<br>A 392<br>A 393	matches<br>matches<br>matches<br>matches	C1263<br>A 80<br>C1261<br>C1262	3.9 A from ZN ZN C1303<br>4.0 A from ZN ZN C1303s<br>2.3 A from ZN ZN C1302s<br>-2.5 A from ZN ZN C1303s	1.39 A	Submit
4fgq<br>PDB<br>PDBsum	PERIPLASMIC PROTEIN	D<br>D<br>D<br>D	A 326<br>A 389<br>A 392<br>A 393	matches<br>matches<br>matches<br>matches	A 121<br>A 113<br>A 120<br>A 136	none<br>none<br>none<br>none	1.39 A	Submit
1yo8<br>PDB<br>PDBsum	THROMBOSPONDIN-2	D<br>D<br>D<br>D	A 326<br>A 389<br>A 392<br>A 393	matches<br>matches<br>matches<br>matches	A 741<br>A 751<br>A 744<br>A 745	-2.4 A from CA CA A1207s<br>-3.2 A from CA CA A1208s<br>-2.4 A from CA CA A1208s<br>2.6 A from CA CA A1205s	1.39 A	Submit
4npj<br>PDB<br>PDBsum	EXTENDED SYNAPTOTAGMIN-2	D<br>D<br>D<br>D	A 326<br>A 389<br>A 392<br>A 393	matches<br>matches<br>matches<br>matches	A 467<br>A 464<br>A 462<br>A 466	17.9 A from CL CL A 801<br>20.7 A from CL CL A 801<br>18.1 A from CL CL A 801<br>14.2 A from CL CL A 801	1.39 A	Submit
3x17<br>PDB<br>PDBsum	ENDOGLUCANASE	D<br>D<br>D<br>D	A 326<br>A 389<br>A 392<br>A 393	matches<br>matches<br>matches<br>matches	A 356<br>A 354<br>A 353<br>A 351	-3.0 A from CA CA A 603s<br>7.3 A from CA CA A 603<br>-5.9 A from CA CA A 603s<br>-2.0 A from CA CA A 603s	1.39 A	Submit
3pdd<br>PDB<br>PDBsum	GLYCOSIDE HYDROLASE, FAMILY 9	D<br>D<br>D<br>D	A 326<br>A 389<br>A 392<br>A 393	matches<br>matches<br>matches<br>matches	A 121<br>A 164<br>A 119<br>A 163	-2.9 A from CA CA A 193s<br>5.4 A from CA CA A 193<br>-2.1 A from CA CA A 193s<br>-3.2 A from CA CA A 193s	1.40 A	Submit
3isa<br>PDB<br>PDBsum	PUTATIVE ENOYL-COA HYDRATASE/ISOMERASE	D<br>D<br>D<br>D	A 326<br>A 389<br>A 392<br>A 393	matches<br>matches<br>matches<br>matches	A 216<br>A 219<br>A 218<br>A 221	5.9 A from CL CL A 262<br>-5.2 A from CL CL A 262s<br>7.3 A from CL CL A 262<br>6.9 A from CL CL A 262	1.40 A	Submit
1h6g<br>PDB<br>PDBsum	ALPHA-1 CATENIN	D<br>D<br>D<br>D	A 326<br>A 389<br>A 392<br>A 393	matches<br>matches<br>matches<br>matches	A 500<br>A 510<br>A 503<br>A 504	-4.2 A from CA CA A1632s<br>4.3 A from CA CA A1632<br>6.0 A from CA CA A1632<br>4.1 A from CA CA A1632	1.40 A	Submit
5gne<br>PDB<br>PDBsum	LEUCINE AMINOPEPTIDASE	D<br>D<br>D<br>D	A 326<br>A 389<br>A 392<br>A 393	matches<br>matches<br>matches<br>matches	A 288<br>A 226<br>A 227<br>A 225	-1.9 A from ZN ZN A 402s<br>2.8 A from ZN ZN A 402s<br>4.8 A from ZN ZN A 402<br>8.2 A from ZN ZN A 402	1.40 A	Submit
1exn<br>PDB<br>PDBsum	5'-EXONUCLEASE	D<br>D<br>D<br>D	A 326<br>A 389<br>A 392<br>A 393	matches<br>matches<br>matches<br>matches	A 204<br>A 130<br>A 155<br>A 153	none<br>none<br>none<br>none	1.40 A	Submit

4py9<br>PDB<br>PDBsum	PUTATIVE EXOPOLYPHOSPHATASE-RELATED PROTEIN	D<br>D<br>D<br>D	A 326 matches A 99<br>A 389 matches A 33<br>A 392 matches A 35<br>A 393 matches A 177	5.0 A from O3 PO4 A 401<br>-3.1 A from NA NA A 406s<br>-5.5 A from NA NA A 406s<br>2.5 A from O3 PO4 A 402s	1.40 A	Submit
5hkq<br>PDB<br>PDBsum	CONTACT-DEPENDENT INHIBITOR A	D<br>D<br>D<br>D	A 326 matches A 311<br>A 389 matches I 80<br>A 392 matches I 78<br>A 393 matches I 81	none<br>none<br>none<br>none	1.40 A	Submit
3wiu<br>PDB<br>PDBsum	TK-SUBTILISIN	D<br>D<br>D<br>D	A 326 matches A 216<br>A 389 matches A 225<br>A 392 matches A 214<br>A 393 matches A 224	2.9 A from CA CA A1003s<br>-3.3 A from CA CA A1003s<br>2.9 A from CA CA A1004s<br>-3.4 A from CA CA A1004s	1.40 A	Submit
4v29<br>PDB<br>PDBsum	AT3G17980	D<br>D<br>D<br>D	A 326 matches A 87<br>A 389 matches A 93<br>A 392 matches A 92<br>A 393 matches A 85	2.3 A from CA CA A1178s<br>4.5 A from CA CA A1178<br>7.0 A from CA CA A1179<br>2.4 A from CA CA A1179s	1.40 A	Submit
3a3o<br>PDB<br>PDBsum	TK-SUBTILISIN	D<br>D<br>D<br>D	A 326 matches A 216<br>A 389 matches A 224<br>A 392 matches A 222<br>A 393 matches A 225	2.4 A from CA CA A 4s<br>5.4 A from CA CA A 4<br>2.2 A from CA CA A 4s<br>-3.2 A from CA CA A 3s	1.41 A	Submit
5yh1<br>PDB<br>PDBsum	MEMBER OF S1P FAMILY OF RIBOSOMAL PROTEINS	D<br>D<br>D<br>D	A 326 matches A 528<br>A 389 matches A 436<br>A 392 matches A 366<br>A 393 matches A 364	-2.6 A from MN MN A 805s<br>-2.5 A from MN MN A 805s<br>-3.1 A from MN MN A 805s<br>4.7 A from MN MN A 805	1.41 A	Submit
2f3o<br>PDB<br>PDBsum	PYRUVATE FORMATE-LYASE 2	D<br>D<br>D<br>D	A 326 matches A 595<br>A 389 matches A 597<br>A 392 matches A 600<br>A 393 matches A 596	29.5 A from O1 GOL A 778<br>31.1 A from O1 GOL A 778<br>27.0 A from O1 GOL A 778<br>32.6 A from O1 GOL A 778	1.41 A	Submit
5xsp<br>PDB<br>PDBsum	PHOSPHODIESTERASE ACTING ON CYCLIC DINUCLEOTIDES	D<br>D<br>D<br>D	A 326 matches A 418<br>A 389 matches A 347<br>A 392 matches A 349<br>A 393 matches A 497	2.4 A from MN MN A 701s<br>-2.5 A from MN MN A 702s<br>-3.3 A from MN MN A 701s<br>-2.8 A from MN MN A 701s	1.41 A	Submit
4l9d<br>PDB<br>PDBsum	PROTEASE	D<br>D<br>D<br>D	A 326 matches A 822<br>A 389 matches A 782<br>A 392 matches A 821<br>A 393 matches A 780	7.2 A from NA NA A 901<br>8.2 A from NA NA A 901<br>8.5 A from NA NA A 901<br>-5.2 A from NA NA A 901s	1.41 A	Submit
3ge2<br>PDB<br>PDBsum	LIPOPROTEIN, PUTATIVE	D<br>D<br>D<br>D	A 326 matches A 147<br>A 389 matches A 142<br>A 392 matches A 140<br>A 393 matches A 139	5.4 A from O1 GOL A 204<br>-3.4 A from O1 GOL A 203s<br>-3.6 A from O3 GOL A 203s<br>7.3 A from O1 GOL A 206	1.41 A	Submit
5o25<br>PDB<br>PDBsum	TMPDE	D<br>D<br>D<br>D	A 326 matches A 80<br>A 389 matches A 23<br>A 392 matches A 25<br>A 393 matches A 154	2.6 A from MN MN A 401s<br>-2.0 A from MN MN A 401s<br>-3.1 A from MN MN A 402s<br>-2.8 A from MN MN A 402s	1.41 A	Submit
5izo<br>PDB<br>PDBsum	BIFUNCTIONAL OLIGORIBONUCLEASE AND PAP PHOSPHATASE	D<br>D<br>D<br>D	A 326 matches A 80<br>A 389 matches A 24<br>A 392 matches A 26<br>A 393 matches A 156	-2.6 A from MN MN A 402s<br>2.1 A from MN MN A 401s<br>-3.0 A from MN MN A 402s<br>-2.7 A from MN MN A 402s	1.42 A	Submit
5ovo<br>PDB<br>PDBsum	ADP-RIBOSYL-(DINITROGEN REDUCTASE) HYDROLASE	D<br>D<br>D<br>D	A 326 matches A 243<br>A 389 matches A 60<br>A 392 matches A 245<br>A 393 matches A 61	-3.3 A from MG MG A 302s<br>3.7 A from MG MG A 302<br>-2.6 A from MG MG A 302s<br>4.1 A from MG MG A 302	1.42 A	Submit
1rtq<br>PDB<br>PDBsum	BACTERIAL LEUCYL AMINOPEPTIDASE	D<br>D<br>D<br>D	A 326 matches A 118<br>A 389 matches A 116<br>A 392 matches A 117<br>A 393 matches A 260	0.6 A from CG ASP A 118<br>0.5 A from CG ASP A 116<br>0.6 A from CG ASP A 117s<br>0.6 A from CG ASP A 260	1.42 A	Submit
5gne<br>PDB<br>PDBsum	LEUCINE AMINOPEPTIDASE	D<br>D<br>D<br>D	A 326 matches A 288<br>A 389 matches A 215<br>A 392 matches A 227<br>A 393 matches A 226	-1.9 A from ZN ZN A 402s<br>7.2 A from ZN ZN A 401<br>4.8 A from ZN ZN A 402<br>2.8 A from ZN ZN A 402s	1.42 A	Submit
5xso	RESPONSE REGULATOR FIXJ	D	A 326 matches A 13	7.1 A from O1 FMT A 407	1.43 A	Submit

PDB<br>PDBsum		D<br>D<br>D	A 389 matches A 55<br>A 392 matches A 11<br>A 393 matches A 12	6.2 A from O1 FMT A 405<br>7.5 A from O2 FMT A 405<br>-2.9 A from O1 FMT A 405s		
2wnx<br>PDB<br>PDBsum	GLYCOSIDE HYDROLASE, FAMILY 9	D<br>D<br>D<br>D	A 326 matches A 129<br>A 389 matches A 125<br>A 392 matches A 128<br>A 393 matches A 57	-3.0 A from CA CA A1163s<br>-5.9 A from CA CA A1163s<br>-3.1 A from CA CA A1163s<br>-2.2 A from CA CA A1163s	1.43 A	Submit
3p4g<br>PDB<br>PDBsum	ANTIFREEZE PROTEIN	D<br>D<br>D<br>D	A 326 matches A 177<br>A 389 matches A 196<br>A 392 matches A 179<br>A 393 matches A 200	-5.8 A from CA CA A 407s<br>-6.2 A from CA CA A 408s<br>2.4 A from CA CA A 407s<br>2.4 A from CA CA A 408s	1.43 A	Submit
4ls9<br>PDB<br>PDBsum	DHH FAMILY PROTEIN	D<br>D<br>D<br>D	A 326 matches A 110<br>A 389 matches A 49<br>A 392 matches A 51<br>A 393 matches A 185	-2.2 A from MN MN A1001s<br>4.8 A from MN MN A1001<br>-3.2 A from MN MN A1001s<br>-3.0 A from MN MN A1001s	1.43 A	Submit
4iik<br>PDB<br>PDBsum	ADENOSINE MONOPHOSPHATE-PROTEIN HYDROLASE SIDD	D<br>D<br>D<br>D	A 326 matches A 326<br>A 389 matches A 91<br>A 392 matches A 327<br>A 393 matches A 110	-3.6 A from MG MG A 402s<br>5.0 A from MG MG A 401<br>5.5 A from MG MG A 401<br>2.8 A from MG MG A 401s	1.43 A	Submit
5hsa<br>PDB<br>PDBsum	ALCOHOL OXIDASE 1	D<br>D<br>D<br>D	A 326 matches A 389<br>A 389 matches A 110<br>A 392 matches A 108<br>A 393 matches A 111	12.6 A from CA CA A 705<br>14.4 A from CA CA A 705<br>13.5 A from CA CA A 705<br>8.0 A from CA CA A 705	1.43 A	Submit
5l3e<br>PDB<br>PDBsum	LYSINE-SPECIFIC HISTONE DEMETHYLASE 1A	D<br>D<br>D<br>D	A 326 matches A 553<br>A 389 matches A 557<br>A 392 matches A 556<br>A 393 matches A 555	4.8 A from CBI E11 A 902<br>-4.2 A from CAX E11 A 905s<br>2.8 A from N3 E11 A 903s<br>4.1 A from CAQ E11 A 902	1.44 A	Submit
4rj9<br>PDB<br>PDBsum	C2 DOMAIN-CONTAINING PROTEIN-LIKE	D<br>D<br>D<br>D	A 326 matches A 76<br>A 389 matches A 23<br>A 392 matches A 74<br>A 393 matches A 28	4.6 A from K K A 202<br>4.5 A from K K A 201<br>-2.2 A from K K A 201s<br>-3.4 A from K K A 201s	1.44 A	Submit
3gin<br>PDB<br>PDBsum	SODIUM/CALCIUM EXCHANGER 1	D<br>D<br>D<br>D	A 326 matches A 447<br>A 389 matches A 498<br>A 392 matches A 500<br>A 393 matches A 499	-3.0 A from CA CA A 7s<br>-2.3 A from CA CA A 1s<br>2.4 A from CA CA A 2s<br>-2.8 A from CA CA A 2s	1.44 A	Submit
5cyb<br>PDB<br>PDBsum	LIPOPROTEIN	D<br>D<br>D<br>D	A 326 matches A 146<br>A 389 matches A 141<br>A 392 matches A 139<br>A 393 matches A 138	-4.5 A from CA CA A 203s<br>-2.6 A from CA CA A 205s<br>4.9 A from CA CA A 205<br>10.5 A from O1 GOL A 201	1.44 A	Submit
5jh9<br>PDB<br>PDBsum	VACUOLAR AMINOPEPTIDASE 1	D<br>D<br>D<br>D	A 326 matches A 385<br>A 389 matches A 134<br>A 392 matches A 304<br>A 393 matches A 303	2.0 A from ZN ZN A 601s<br>6.7 A from O1 CAC A 603<br>5.0 A from ZN ZN A 601<br>-2.4 A from ZN ZN A 601s	1.44 A	Submit
4rj9<br>PDB<br>PDBsum	C2 DOMAIN-CONTAINING PROTEIN-LIKE	D<br>D<br>D<br>D	A 326 matches A 76<br>A 389 matches A 81<br>A 392 matches A 80<br>A 393 matches A 74	4.6 A from K K A 202<br>-3.1 A from K K A 202s<br>7.1 A from K K A 201<br>-2.2 A from K K A 201s	1.44 A	Submit
4a01<br>PDB<br>PDBsum	PROTON PYROPHOSPHATASE	D<br>D<br>D<br>D	A 326 matches A 269<br>A 389 matches A 253<br>A 392 matches A 279<br>A 393 matches A 283	3.9 A from MG MG A1768<br>-2.4 A from MG MG A1767s<br>4.3 A from MG MG A1768<br>2.7 A from MG MG A1770s	1.44 A	Submit
5cet<br>PDB<br>PDBsum	BIFUNCTIONAL OLIGORIBONUCLEASE AND PAP PHOSPHATASE	D<br>D<br>D<br>D	A 326 matches A 181<br>A 389 matches A 106<br>A 392 matches A 47<br>A 393 matches A 45	-2.6 A from MN MN A1001s<br>2.5 A from MN MN A1001s<br>-3.1 A from MN MN A1001s<br>-1.9 A from MN MN A1002s	1.45 A	Submit
3eqz<br>PDB<br>PDBsum	RESPONSE REGULATOR	D<br>D<br>D<br>D	A 326 matches A 52<br>A 389 matches A 10<br>A 392 matches A 9<br>A 393 matches A 11	none<br>none<br>none<br>none	1.45 A	Submit
4lvn<br>PDB<br>PDBsum	SUBTILISIN-LIKE SERINE PROTEASE	D<br>D<br>D	A 326 matches A 409<br>A 389 matches A 400<br>A 392 A 402	-2.8 A from CA CA A 704s<br>-3.2 A from CA CA A 704s<br>-2.9 A from CA CA A 704s	1.45 A	Submit

		D	A 393	matches<br>matches	A 401	-3.1 A from CA CA A 703s		
4c7i<br>PDB<br>PDBsum	GLYCYLPEPTIDE N-TETRADECANOYLTRANSFERASE	D<br>D<br>D<br>D	A 326<br>A 389<br>A 392<br>A 393	matches<br>matches<br>matches<br>matches	A 85<br>A 396<br>A 83<br>A 84	-3.7 A from N JJ1 A1424s<br>-3.4 A from N JJ1 A1424s<br>-3.9 A from N JJ1 A1424s<br>-4.7 A from O3 JJ1 A1424s	1.45 A	Submit
5iz5<br>PDB<br>PDBsum	CYTOSOLIC PHOSPHOLIPASE A2 DELTA	D<br>D<br>D<br>D	A 326<br>A 389<br>A 392<br>A 393	matches<br>matches<br>matches<br>matches	A 102<br>A 96<br>A 94<br>A 101	18.4 A from O3 SO4 A 903<br>21.3 A from O3 SO4 A 903<br>19.8 A from O3 SO4 A 903<br>14.6 A from O3 SO4 A 903	1.45 A	Submit
5f56<br>PDB<br>PDBsum	SINGLE-STRANDED-DNA-SPECIFIC EXONUCLEASE	D<br>D<br>D<br>D	A 326<br>A 389<br>A 392<br>A 393	matches<br>matches<br>matches<br>matches	A 135<br>A 81<br>A 83<br>A 223	2.6 A from MN MN A 805s<br>-1.9 A from MN MN A 804s<br>-3.3 A from MN MN A 805s<br>-2.6 A from MN MN A 805s	1.46 A	Submit
5wtl<br>PDB<br>PDBsum	OMPA FAMILY PROTEIN	D<br>D<br>D<br>D	A 326<br>A 389<br>A 392<br>A 393	matches<br>matches<br>matches<br>matches	A 308<br>A 318<br>A 316<br>A 319	-3.2 A from CA CA A1009s<br>9.3 A from CA CA A1009<br>5.4 A from CA CA A1009<br>2.1 A from CA CA A1010s	1.46 A	Submit
1ux6<br>PDB<br>PDBsum	THROMBOSPONDIN-1	D<br>D<br>D<br>D	A 326<br>A 389<br>A 392<br>A 393	matches<br>matches<br>matches<br>matches	A 890<br>A 879<br>A 881<br>A 880	2.2 A from CA CA A2005s<br>3.1 A from CA CA A2004s<br>2.8 A from CA CA A2004s<br>-2.2 A from CA CA A2008s	1.46 A	Submit
1yo8<br>PDB<br>PDBsum	THROMBOSPONDIN-2	D<br>D<br>D<br>D	A 326<br>A 389<br>A 392<br>A 393	matches<br>matches<br>matches<br>matches	A 910<br>A 899<br>A 901<br>A 900	2.3 A from CA CA A1186s<br>3.3 A from CA CA A1185s<br>2.8 A from CA CA A1185s<br>-2.4 A from CA CA A1189s	1.46 A	Submit
3eqz<br>PDB<br>PDBsum	RESPONSE REGULATOR	D<br>D<br>D	A 326<br>A 389<br>A 392	matches<br>matches<br>matches	A 11<br>A 52<br>A 9	none<br>none<br>none	1.46 A	Submit

Back
