# Supplementary material for: Torpedo californica acetylcholinesterase is stabilized by binding of a divalent metal ion to a novel and versatile 4D motif
Source: Protein Sci. 2021 Mar 29;30(5):966–81. doi: 10.1002/pro.4061 (PMC8040873; doi:10.1002/pro.4061)
Supplement: Supplementary file 2 — Table S2 Output from the ASSAM server of proteins whose 3D structures contains the 3D1E motif based on the crystal structure of BfAChE. [file PRO-30-966-s003.pdf]

GrAfSS GRaph theoretic Applications for Structure Searching
Molecular Function Regulation Lab.
M. Firdaus Raih Research Group
Universiti Kebangsaan Malaysia
The National University of Malaysia
KREBS INSTITUTE
The University Of Sheffield.

ASSAM
Amino acid pattern Search for Substructures And Motifs

IMAAAGINE SPRITE ASSAM NASSAM

About GrAfSS Contact us HOME

Results of ASSAM search and righthanded superposition for 4qww_a_rot_on_1ea5_3D_1E_motif
→ Download text version of the ASSAM output

Matches found in 4qww_a_rot_on_1ea5_3D_1E_motif (PDB ID)	Description	Residues	Residue Matches Query Database Hits	Heteroatoms Notes in Database hit	RMSD	Viewer
6cgs<br>PDB<br>PDBsum	CADHERIN-7	D<br>E<br>D<br>D	A matches A 134<br>326 matches A 103<br>A matches A 132<br>389 matches A 187<br>A<br>392<br>A<br>393	3.3 A from CA CA A 302s<br>9.0 A from CA CA A 303<br>-2.2 A from CA CA A 303s<br>-3.3 A from CA CA A 303s	1.07 A	Submit
1u7o<br>PDB<br>PDBsum	MAGNESIUM-DEPENDENT PHOSPHATASE-1	D<br>E<br>D<br>D	A matches A 11<br>326 matches A 124<br>A matches A 122<br>389 matches A 123<br>A<br>392<br>A<br>393	12.7 A from O ACT A 166<br>10.5 A from OXT ACT A 166<br>12.1 A from OXT ACT A 166<br>12.8 A from OXT ACT A 165	1.09 A	Submit
2iq1<br>PDB<br>PDBsum	PROTEIN PHOSPHATASE 2C KAPPA, PPM1K	D<br>E<br>D<br>D	A matches A 298<br>326 matches A 108<br>A matches A 127<br>389 matches A 109<br>A<br>392<br>A<br>393	3.6 A from MG MG A 1<br>5.5 A from MG MG A 1<br>-2.8 A from MG MG A 1s<br>4.9 A from MG MG A 1	1.16 A	Submit
2isn<br>PDB<br>PDBsum	NYSGXRC-8828Z, PHOSPHATASE	D<br>E<br>D<br>D	A matches A 277<br>326 matches A 28<br>A matches A 50<br>389 matches A 29<br>A<br>392<br>A<br>393	-2.5 A from PR PR A 522s<br>7.3 A from PR PR A 522<br>-2.5 A from PR PR A 522s<br>5.0 A from PR PR A 522	1.16 A	Submit
5m6g<br>PDB<br>PDBsum	BETA-GLUCOSIDASE	D<br>E<br>D<br>D	A matches A 312<br>326 matches A 321<br>A matches A 315<br>389 matches A 322<br>A<br>392<br>A<br>393	9.1 A from C5 SOR A 711<br>9.2 A from MG MG A 701<br>-6.0 A from MG MG A 701s<br>-7.2 A from MG MG A 701s	1.17 A	Submit

5osw<br>PDB<br>PDBsum	ALBUMIN	D<br>E<br>D<br>D	A 326 matches A 13<br>A 389 matches A 251<br>A 392 matches A 254<br>A 393 matches A 255	15.5 A from I2 DIU A 601<br>16.2 A from O1 DIU A 601<br>12.7 A from I2 DIU A 601<br>11.1 A from I2 DIU A 601	1.23 A	Submit
3wh2<br>PDB<br>PDBsum	C-TYPE LECTIN DOMAIN FAMILY 4 MEMBER E	D<br>E<br>D<br>D	A 326 matches A 194<br>A 389 matches A 147<br>A 392 matches A 178<br>A 393 matches A 143	3.3 A from CA CA A 301s<br>12.9 A from OB1 FLC A 303<br>6.3 A from CA CA A 301<br>12.5 A from CA CA A 301	1.25 A	Submit
2pnq<br>PDB<br>PDBsum	[PYRUVATE DEHYDROGENASE [LIPOAMIDE]]-PHOSPHATASE	D<br>E<br>D<br>D	A 326 matches A 73<br>A 389 matches A 53<br>A 392 matches A 54<br>A 393 matches A 446	2.6 A from MG MG A 502s<br>4.9 A from MG MG A 501<br>4.6 A from MG MG A 502<br>5.3 A from MG MG A 501	1.26 A	Submit
4csw<br>PDB<br>PDBsum	CUPIN 4 FAMILY PROTEIN	D<br>E<br>D<br>D	A 326 matches A 117<br>A 389 matches A 93<br>A 392 matches A 118<br>A 393 matches A 95	23.3 A from CL1 UN9 A1392<br>21.0 A from C6 UN9 A1391<br>19.4 A from CL1 UN9 A1392<br>18.3 A from C6 UN9 A1392	1.29 A	Submit
3g20<br>PDB<br>PDBsum	TYPE II SECRETION PROTEIN	D<br>E<br>D<br>D	A 326 matches A 117<br>A 389 matches A 123<br>A 392 matches A 124<br>A 393 matches A 125	-2.9 A from CA CA A 201s<br>8.5 A from CA CA A 201<br>-5.2 A from CA CA A 201s<br>-3.3 A from CA CA A 201s	1.29 A	Submit
4s17<br>PDB<br>PDBsum	GLUTAMINE SYNTHETASE	D<br>E<br>D<br>D	A 326 matches A 141<br>A 389 matches A 170<br>A 392 matches A 171<br>A 393 matches A 172	21.0 A from MG MG A 501<br>26.0 A from MG MG A 501<br>26.8 A from MG MG A 501<br>29.5 A from MG MG A 501	1.31 A	Submit
4fgq<br>PDB<br>PDBsum	PERIPLASMIC PROTEIN	D<br>E<br>D<br>D	A 326 matches A 120<br>A 389 matches A 111<br>A matches A 136<br>A matches A 139	none<br>none<br>none<br>none	1.31 A	Submit

			392 A 393			
4a01 PDB PDBsum	PROTON PYROPHOSPHATASE	D E D D	A 326 matches A 691 A 389 matches A 698 A 392 matches A 723 A 393 matches A 257	2.7 A from MG MG A1771s 6.8 A from O4 2PN A1773 3.9 A from MG MG A1769 -2.5 A from MG MG A1767s	1.32 A	Submit
2zyr PDB PDBsum	LIPASE, PUTATIVE	D E D D	A 326 matches A 409 A 389 matches A 345 A 392 matches A 405 A 393 matches A 431	-2.9 A from MG MG A2003s 8.0 A from MG MG A2003 -3.3 A from MG MG A2003s -2.4 A from MG MG A2003s	1.32 A	Submit
3cih PDB PDBsum	PUTATIVE ALPHA-RHAMNOSIDASE	D E D D	A 326 matches A 337 A 389 matches A 597 A 392 matches A 332 A 393 matches A 344	4.3 A from O3 TRS A 800 -3.7 A from O1 TRS A 800s -3.6 A from O3 TRS A 800s -2.6 A from N TRS A 800s	1.34 A	Submit
5msm PDB PDBsum	SISTER CHROMATID COHESION PROTEIN DCC1	D E D D	A 326 matches A 299 A 389 matches A 342 A 392 matches A 343 A 393 matches A 340	none none none none	1.34 A	Submit
3cu5 PDB PDBsum	TWO COMPONENT TRANSCRIPTIONAL REGULATOR, ARAC FAMIL	D E D D	A 326 matches A 56 A 389 matches A 11 A 392 matches A 9 A 393 matches A 10	none none none none	1.36 A	Submit
5wrt PDB PDBsum	SOLUBLE INORGANIC PYROPHOSPHATASE	D E D D	A 326 matches A 192 A 389 matches A 225 A 392 matches A 190 A 393 matches A 227	5.4 A from MG MG A 402 7.9 A from MG MG A 401 -3.0 A from MG MG A 401s -2.8 A from MG MG A 401s	1.37 A	Submit
4xlz PDB PDBsum	UNCHARACTERIZED PROTEIN	D E D D	A 326 matches A 150 A 389 matches A 113 A 392 matches A 111 matches A 69	7.8 A from CD CD A 301 13.8 A from C5 HEZ A 308	1.37 A	Submit

			A<br>393	7.3 A from CD CD A 301<br>12.2 A from CD CD A 301		
4mlb<br>PDB<br>PDBsum	PF0708	D<br>E<br>D<br>D	A matches A 226<br>326 matches A 91<br>A matches A 94<br>389 matches A 231<br>A<br>392<br>A<br>393	9.8 A from C12 CXE A 504<br>16.5 A from C19 CXE A 504<br>11.7 A from C12 CXE A 504<br>11.6 A from C6 CXE A 504	1.37 A	Submit
1a76<br>PDB<br>PDBsum	FLAP ENDONUCLEASE-1 PROTEIN	D<br>E<br>D<br>D	A matches A 173<br>326 matches A 154<br>A matches A 175<br>389 matches A 224<br>A<br>392<br>A<br>393	4.9 A from MN MN A 341<br>-2.6 A from MN MN A 340s<br>-3.8 A from MN MN A 341s<br>-3.0 A from MN MN A 341s	1.38 A	Submit
2bce<br>PDB<br>PDBsum	CHOLESTEROL ESTERASE	D<br>E<br>D<br>D	A matches A 186<br>326 matches A 179<br>A matches A 184<br>389 matches A 97<br>A<br>392<br>A<br>393	none<br>none<br>none<br>none	1.39 A	Submit
1lns<br>PDB<br>PDBsum	X-PROLYL DIPEPTIDYL AMINOPEPTIDASE	D<br>E<br>D<br>D	A matches A 178<br>326 matches A 180<br>A matches A 186<br>389 matches A 296<br>A<br>392<br>A<br>393	none<br>none<br>none<br>none	1.40 A	Submit
5cuy<br>PDB<br>PDBsum	ACIDOCALCISOMAL PYROPHOSPHATASE	D<br>E<br>D<br>D	A matches A 293<br>326 matches A 326<br>A matches A 291<br>389 matches A 328<br>A<br>392<br>A<br>393	5.0 A from MG MG A 902<br>9.5 A from MG MG A 902<br>-3.2 A from MG MG A 902s<br>-4.3 A from MG MG A 902s	1.40 A	Submit
3ory<br>PDB<br>PDBsum	FLAP ENDONUCLEASE 1	D<br>E<br>D<br>D	A matches A 179<br>326 matches A 94<br>A matches A 177<br>389 matches A 31<br>A<br>392<br>A<br>393	4.6 A from O4 PO4 A 354<br>-3.4 A from O1 PO4 A 354s<br>-2.8 A from O4 PO4 A 354s<br>-2.8 A from O3 PO4 A 354s	1.41 A	Submit
2dew<br>PDB<br>PDBsum	PROTEIN-ARGININE DEIMINASE TYPE IV	D<br>E<br>D	A matches X 388<br>326 matches X 175<br>A X 179	-3.1 A from CA CA X 902s	1.42 A	Submit

		D	389<br>A<br>392<br>A<br>393	matches<br>matches	X 165	8.5 A from CA CA X 902<br>2.3 A from CA CA X 902s<br>3.3 A from CA CA X 901s		
3kh1<br>PDB<br>PDBsum	PREDICTED METAL-DEPENDENT PHOSPHOHYDROLASE	D<br>E<br>D<br>D	A<br>326<br>A<br>389<br>A<br>392<br>A<br>393	matches<br>matches<br>matches<br>matches	A 75<br>A 38<br>A 40<br>A 83	12.1 A from CA CA A 200<br>8.0 A from CA CA A 200<br>11.7 A from CA CA A 200<br>8.6 A from CA CA A 200	1.42 A	Submit
2akz<br>PDB<br>PDBsum	GAMMA ENOLASE	D<br>E<br>D<br>D	A<br>326<br>A<br>389<br>A<br>392<br>A<br>393	matches<br>matches<br>matches<br>matches	A 244<br>A 249<br>A 293<br>A 318	-2.5 A from MG MG A 440s<br>-3.3 A from O3 TRS A 460s<br>5.0 A from MG MG A 440<br>3.9 A from MG MG A 441	1.43 A	Submit
5jbe<br>PDB<br>PDBsum	INACTIVE GLUCANSUCRASE	D<br>E<br>D<br>D	A<br>326<br>A<br>389<br>A<br>392<br>A<br>393	matches<br>matches<br>matches<br>matches	A1108<br>A1111<br>A1190<br>A1380	25.6 A from CH3 ACT A1706<br>32.6 A from CH3 ACT A1706<br>25.6 A from CH3 ACT A1706<br>25.7 A from CH3 ACT A1706	1.44 A	Submit
2o4v<br>PDB<br>PDBsum	PORIN P	D<br>E<br>D<br>D	A<br>326<br>A<br>389<br>A<br>392<br>A<br>393	matches<br>matches<br>matches<br>matches	A 176<br>A 252<br>A 174<br>A 212	15.3 A from C16 C8E A1297<br>21.2 A from C16 C8E A1297<br>12.0 A from C16 C8E A1297<br>17.5 A from C16 C8E A1297	1.44 A	Submit
4g1q<br>PDB<br>PDBsum	REVERSE TRANSCRIPTASE/RIBONUCLEASE H	D<br>E<br>D<br>D	A<br>326<br>A<br>389<br>A<br>392<br>A<br>393	matches<br>matches<br>matches<br>matches	A 549<br>A 478<br>A 443<br>A 498	-3.1 A from MG MG A 608s<br>7.1 A from MG MG A 608<br>-2.6 A from MG MG A 608s<br>4.1 A from MG MG A 608	1.45 A	Submit
3ujg<br>PDB<br>PDBsum	SERINE/THREONINE-PROTEIN KINASE SRK2E	D<br>E<br>D<br>D	A<br>326<br>A<br>389<br>A<br>392	matches<br>matches<br>matches<br>matches	B 243<br>B 203<br>B 204<br>B 492	2.6 A from MG MG B 512s<br>5.7 A from MG MG B 100	1.45 A	Submit

			A 393	-4.1 A from MG MG B 100s<br>-2.3 A from MG MG B 512s		
5msn<br>PDB<br>PDBsum	DCC1 PROTEIN	D<br>E<br>D<br>D	A 326 matches A 299<br>A 389 matches A 342<br>A 392 matches A 343<br>A 393 matches A 340	none<br>none<br>none<br>none	1.45 A	Submit
5jbd<br>PDB<br>PDBsum	INACTIVE GLUCANSUCRASE	D<br>E<br>D<br>D	A 326 matches A1108<br>A 389 matches A1111<br>A 392 matches A1190<br>A 393 matches A1380	16.8 A from O ACT A1702<br>12.6 A from O ACT A1702<br>17.9 A from O ACT A1702<br>18.3 A from O ACT A1702	1.45 A	Submit
2gre<br>PDB<br>PDBsum	DEBLOCKING AMINOPEPTIDASE	D<br>E<br>D<br>D	A 326 matches A 186<br>A 389 matches A 220<br>A 392 matches A 187<br>A 393 matches A 241	29.8 A from O3 SO4 A 350<br>27.4 A from O3 SO4 A 350<br>23.9 A from O3 SO4 A 350<br>24.5 A from O3 SO4 A 350	1.45 A	Submit
1e9g<br>PDB<br>PDBsum	INORGANIC PYROPHOSPHATASE	D<br>E<br>D<br>D	A 326 matches A 117<br>A 389 matches A 150<br>A 392 matches A 115<br>A 393 matches A 147	-3.5 A from O4 BPO4 A3002s<br>7.7 A from O1 BPO4 A3001<br>2.8 A from MN MN A1001s<br>3.2 A from MN MN A1004s	1.47 A	Submit
5i8d<br>PDB<br>PDBsum	CADHERIN-23	D<br>E<br>D<br>D	A 326 matches A2041<br>A 389 matches A2073<br>A 392 matches A2074<br>A 393 matches A2072	2.6 A from CA CA A2302s<br>9.4 A from CA CA A2304<br>3.3 A from CA CA A2303s<br>-2.4 A from CA CA A2304s	1.47 A	Submit
3kwu<br>PDB<br>PDBsum	MUNC13-1	D<br>E<br>D<br>D	A 326 matches A 705<br>A 389 matches A 758<br>A 392 matches A 759<br>A 393 matches A 775	2.2 A from CA CA A 901s<br>-7.3 A from CA CA A 901s<br>2.2 A from CA CA A 902s<br>-3.7 A from O3 AGOL A 921s	1.47 A	Submit

2hih PDB PDBsum	LIPASE 46 KDA FORM	D E D D	A 326 matches A 365<br>A 389 matches A 292<br>A 392 matches A 357<br>A 393 matches A 354	-3.6 A from CA CA A 603s<br>7.6 A from CA CA A 603<br>-2.3 A from CA CA A 603s<br>-2.8 A from CA CA A 603s	1.48 A	Submit
4grh PDB PDBsum	AMINODEOXYCHORISMATE SYNTHASE	D E D D	A 326 matches A 284<br>A 389 matches A 281<br>A 392 matches A 429<br>A 393 matches A 283	-2.6 A from MG MG A 507s<br>4.9 A from MG MG A 507<br>4.7 A from MG MG A 507<br>7.9 A from MG MG A 507	1.48 A	Submit
4g3h PDB PDBsum	ARGINASE (ROCF)	D E D D	A 326 matches A 120<br>A 389 matches A 250<br>A 392 matches A 116<br>A 393 matches A 234	-2.6 A from MN MN A 500s<br>7.6 A from MN MN A 501<br>2.5 A from MN MN A 501s<br>2.8 A from MN MN A 501s	1.48 A	Submit
1w6t PDB PDBsum	ENOLASE	D E D D	A 326 matches A 242<br>A 389 matches A 247<br>A 392 matches A 292<br>A 393 matches A 319	-2.5 A from MG MG A 435s<br>8.5 A from MG MG A 435<br>5.0 A from MG MG A 435<br>5.4 A from MG MG A 435	1.48 A	Submit
3klk PDB PDBsum	GLUCANSUCRASE	D E D D	A 326 matches A1119<br>A 389 matches A1122<br>A 392 matches A1198<br>A 393 matches A1401	25.3 A from O3 GOL A1785<br>30.8 A from O3 GOL A1783<br>24.3 A from O3 GOL A1783<br>24.6 A from C1 GOL A1783	1.49 A	Submit
3slr PDB PDBsum	UNCHARACTERIZED PROTEIN BF1531	D E D D	A 326 matches A 230<br>A 389 matches A 340<br>A 392 matches A 228<br>A 393 matches A 336	-4.6 A from MG MG A 389s<br>7.1 A from MG MG A 389<br>2.7 A from MG MG A 388s<br>-2.6 A from MG MG A 388s	1.49 A	Submit
2dew PDB PDBsum	PROTEIN-ARGININE DEIMINASE TYPE IV	D E D	A 326 matches X 179<br>A matches X 175<br>X 176	2.3 A from CA CA X 902s	1.49 A	Submit

		D	389 matches X 165<br>A matches<br>392<br>A<br>393	8.5 A from CA CA X 902<br>-3.4 A from CA CA X 901s<br>3.3 A from CA CA X 901s		
4gwm<br>PDB<br>PDBsum	MEPRIN A SUBUNIT BETA	D<br>E<br>D<br>D	A matches A 419<br>326 matches A 268<br>A matches A 418<br>389 matches A 298<br>A<br>392<br>A<br>393	4.7 A from NA NA A 702<br>3.8 A from NA NA A 702s<br>-2.4 A from NA NA A 702s<br>-3.5 A from NA NA A 702s	1.49 A	Submit
4rzy<br>PDB<br>PDBsum	PEPTIDASE M24	D<br>E<br>D<br>D	A matches A 295<br>326 matches A 264<br>A matches A 307<br>389 matches A 377<br>A<br>392<br>A<br>393	-2.0 A from FE FE A 501s<br>6.6 A from O1S MES A 503<br>2.4 A from FE FE A 501s<br>-3.0 A from O3S MES A 503s	1.49 A	Submit
1lw7<br>PDB<br>PDBsum	TRANSCRIPTIONAL REGULATOR NADR	D<br>E<br>D<br>D	A matches A 99<br>326 matches A 139<br>A matches A 95<br>389 matches A 140<br>A<br>392<br>A<br>393	7.2 A from O3D NAD A 601<br>-2.5 A from O3D NAD A 601s<br>7.6 A from O2D NAD A 601<br>9.9 A from O3D NAD A 601	1.50 A	Submit
5cuv<br>PDB<br>PDBsum	ACIDOCALCISOMAL PYROPHOSPHATASE	D<br>E<br>D<br>D	A matches A 296<br>326 matches A 278<br>A matches A 328<br>389 matches A 291<br>A<br>392<br>A<br>393	-2.7 A from MG MG A 502s<br>6.5 A from MG MG A 502<br>-3.4 A from MG MG A 502s<br>-2.9 A from MG MG A 502s	1.50 A	Submit
1e9g<br>PDB<br>PDBsum	INORGANIC PYROPHOSPHATASE	D<br>E<br>D<br>D	A matches A 117<br>326 matches A 150<br>A matches A 115<br>389 matches A 152<br>A<br>392<br>A<br>393	-3.5 A from O4 BPO4 A3002s<br>7.7 A from O1 BPO4 A3001<br>2.8 A from MN MN A1001s<br>-2.6 A from MN MN A1001s	1.51 A	Submit
1u7o<br>PDB<br>PDBsum	MAGNESIUM-DEPENDENT PHOSPHATASE-1	D<br>E<br>D<br>D	A matches A 13<br>326 matches A 124<br>A matches A 11<br>389 matches A 123<br>A<br>392	10.7 A from OXT ACT A 165<br>10.5 A from OXT ACT A 166	1.51 A	Submit

			A 393	12.7 A from O ACT A 166<br>12.8 A from OXT ACT A 165		
3ikw<br>PDB<br>PDBsum	HEPARIN LYASE I	D<br>E<br>D<br>D	A 326 matches A 155<br>A 389 matches A 347<br>A 392 matches A 348<br>A 393 matches A 346	9.9 A from CA CA A 1<br>8.0 A from O1 EDO A 380<br>9.4 A from CA CA A 1<br>-3.3 A from CA CA A 1s	1.51 A	Submit
2i7h<br>PDB<br>PDBsum	NITROREDUCTASE-LIKE FAMILY PROTEIN	D<br>E<br>D<br>D	A 326 matches A 100<br>A 389 matches A 108<br>A 392 matches A 109<br>A 393 matches A 107	21.1 A from C7M FMN A1001<br>19.7 A from C7M FMN A1001<br>14.8 A from C7M FMN A1001<br>14.5 A from C7M FMN A1001	1.52 A	Submit
5uc2<br>PDB<br>PDBsum	DOMAIN OF UNKNOWN FUNCTION DUF1849	D<br>E<br>D<br>D	A 326 matches A 106<br>A 389 matches A 100<br>A 392 matches A 101<br>A 393 matches A 103	17.7 A from O1 EDO A 302<br>11.3 A from O2 GOL A 301<br>19.5 A from O1 EDO A 302<br>20.5 A from O2 GOL A 301	1.52 A	Submit
4a9v<br>PDB<br>PDBsum	PHOX	D<br>E<br>D<br>D	A 326 matches A 438<br>A 389 matches A 279<br>A 392 matches A 281<br>A 393 matches A 282	9.3 A from LI LI A 612<br>-1.6 A from LI LI A 612s<br>8.1 A from LI LI A 612<br>9.0 A from LI LI A 612	1.52 A	Submit
3zsc<br>PDB<br>PDBsum	PECTATE TRISACCHARIDE-LYASE	D<br>E<br>D<br>D	A 326 matches A 115<br>A 389 matches A 122<br>A 392 matches A 117<br>A 393 matches A 143	6.9 A from O6B AQA A1338<br>13.0 A from O6B AQA A1338<br>8.0 A from O6B AQA A1338<br>6.8 A from O6B AQA A1338	1.53 A	Submit
2ehg<br>PDB<br>PDBsum	RIBONUCLEASE HI	D<br>E<br>D<br>D	A 326 matches A 125<br>A 389 matches A 52<br>A 392 matches A 7<br>A 393 matches A 76	none<br>none<br>none<br>none	1.54 A	Submit
5unc<br>PDB<br>PDBsum	PHOSPHOENOLPYRUVATE PHOSPHOMUTASE	D<br>E<br>D<br>D	A 326 matches A 71<br>A 389 matches A 127<br>A 392 matches A 100<br>matches A 98	-3.1 A from O2 TLA A 401s<br>4.6 A from O1 TLA A 401	1.54 A	Submit

			A 393	6.1 A from O1 TLA A 401<br>-3.3 A from O3 TLA A 401s		
2e7s<br>PDB<br>PDBsum	RAB GUANINE NUCLEOTIDE EXCHANGE FACTOR SEC2	D<br>E<br>D<br>D	A 326 matches A 70<br>A 389 matches A 72<br>A 392 matches A 71<br>A 393 matches A 68	none<br>none<br>none<br>none	1.54 A	Submit
4u65<br>PDB<br>PDBsum	TWO COMPONENT HISTIDINE KINASE, GGDEF DOMAIN PROTEI	D<br>E<br>D<br>D	A 326 matches E 119<br>A 389 matches E 122<br>A 392 matches E 111<br>A 393 matches E 134	-3.3 A from CA CA E 302s<br>5.2 A from CA CA E 301<br>2.3 A from CA CA E 301s<br>2.6 A from CA CA E 302s	1.54 A	Submit
5e33<br>PDB<br>PDBsum	DIPEPTIDYL PEPTIDASE 3	D<br>E<br>D<br>D	A 326 matches A 431<br>A 389 matches A 429<br>A 392 matches A 430<br>A 393 matches A 433	26.2 A from ZN ZN A 801<br>31.3 A from MG MG A 802<br>31.1 A from ZN ZN A 801<br>30.3 A from ZN ZN A 801	1.55 A	Submit
5e6s<br>PDB<br>PDBsum	INTEGRIN ALPHA-L	D<br>E<br>D<br>D	A 326 matches A 569<br>A 389 matches A 567<br>A 392 matches A 565<br>A 393 matches A 573	-2.7 A from CA CA A2004s<br>-5.3 A from CA CA A2004s<br>-3.3 A from CA CA A2004s<br>-2.9 A from CA CA A2004s	1.56 A	Submit
2jk1<br>PDB<br>PDBsum	HYDROGENASE TRANSCRIPTIONAL REGULATORY PROTEIN HUPR	D<br>E<br>D<br>D	A 326 matches A 55<br>A 389 matches A 14<br>A 392 matches A 12<br>A 393 matches A 13	-2.8 A from MG MG A1144s<br>4.6 A from MG MG A1144<br>4.6 A from MG MG A1144<br>-2.4 A from MG MG A1144s	1.56 A	Submit
3cux<br>PDB<br>PDBsum	MALATE SYNTHASE	D<br>E<br>D<br>D	A 326 matches A 443<br>A 389 matches A 448<br>A 392 matches A 117<br>A 393 matches A 275	5.8 A from MG MG A 1<br>10.4 A from MG MG A 1<br>4.7 A from MG MG A 1<br>-3.3 A from MG MG A 1s	1.57 A	Submit
4g3h<br>PDB	ARGINASE (ROCF)	D<br>E	A 326 matches A 120<br>matches A 250	-2.6 A from MN MN A	1.57 A	Submit

PDBsum		D<br>D	A matches A 116<br>389 matches A 236<br>A<br>392<br>A<br>393	500s<br>7.6 A from MN MN A 501<br>2.5 A from MN MN A 501s<br>-2.3 A from MN MN A 501s		
5gw8<br>PDB<br>PDBsum	HYPOTHETICAL SECRETORY LIPASE (FAMILY 3)	D<br>E<br>D<br>D	A matches A 279<br>326 matches A 282<br>A matches A 280<br>389 matches A 228<br>A<br>392<br>A<br>393	-3.4 A from O6 NAG A 403s<br>-4.5 A from C8 NAG A 403s<br>-3.9 A from N2 NAG A 402s<br>10.0 A from O7 NAG A 403	1.57 A	Submit
3ruv<br>PDB<br>PDBsum	CHAPERONIN	D<br>E<br>D<br>D	A matches A 60<br>326 matches A 86<br>A matches A 386<br>389 matches A 385<br>A<br>392<br>A<br>393	2.6 A from MG MG A 549s<br>5.4 A from MG MG A 549<br>-3.1 A from MG MG A 549s<br>6.6 A from MG MG A 549	1.58 A	Submit
2dew<br>PDB<br>PDBsum	PROTEIN-ARGININE DEIMINASE TYPE IV	D<br>E<br>D<br>D	A matches X 155<br>326 matches X 175<br>A matches X 179<br>389 matches X 176<br>A<br>392<br>A<br>393	2.8 A from CA CA X 901s<br>8.5 A from CA CA X 902<br>2.3 A from CA CA X 902s<br>-3.4 A from CA CA X 901s	1.58 A	Submit
1dc1<br>PDB<br>PDBsum	DNA (5'-D(*T*AP*TP*AP*CP*TP*CP*GP*AP*GP*TP*AP*T)-	D<br>E<br>D<br>D	A matches A 151<br>326 matches A 175<br>A matches A 177<br>389 matches A 176<br>A<br>392<br>A<br>393	16.4 A from C2 DIO A1002<br>22.7 A from C2' DIO A1002<br>14.4 A from C2 DIO A1002<br>17.7 A from C2 DIO A1002	1.58 A	Submit
4rhz<br>PDB<br>PDBsum	CRY23AA1	D<br>E<br>D<br>D	A matches B 83<br>326 matches B 81<br>A matches B 88<br>389 matches B 89<br>A<br>392<br>A<br>393	12.3 A from CA CA B 501<br>8.2 A from CA CA B 501<br>10.0 A from CA CA B 501<br>-2.7 A from CA CA B 501s	1.58 A	Submit
4af1<br>PDB<br>PDBsum	PEPTIDE CHAIN RELEASE FACTOR SUBUNIT 1	D<br>E<br>D<br>D	A matches A 373<br>326 matches A 23<br>A matches A 370<br>389 matches A 329<br>A	26.9 A from ZN ZN A 500<br>29.3 A from ZN ZN A	1.59 A	Submit

			392<br>A<br>393	500<br>25.5 A from ZN ZN A 500<br>22.7 A from ZN ZN A 500		
2isn<br>PDB<br>PDBsum	NYSGXRC-8828Z, PHOSPHATASE	D<br>E<br>D<br>D	A 326 matches A 323<br>A 389 matches A 28<br>A 392 matches A 50<br>A 393 matches A 29	-2.4 A from PR PR A 522s<br>7.3 A from PR PR A 522<br>-2.5 A from PR PR A 522s<br>5.0 A from PR PR A 522	1.59 A	Submit
5fi9<br>PDB<br>PDBsum	SPHINGOMYELIN PHOSPHODIESTERASE	D<br>E<br>D<br>D	A 326 matches A 289<br>A 389 matches A 210<br>A 392 matches A 281<br>A 393 matches A 208	7.1 A from H62 NAG A 704<br>15.2 A from H62 NAG A 704<br>11.4 A from HO4 NAG A 704<br>12.8 A from O13 NT8 A 718	1.59 A	Submit
3bij<br>PDB<br>PDBsum	UNCHARACTERIZED PROTEIN GSU0716	D<br>E<br>D<br>D	A 326 matches A 100<br>A 389 matches A 114<br>A 392 matches A 112<br>A 393 matches A 113	none<br>none<br>none<br>none	1.59 A	Submit
5f7u<br>PDB<br>PDBsum	CYCLOALTERNAN-FORMING ENZYME	D<br>E<br>D<br>D	A 326 matches A 694<br>A 389 matches A 401<br>A 392 matches A 403<br>A 393 matches A 407	5.8 A from MG MG A1104<br>-6.4 A from MG MG A1104s<br>-2.9 A from MG MG A1104s<br>-2.8 A from MG MG A1104s	1.59 A	Submit
4a01<br>PDB<br>PDBsum	PROTON PYROPHOSPHATASE	D<br>E<br>D<br>D	A 326 matches A 727<br>A 389 matches A 268<br>A 392 matches A 253<br>A 393 matches A 279	2.5 A from MG MG A1769s<br>-3.7 A from O4 2PN A1773s<br>-2.4 A from MG MG A1767s<br>4.3 A from MG MG A1768	1.60 A	Submit
3lwu<br>PDB<br>PDBsum	SUCCINYLGLUTAMATE DESUCCINYLASE/ASPARTOACYLASE	D<br>E<br>D<br>D	A 326 matches A 251<br>A 389 matches A 257<br>A 392 matches A 254<br>A 393 matches A 258	17.4 A from O12 UNL A 373<br>21.8 A from O6 UNL A 373<br>15.3 A from O6 UNL A 373<br>13.7 A from O6 UNL A	1.60 A	Submit

				373		
1bgx PDB PDBsum	TAQ DNA POLYMERASE	D E D D	A 326 matches T 144<br>A 389 matches T 117<br>A 392 matches T 119<br>A 393 matches T 120	none none none none	1.60 A	Submit
3qml PDB PDBsum	78 KDA GLUCOSE-REGULATED PROTEIN HOMOLOG	D E D D	A 326 matches C 395<br>A 389 matches C 390<br>A 392 matches C 392<br>A 393 matches C 388	25.7 A from O3 PO4 C 6<br>27.5 A from O3 PO4 C 6<br>31.9 A from O3 PO4 C 6<br>29.8 A from O3 PO4 C 6	1.60 A	Submit
5wrt PDB PDBsum	SOLUBLE INORGANIC PYROPHOSPHATASE	D E D D	A 326 matches A 195<br>A 389 matches A 174<br>A 392 matches A 227<br>A 393 matches A 190	2.7 A from MG MG A 401s<br>6.7 A from MG MG A 401<br>-2.8 A from MG MG A 401s<br>-3.0 A from MG MG A 401s	1.60 A	Submit
5o0s PDB PDBsum	GLUCOSYLCERAMIDASE	D E D D	A 326 matches A 579<br>A 389 matches A 657<br>A 392 matches A 577<br>A 393 matches A 583	-2.8 A from CA CA A 910s<br>8.4 A from CA CA A 910<br>-3.1 A from CA CA A 910s<br>-3.2 A from CA CA A 910s	1.60 A	Submit
4pvc PDB PDBsum	NADPH-DEPENDENT METHYLGLYOXAL REDUCTASE GRE2	D E D D	A 326 matches A 282<br>A 389 matches A 326<br>A 392 matches A 329<br>A 393 matches A 330	none none none none	1.60 A	Submit
3mse PDB PDBsum	CALCIUM-DEPENDENT PROTEIN KINASE, PUTATIVE	D E D D	A 326 matches B 119<br>A 389 matches B 120<br>A 392 matches B 121<br>A 393 matches B 117	5.9 A from CA CA B 180<br>9.7 A from CA CA B 180<br>-2.3 A from CA CA B 180s<br>-3.3 A from CA CA B 180s	1.60 A	Submit
2cm5 PDB PDBsum	RABPHILIN-3A	D E D D	A 326 matches A 577<br>A 389 matches A 529<br>A 392 matches A 571<br>A 393 matches A 639	-3.4 A from CA CA A1679s<br>-5.2 A from CA CA A1679s<br>2.2 A from CA CA A1679s<br>-3.3 A from CA CA	1.61 A	Submit

				A1678s		
1h30 PDB PDBsum	GROWTH-ARREST-SPECIFIC PROTEIN	D E D D	A 326 matches A 654<br>A 389 matches A 331<br>A 392 matches A 656<br>A 393 matches A 329	4.8 A from CA CA A 701<br>-6.4 A from CA CA A 701s<br>-3.4 A from CA CA A 701s<br>-2.4 A from CA CA A 701s	1.61 A	Submit
5ij7 PDB PDBsum	ENHANCER OF ZESTE HOMOLOG 2 (EZH2),HISTONE-LYSINE N	D E D D	A 326 matches S 582<br>A 389 matches S 584<br>A 392 matches S 585<br>A 393 matches S 583	14.1 A from C21 6BN A9001<br>19.2 A from C21 6BN A9001<br>15.8 A from C22 6BN A9001<br>21.2 A from C21 6BN A9001	1.61 A	Submit
5uly PDB PDBsum	PROTOCADHERIN-15	D E D D	A 326 matches A 236<br>A 389 matches A 202<br>A 392 matches A 289<br>A 393 matches A 239	-3.1 A from CA CA A1001s<br>6.1 A from CA CA A1001<br>3.0 A from CA CA A1001s<br>-3.3 A from CA CA A1001s	1.61 A	Submit
5azx PDB PDBsum	TRANSMEMBRANE EMP24 DOMAIN-CONTAINING PROTEIN 10	D E D D	A 326 matches A 78<br>A 389 matches A 100<br>A 392 matches A 101<br>A 393 matches A 103	19.2 A from O4 SO4 A 201<br>19.2 A from O4 SO4 A 201<br>19.2 A from O2 SO4 A 201<br>14.6 A from O3 SO4 A 201	1.61 A	Submit
1yo8 PDB PDBsum	THROMBOSPONDIN-2	D E D D	A 326 matches A 797<br>A 389 matches A 795<br>A 392 matches A 789<br>A 393 matches A 791	5.6 A from CA CA A1197<br>-5.7 A from CA CA A1197s<br>-3.0 A from CA CA A1197s<br>-2.9 A from CA CA A1197s	1.62 A	Submit
4gua PDB PDBsum	NON-STRUCTURAL POLYPROTEIN	D E D D	A 326 matches A1284<br>A 389 matches A1522<br>A 392 matches A1521<br>A 393 matches A1520	10.7 A from O3 SO4 A1709<br>6.1 A from O4 SO4 A1701<br>4.8 A from O3 SO4 A1709<br>8.0 A from O1 SO4 A1709	1.62 A	Submit

5vxz PDB PDBsum	GROWTH ARREST-SPECIFIC PROTEIN 6	D E D D	A 326 matches A 654<br>A 389 matches A 331<br>A 392 matches A 656<br>A 393 matches A 329	4.8 A from CA CA A 703<br>-6.3 A from CA CA A 703s<br>-3.0 A from CA CA A 703s<br>-2.2 A from CA CA A 703s	1.62 A	Submit
5osw PDB PDBsum	ALBUMIN	D E D D	A 326 matches A 13<br>A 389 matches A 6<br>A 392 matches A 254<br>A 393 matches A 1	15.5 A from I2 DIU A 601<br>18.7 A from I2 DIU A 601<br>12.7 A from I2 DIU A 601<br>17.4 A from I2 DIU A 601	1.62 A	Submit
3b77 PDB PDBsum	UNCHARACTERIZED PROTEIN	D E D D	A 326 matches A 96<br>A 389 matches A 90<br>A 392 matches A 98<br>A 393 matches A 113	none<br>none<br>none<br>none	1.62 A	Submit
1e9g PDB PDBsum	INORGANIC PYROPHOSPHATASE	D E D D	A 326 matches A 120<br>A 389 matches A 101<br>A 392 matches A 152<br>A 393 matches A 115	-2.5 A from MN MN A1001s<br>6.6 A from MN MN A1001<br>-2.6 A from MN MN A1001s<br>2.8 A from MN MN A1001s	1.62 A	Submit
5f7u PDB PDBsum	CYCLOALTERNAN-FORMING ENZYME	D E D D	A 326 matches A1077<br>A 389 matches A1000<br>A 392 matches A1078<br>A 393 matches A1073	15.9 A from O6 GLC A1113<br>14.8 A from O6 GLC A1113<br>12.5 A from O6 GLC A1113<br>13.6 A from O4 GLC A1109	1.62 A	Submit
5f1m PDB PDBsum	PHOSPHORYLATED PROTEIN PHOSPHATASE	D E D D	A 326 matches A 233<br>A 389 matches A 18<br>A 392 matches A 37<br>A 393 matches A 19	-2.4 A from MN MN A 301s<br>-3.0 A from MN MN A 303s<br>2.5 A from MN MN A 301s<br>4.7 A from MN MN A 302	1.63 A	Submit
2vn6 PDB PDBsum	SCAFFOLDING PROTEIN	D E D D	A 326 matches B 49<br>A 389 matches B 44<br>A 392 matches B 38<br>A 393 matches B 42	-2.2 A from CA CA B1066s<br>-7.5 A from CA CA B1066s<br>-3.3 A from CA CA B1066s<br>-2.9 A from CA CA	1.63 A	Submit

				B1066s		
5b0o<br>PDB<br>PDBsum	FLAGELLUM-SPECIFIC ATP SYNTHASE	D<br>E<br>D<br>D	A matches A 160<br>326 matches A 443<br>A matches A 441<br>389 matches A 444<br>A<br>392<br>A<br>393	16.4 A from<br>N6 ADP A<br>601<br>22.7 A from<br>N6 ADP A<br>601<br>18.0 A from<br>N6 ADP A<br>601<br>16.3 A from<br>N6 ADP A<br>601	1.63 A	Submit
3u3g<br>PDB<br>PDBsum	RIBONUCLEASE H	D<br>E<br>D<br>D	A matches A 128<br>326 matches A 49<br>A matches A 9<br>389 matches A 77<br>A<br>392<br>A<br>393	7.6 A from<br>CL CL A 201<br>-3.6 A from<br>CL CL A<br>201s<br>-3.2 A from<br>CL CL A<br>201s<br>5.3 A from<br>CL CL A 201	1.63 A	Submit
4hsr<br>PDB<br>PDBsum	GLUTARYL-7-AMINOCEPHALOSPORANIC ACID ACYLASE ALPHA	D<br>E<br>D	A matches A 193<br>326 matches A 206<br>A matches A 197<br>389<br>A<br>392	19.3 A from<br>OE2 GLJ B<br>601<br>21.8 A from<br>OE2 GLJ B<br>601<br>15.7 A from<br>OE2 GLJ B<br>601	1.63 A	Submit

Results of ASSAM search and lefthanded superposition for 4qww_a_rot_on_1ea5_3D_1E_motif

➔ Download text version of the ASSAM output

Matches found in 4qww_a_rot_on_1ea5_3D_1E_motif (PDB ID)	Description	Residues	Residue Matches Query Database Hits	Heteroatoms Notes in Database hit	RMSD	
3t8j<br>PDB<br>PDBsum	PURINE NUCLEOSIDASE, (IUNH-1)	D<br>E<br>D<br>D	A matches A 9<br>326 matches A<br>A matches 12<br>389 matches A<br>A 14<br>392 A<br>A 238<br>393	-3.2 A from<br>NA NA A<br>401s<br>8.5 A from<br>NA NA A<br>401<br>-2.6 A from<br>NA NA A<br>401s<br>3.9 A from<br>NA NA A<br>401	1.14 A	Submit
4a7c<br>PDB<br>PDBsum	PROTO-ONCOGENE SERINE/THREONINE-PROTEIN KINASE PIM-	D<br>E<br>D<br>D	A matches A<br>326 matches 131<br>A matches A<br>389 matches 171<br>A A<br>392 128<br>A A<br>393 170	5.2 A from<br>CAJ E46<br>A1306<br>-6.5 A from<br>CAK E46<br>A1306s<br>-3.5 A from<br>NAR E46<br>A1306s<br>-3.5 A from<br>N3 IMD<br>A1307s	1.21 A	Submit
3f2a<br>PDB<br>PDBsum	PROTO-ONCOGENE SERINE/THREONINE-PROTEIN KINASE	D<br>E<br>D<br>D	A matches A<br>326 matches 131<br>A matches A<br>389 matches 171<br>A A	6.0 A from<br>C14 985 A 1<br>-5.0 A from<br>C5 985 A 1s<br>-4.7 A from	1.22 A	Submit

			392 ... 128<br>A ... A<br>393 ... 170	C14 985 A 1s<br>9.2 A from C14 985 A 1		
2p3y<br>PDB<br>PDBsum	HYPOTHETICAL PROTEIN VPA0735	D<br>E<br>D<br>D	A 326 matches A 371<br>A 389 matches A 480<br>A 392 matches A 369<br>A 393 matches A 478	none<br>none<br>none<br>none	1.22 A	Submit
3r04<br>PDB<br>PDBsum	PROTO-ONCOGENE SERINE/THREONINE-PROTEIN KINASE PIM-	D<br>E<br>D<br>D	A 326 matches A 131<br>A 389 matches A 171<br>A 392 matches A 128<br>A 393 matches A 170	5.2 A from C23 UNQ A 555<br>-3.5 A from N3 IMD A4498s<br>-4.0 A from N26 UNQ A 555s<br>-3.7 A from N1 IMD A4497s	1.22 A	Submit
2rhq<br>PDB<br>PDBsum	PHENYLALANYL-TRNA SYNTHETASE ALPHA CHAIN	D<br>E<br>D<br>D	A 326 matches B 740<br>A 389 matches B 773<br>A 392 matches B 771<br>A 393 matches B 774	none<br>none<br>none<br>none	1.22 A	Submit
4a01<br>PDB<br>PDBsum	PROTON PYROPHOSPHATASE	D<br>E<br>D<br>D	A 326 matches A 283<br>A 389 matches A 268<br>A 392 matches A 279<br>A 393 matches A 253	2.7 A from MG MG A1770s<br>-3.7 A from O4 2PN A1773s<br>4.3 A from MG MG A1768<br>-2.4 A from MG MG A1767s	1.23 A	Submit
3fgy<br>PDB<br>PDBsum	UNCHARACTERIZED NTF2-LIKE PROTEIN	D<br>E<br>D<br>D	A 326 matches A 121<br>A 389 matches A 99<br>A 392 matches A 100<br>A 393 matches A 101	9.5 A from O3 UNL A 135<br>10.7 A from O3 UNL A 135<br>-3.7 A from O3 UNL A 135s<br>9.7 A from O3 UNL A 135	1.24 A	Submit
2dew<br>PDB<br>PDBsum	PROTEIN-ARGININE DEIMINASE TYPE IV	D<br>E<br>D<br>D	A 326 matches X 165<br>A 389 matches X 175<br>A 392 matches X 176<br>A 393 matches X 179	3.3 A from CA CA X 901s<br>8.5 A from CA CA X 902<br>-3.4 A from CA CA X 901s<br>2.3 A from CA CA X 902s	1.24 A	Submit
5tur<br>PDB<br>PDBsum	SERINE/THREONINE-PROTEIN KINASE PIM-1	D<br>E<br>D<br>D	A 326 matches A 131<br>A 389 matches A 171<br>A ... A	-3.0 A from CAV 7LK A 401s<br>5.2 A from CAT 7LK A	1.24 A	Submit

			392<br>A<br>393 128<br>A<br>170	401<br>3.9 A from<br>CAV 7LK A<br>401<br>9.0 A from<br>CAT 7LK A<br>401		
1xws<br>PDB<br>PDBsum	PROTO-ONCOGENE SERINE/THREONINE-PROTEIN KINASE PIM-	D<br>E<br>D<br>D	A matches A<br>326 matches 131<br>A matches A<br>389 matches 171<br>A A<br>392 128<br>A A<br>393 170	6.7 A from<br>CBE BI1<br>A1001<br>-4.3 A from<br>CBD BI1<br>A1001s<br>5.1 A from<br>CBE BI1<br>A1001<br>8.7 A from<br>CBD BI1<br>A1001	1.25 A	Submit
1yo8<br>PDB<br>PDBsum	THROMBOSPONDIN-2	D<br>E<br>D<br>D	A matches A<br>326 matches 791<br>A matches A<br>389 matches 795<br>A A<br>392 789<br>A A<br>393 797	-2.9 A from<br>CA CA<br>A1197s<br>-5.7 A from<br>CA CA<br>A1197s<br>-3.0 A from<br>CA CA<br>A1197s<br>5.6 A from<br>CA CA<br>A1197	1.26 A	Submit
5xd0<br>PDB<br>PDBsum	GLUCANASE	D<br>E<br>D<br>D	A matches A<br>326 matches 293<br>A matches A<br>389 matches 285<br>A A<br>392 267<br>A A<br>393 289	8.0 A from<br>C1 PGE A<br>501<br>4.4 A from<br>C4 PGE A<br>501<br>7.5 A from<br>C3 PGE A<br>501<br>10.0 A from<br>C4 PGE A<br>501	1.26 A	Submit
1t5j<br>PDB<br>PDBsum	HYPOTHETICAL PROTEIN MJ1187	D<br>E<br>D<br>D	A matches A<br>326 matches 62<br>A matches A<br>389 matches 64<br>A A<br>392 61<br>A A<br>393 97	-3.6 A from<br>MG B MG A<br>500s<br>11.2 A from<br>MG A MG A<br>500<br>-3.0 A from<br>MG A MG A<br>500s<br>4.9 A from<br>MG A MG A<br>500	1.28 A	Submit
4iqb<br>PDB<br>PDBsum	THYMIDYLATE SYNTHASE	D<br>E<br>D<br>D	A matches A<br>326 matches 150<br>A matches A<br>389 matches 129<br>A A<br>392 132<br>A A<br>393 99	24.4 A from<br>O3 SO4 A<br>401<br>23.9 A from<br>O3 SO4 A<br>401<br>18.6 A from<br>O3 SO4 A<br>401<br>21.2 A from<br>O3 SO4 A<br>401	1.28 A	Submit
3iij<br>PDB<br>PDBsum	COILIN-INTERACTING NUCLEAR ATPASE PROTEIN	D<br>E<br>D<br>D	A matches A<br>326 matches 45<br>A matches A<br>389 matches 58<br>A A<br>392 57<br>A A	24.6 A from<br>O2B ADP A<br>173<br>17.9 A from<br>O2B ADP A<br>173<br>22.3 A from<br>O3B ADP A	1.31 A	Submit

			393 59	173<br>22.9 A from O1 SO4 A 175		
5uly<br>[PDB](PDB)<br>PDBsum	PROTOCADHERIN-15	D<br>E<br>D<br>D	A 326 matches A 239<br>A 389 matches A 202<br>A 392 matches A 289<br>A 393 matches A 236	-3.3 A from CA CA A1001s<br>6.1 A from CA CA A1001<br>3.0 A from CA CA A1001s<br>-3.1 A from CA CA A1001s	1.32 A	Submit
3kwu<br>[PDB](PDB)<br>PDBsum	MUNC13-1	D<br>E<br>D<br>D	A 326 matches A 705<br>A 389 matches A 758<br>A 392 matches A 759<br>A 393 matches A 761	2.2 A from CA CA A 901s<br>-7.3 A from CA CA A 901s<br>2.2 A from CA CA A 902s<br>6.9 A from CA CA A 901	1.33 A	Submit
5vxz<br>[PDB](PDB)<br>PDBsum	GROWTH ARREST-SPECIFIC PROTEIN 6	D<br>E<br>D<br>D	A 326 matches A 329<br>A 389 matches A 331<br>A 392 matches A 656<br>A 393 matches A 654	-2.2 A from CA CA A 703s<br>-6.3 A from CA CA A 703s<br>-3.0 A from CA CA A 703s<br>4.8 A from CA CA A 703	1.35 A	Submit
4qtq<br>[PDB](PDB)<br>PDBsum	XAC2610 PROTEIN	D<br>E<br>D<br>D	A 326 matches A 155<br>A 389 matches A 158<br>A 392 matches A 151<br>A 393 matches A 159	-2.9 A from CA CA A 301s<br>7.9 A from CA CA A 301<br>-3.2 A from CA CA A 301s<br>-2.3 A from CA CA A 301s	1.36 A	Submit
3k3q<br>[PDB](PDB)<br>PDBsum	LLAMA AA1 VHH DOMAIN	D<br>E<br>D<br>D	A 326 matches A 128<br>A 389 matches A 114<br>A 392 matches A 113<br>A 393 matches A 115	none<br>none<br>none<br>none	1.36 A	Submit
2akz<br>[PDB](PDB)<br>PDBsum	GAMMA ENOLASE	D<br>E<br>D<br>D	A 326 matches A 317<br>A 389 matches A 249<br>A 392 matches A 293<br>A 393 matches A 244	-3.0 A from MG MG A 440s<br>-3.3 A from O3 TRS A 460s<br>5.0 A from MG MG A 440<br>-2.5 A from MG MG A 440s	1.37 A	Submit
3oyz	MALATE SYNTHASE	D	A matches A	2.9 A from	1.37 A	Submit

PDB<br>PDBsum		E<br>D<br>D	326 matches 192<br>A matches A<br>389 matches 389<br>A A<br>392 52<br>A A<br>393 388	MG MG A<br>500s<br>10.4 A from<br>MG MG A<br>500<br>-5.1 A from<br>MG MG A<br>500s<br>-3.6 A from<br>C ACO A<br>434s		
1yo8<br>PDB<br>PDBsum	THROMBOSPONDIN-2	D<br>E<br>D<br>D	A matches A<br>326 matches 939<br>A matches A<br>389 matches 950<br>A A<br>392 946<br>A A<br>393 943	2.7 A from<br>CA CA<br>A1183s<br>10.0 A from<br>CA CA<br>A1182<br>2.2 A from<br>CA CA<br>A1182s<br>5.7 A from<br>CA CA<br>A1183	1.38 A	Submit
3kh1<br>PDB<br>PDBsum	PREDICTED METAL-DEPENDENT PHOSPHOHYDROLASE	D<br>E<br>D<br>D	A matches A<br>326 matches 83<br>A matches A<br>389 matches 38<br>A A<br>392 40<br>A A<br>393 75	8.6 A from<br>CA CA A<br>200<br>8.0 A from<br>CA CA A<br>200<br>11.7 A from<br>CA CA A<br>200<br>12.1 A from<br>CA CA A<br>200	1.40 A	Submit
1vq8<br>PDB<br>PDBsum	23S RIBOSOMAL RNA	D<br>E<br>D<br>D	A matches T<br>326 matches 102<br>A matches T<br>389 matches 61<br>A T<br>392 46<br>A T<br>393 100	17.1 A from<br>MG MG<br>T8073<br>23.2 A from<br>MG MG<br>T8073<br>13.9 A from<br>MG MG<br>T8073<br>16.8 A from<br>MG MG<br>T8073	1.40 A	Submit
3kwu<br>PDB<br>PDBsum	MUNC13-1	D<br>E<br>D<br>D	A matches A<br>326 matches 705<br>A matches A<br>389 matches 758<br>A A<br>392 757<br>A A<br>393 775	2.2 A from<br>CA CA A<br>901s<br>-7.3 A from<br>CA CA A<br>901s<br>2.7 A from<br>CA CA A<br>901s<br>-3.7 A from<br>O3 AGOL A<br>921s	1.40 A	Submit
2f96<br>PDB<br>PDBsum	RIBONUCLEASE T	D<br>E<br>D<br>D	A matches A<br>326 matches 137<br>A matches A<br>389 matches 37<br>A A<br>392 35<br>A A<br>393 198	4.1 A from<br>MG MG<br>A2001<br>6.9 A from<br>MG MG<br>A2001<br>-2.5 A from<br>MG MG<br>A2001s<br>5.3 A from<br>MG MG<br>A2001	1.41 A	Submit
1ux6<br>PDB<br>PDBsum	THROMBOSPONDIN-1	D<br>E<br>D	A matches A<br>326 matches 919<br>A matches A<br>389 matches 930	2.7 A from<br>CA CA<br>A2002s<br>9.8 A from	1.41 A	Submit

		D	A<br>392<br>A<br>393		A<br>926<br>A<br>923	CA CA<br>A2001<br>2.2 A from<br>CA CA<br>A2001s<br>5.6 A from<br>CA CA<br>A2002		
4g3h<br>PDB<br>PDBsum	ARGINASE (ROCF)	D<br>E<br>D<br>D	A<br>326<br>A<br>389<br>A<br>392<br>A<br>393	matches<br>matches<br>matches<br>matches	A<br>120<br>A<br>281<br>A<br>116<br>A<br>236	-2.6 A from<br>MN MN A<br>500s<br>5.8 A from<br>MN MN A<br>500<br>2.5 A from<br>MN MN A<br>501s<br>-2.3 A from<br>MN MN A<br>501s	1.41 A	Submit
2db3<br>PDB<br>PDBsum	5'-<br>R(*UP*UP*UP*UP*UP*UP*UP*UP*UP*U)-3'	D<br>E<br>D<br>D	A<br>326<br>A<br>389<br>A<br>392<br>A<br>393	matches<br>matches<br>matches<br>matches	A<br>571<br>A<br>438<br>A<br>402<br>A<br>406	11.5 A from<br>O1G ANP<br>A2901<br>18.2 A from<br>O1G ANP<br>A2901<br>8.9 A from<br>O1G ANP<br>A2901<br>16.8 A from<br>O1G ANP<br>A2901	1.42 A	Submit
5wtl<br>PDB<br>PDBsum	OMPA FAMILY PROTEIN	D<br>E<br>D<br>D	A<br>326<br>A<br>389<br>A<br>392<br>A<br>393	matches<br>matches<br>matches<br>matches	A<br>312<br>A<br>323<br>A<br>319<br>A<br>316	2.7 A from<br>CA CA<br>A1009s<br>7.5 A from<br>CA CA<br>A1010<br>2.1 A from<br>CA CA<br>A1010s<br>5.4 A from<br>CA CA<br>A1009	1.42 A	Submit
4p7o<br>PDB<br>PDBsum	POLY-BETA-1,6-N-ACETYL-D-<br>GLUCOSAMINE N-DEACETYLASE	D<br>E<br>D<br>D	A<br>326<br>A<br>389<br>A<br>392<br>A<br>393	matches<br>matches<br>matches<br>matches	A<br>362<br>A<br>367<br>A<br>358<br>A<br>322	none<br>none<br>none<br>none	1.42 A	Submit
4fgq<br>PDB<br>PDBsum	PERIPLASMIC PROTEIN	D<br>E<br>D<br>D	A<br>326<br>A<br>389<br>A<br>392<br>A<br>393	matches<br>matches<br>matches<br>matches	A<br>139<br>A<br>111<br>A<br>136<br>A<br>120	none<br>none<br>none<br>none	1.42 A	Submit
3ujg<br>PDB<br>PDBsum	SERINE/THREONINE-PROTEIN KINASE<br>SRK2E	D<br>E<br>D<br>D	A<br>326<br>A<br>389<br>A<br>392<br>A<br>393	matches<br>matches<br>matches<br>matches	B<br>492<br>B<br>203<br>B<br>204<br>B<br>243	-2.3 A from<br>MG MG B<br>512s<br>5.7 A from<br>MG MG B<br>100<br>-4.1 A from<br>MG MG B<br>100s<br>2.6 A from<br>MG MG B<br>512s	1.43 A	Submit
1w6t	ENOLASE	D	A	matches	A	-3.1 A from	1.43 A	Submit

PDB<br>PDBsum		E<br>D<br>D	326 matches 318<br>A matches A<br>389 matches 247<br>A A<br>392 292<br>A A<br>393 242	MG MG A 435s<br>8.5 A from MG MG A 435<br>5.0 A from MG MG A 435<br>-2.5 A from MG MG A 435s		
2akz<br>PDB<br>PDBsum	GAMMA ENOLASE	D<br>E<br>D<br>D	A matches A<br>326 matches 318<br>A matches A<br>389 matches 249<br>A A<br>392 293<br>A A<br>393 244	3.9 A from MG MG A 441<br>-3.3 A from O3 TRS A 460s<br>5.0 A from MG MG A 440<br>-2.5 A from MG MG A 440s	1.44 A	Submit
3tu8<br>PDB<br>PDBsum	BURKHOLDERIA LETHAL FACTOR 1 (BLF1)	D<br>E<br>D<br>D	A matches A<br>326 matches 51<br>A matches A<br>389 matches 22<br>A A<br>392 49<br>A A<br>393 24	24.1 A from OD CSO A 94<br>17.0 A from OD CSO A 94<br>19.6 A from OD CSO A 94<br>16.1 A from OD CSO A 94	1.44 A	Submit
3lhl<br>PDB<br>PDBsum	PUTATIVE AGMATINASE	D<br>E<br>D<br>D	A matches A<br>326 matches 138<br>A matches A<br>389 matches 261<br>A A<br>392 134<br>A A<br>393 217	2.8 A from MN MN A 301s<br>-4.3 A from MN MN A 303s<br>2.5 A from MN MN A 301s<br>-2.1 A from MN MN A 302s	1.44 A	Submit
2aeb<br>PDB<br>PDBsum	ARGINASE 1	D<br>E<br>D<br>D	A matches A<br>326 matches 128<br>A matches A<br>389 matches 277<br>A A<br>392 124<br>A A<br>393 234	2.0 A from HO1 ABH A 551s<br>-3.7 A from HO3 ABH A 551s<br>-2.6 A from MN MN A2363s<br>2.0 A from MN MN A2362s	1.45 A	Submit
4gl0<br>PDB<br>PDBsum	LMO0810 PROTEIN	D<br>E<br>D<br>D	A matches A<br>326 matches 44<br>A matches A<br>389 matches 220<br>A A<br>392 219<br>A A<br>393 68	5.6 A from O1 PGE A 501<br>9.9 A from O1 PGE A 501<br>-3.0 A from O1 PGE A 501s<br>-7.8 A from C1 PGE A 501s	1.45 A	Submit
2dew<br>PDB<br>PDBsum	PROTEIN-ARGININE DEIMINASE TYPE IV	D<br>E<br>D	A matches X<br>326 matches 388<br>A matches X<br>389 matches 175	-3.1 A from CA CA X 902s<br>8.5 A from	1.45 A	Submit

		D	A 392 A 393 X 157 X 176	CA CA X 902 2.3 A from CA CA X 902s -3.4 A from CA CA X 901s		
5iz5 PDB PDBsum	CYTOSOLIC PHOSPHOLIPASE A2 DELTA	D E D D	A 326 matches A 96<br>A 389 matches A 100<br>A 392 A 101<br>A 393 A 102	21.3 A from O3 SO4 A 903 14.2 A from O3 SO4 A 903 14.6 A from O3 SO4 A 903 18.4 A from O3 SO4 A 903	1.45 A	Submit
4yzg PDB PDBsum	PROTEIN PHOSPHATASE 2C 57	D E D D	A 326 matches A 339<br>A 389 matches A 73<br>A 392 A 74<br>A 393 A 93	-2.5 A from MN MN A 402s 5.3 A from MN MN A 401 4.8 A from MN MN A 402 2.6 A from MN MN A 402s	1.46 A	Submit
3qn1 PDB PDBsum	ABSCISIC ACID RECEPTOR PYR1	D E D D	A 326 matches B 492<br>A 389 matches B 203<br>A 392 B 204<br>A 393 B 243	-2.6 A from MN MN B 1s 5.3 A from MN MN B 2 4.8 A from MN MN B 1 2.5 A from MN MN B 1s	1.46 A	Submit
4oic PDB PDBsum	BET V I ALLERGEN-LIKE	D E D D	A 326 matches B 448<br>A 389 matches B 163<br>A 392 B 164<br>A 393 B 205	-2.8 A from MN MN B 501s 5.4 A from MN MN B 502 5.1 A from MN MN B 501 2.6 A from MN MN B 501s	1.46 A	Submit
1t5j PDB PDBsum	HYPOTHETICAL PROTEIN MJ1187	D E D D	A 326 matches A 19<br>A 389 matches A 64<br>A 392 A 61<br>A 393 A 97	5.6 A from MG A MG A 500 11.2 A from MG A MG A 500 -3.0 A from MG A MG A 500s 4.9 A from MG A MG A 500	1.47 A	Submit
4xlz PDB PDBsum	UNCHARACTERIZED PROTEIN	D E D D	A 326 matches A 69<br>A 389 matches A 113<br>A 392 A 111<br>A 393 A 150	12.2 A from CD CD A 301 13.8 A from C5 HEZ A 308 7.3 A from CD CD A 301 7.8 A from CD CD A	1.47 A	Submit

				301		
2cm5<br>PDB<br>PDBsum	RABPHILIN-3A	D<br>E<br>D<br>D	A 326 matches A 571<br>A 389 matches A 530<br>A 392 A 633<br>A 393 A 639	2.2 A from CA CA A1679s<br>8.0 A from CA CA A1678<br>2.2 A from CA CA A1678s<br>-3.3 A from CA CA A1678s	1.47 A	Submit
3nio<br>PDB<br>PDBsum	GUANIDINOBUTYRASE	D<br>E<br>D<br>D	A 326 matches A 156<br>A 389 matches A 287<br>A 392 A 152<br>A 393 A 245	-3.0 A from MN MN A1601s<br>5.8 A from MN MN A1601<br>2.7 A from MN MN A1602s<br>-2.2 A from MN MN A1602s	1.47 A	Submit
1h30<br>PDB<br>PDBsum	GROWTH-ARREST-SPECIFIC PROTEIN	D<br>E<br>D<br>D	A 326 matches A 329<br>A 389 matches A 331<br>A 392 A 656<br>A 393 A 654	-2.4 A from CA CA A 701s<br>-6.4 A from CA CA A 701s<br>-3.4 A from CA CA A 701s<br>4.8 A from CA CA A 701	1.47 A	Submit
4jnd<br>PDB<br>PDBsum	CA(2+)/CALMODULIN-DEPENDENT PROTEIN KINASE PHOSPHAT	D<br>E<br>D<br>D	A 326 matches A 415<br>A 389 matches A 176<br>A 392 A 177<br>A 393 A 202	-2.6 A from MG MG A 501s<br>5.1 A from MG MG A 502<br>4.8 A from MG MG A 501<br>2.5 A from MG MG A 501s	1.47 A	Submit
3pzl<br>PDB<br>PDBsum	AGMATINE UREOHYDROLASE	D<br>E<br>D<br>D	A 326 matches A 148<br>A 389 matches A 272<br>A 392 A 144<br>A 393 A 231	-2.3 A from MN MN A 314s<br>5.3 A from MN MN A 314<br>2.6 A from MN MN A 315s<br>-2.2 A from MN MN A 315s	1.47 A	Submit
4d25<br>PDB<br>PDBsum	BMVLG PROTEIN	D<br>E<br>D<br>D	A 326 matches A 538<br>A 389 matches A 412<br>A 392 A 536<br>A 393 A 540	31.9 A from O3G ANP A1564<br>29.3 A from O3G ANP A1564<br>27.7 A from O3G ANP A1564<br>22.2 A from O3G ANP A1564	1.48 A	Submit

1w6t PDB PDBsum	ENOLASE	D E D D	A 326 matches A 319<br>A 389 matches A 247<br>A 392 A 292<br>A 393 A 242	5.4 A from MG MG A 435<br>8.5 A from MG MG A 435<br>5.0 A from MG MG A 435<br>-2.5 A from MG MG A 435s	1.48 A	Submit
3pb6 PDB PDBsum	GLUTAMINYL-PEPTIDE CYCLOTRANSFERASE-LIKE PROTEIN	D E D D	A 326 matches X 269<br>A 389 matches X 325<br>A 392 X 326<br>A 393 X 327	-3.0 A from C1 CAC X 390s<br>7.4 A from O2 CAC X 390<br>4.2 A from C1 CAC X 390<br>6.5 A from C1 CAC X 390	1.48 A	Submit
4jd0 PDB PDBsum	NUCLEOTIDYL TRANSFERASE	D E D D	A 326 matches A 138<br>A 389 matches A 217<br>A 392 A 223<br>A 393 A 219	9.7 A from O4 A1KH A 301<br>-3.6 A from O4 A1KH A 301s<br>9.1 A from NA NA A 315<br>-3.5 A from NA NA A 315s	1.48 A	Submit
2pnq PDB PDBsum	[PYRUVATE DEHYDROGENASE [LIPOAMIDE]]-PHOSPHATASE	D E D D	A 326 matches A 445<br>A 389 matches A 53<br>A 392 A 54<br>A 393 A 73	-2.5 A from MG MG A 502s<br>4.9 A from MG MG A 501<br>4.6 A from MG MG A 502<br>2.6 A from MG MG A 502s	1.48 A	Submit
5msm PDB PDBsum	SISTER CHROMATID COHESION PROTEIN DCC1	D E D D	A 326 matches A 340<br>A 389 matches A 342<br>A 392 A 343<br>A 393 A 299	none<br>none<br>none<br>none	1.48 A	Submit
3b77 PDB PDBsum	UNCHARACTERIZED PROTEIN	D E D D	A 326 matches A 113<br>A 389 matches A 90<br>A 392 A 98<br>A 393 A 96	none<br>none<br>none<br>none	1.48 A	Submit
5lds PDB PDBsum	AMINOPEPTIDASE N	D E D D	A 326 matches A 551<br>A 389 matches A 633<br>A 392 A 632<br>A 393 A 634	25.4 A from C8 NAG A1014<br>27.1 A from C1 NAG A1003<br>22.5 A from C8 NAG A1014<br>23.7 A from O5 NAG	1.49 A	Submit

				A1003		
1lns<br>PDB<br>PDBsum	X-PROLYL DIPEPTIDYL AMINOPEPTIDASE	D<br>E<br>D<br>D	A 326 matches A 296<br>A 389 matches A 180<br>A 392 A 186<br>A 393 A 178	none<br>none<br>none<br>none	1.49 A	Submit
3i5g<br>PDB<br>PDBsum	MYOSIN HEAVY CHAIN ISOFORM A	D<br>E<br>D<br>D	A 326 matches B 28<br>A 389 matches B 36<br>A 392 B 37<br>A 393 B 40	none<br>none<br>none<br>none	1.50 A	Submit
4dz4<br>PDB<br>PDBsum	AGMATINASE	D<br>E<br>D<br>D	A 326 matches A 163<br>A 389 matches A 286<br>A 392 A 159<br>A 393 A 244	-2.5 A from MN MN A 402s<br>3.9 A from O UNK A 408<br>2.5 A from MN MN A 402s<br>-2.0 A from MN MN A 401s	1.50 A	Submit
6cgs<br>PDB<br>PDBsum	CADHERIN-7	D<br>E<br>D<br>D	A 326 matches A 187<br>A 389 matches A 103<br>A 392 A 132<br>A 393 A 134	-3.3 A from CA CA A 303s<br>9.0 A from CA CA A 303<br>-2.2 A from CA CA A 303s<br>3.3 A from CA CA A 302s	1.50 A	Submit
5hj9<br>PDB<br>PDBsum	ARGINASE	D<br>E<br>D<br>D	A 326 matches A 141<br>A 389 matches A 288<br>A 392 A 137<br>A 393 A 245	2.6 A from MN MN A 401s<br>-2.9 A from H15 X7A A 403s<br>-2.5 A from MN MN A 401s<br>2.1 A from MN MN A 402s	1.50 A	Submit
1woh<br>PDB<br>PDBsum	AGMATINASE	D<br>E<br>D<br>D	A 326 matches A 147<br>A 389 matches A 274<br>A 392 A 143<br>A 393 A 231	0.6 A from CG ASP A 147<br>0.6 A from CD GLU A 274<br>0.5 A from CG ASP A 143<br>0.6 A from CG ASP A 231	1.51 A	Submit
5g4d<br>PDB<br>PDBsum	CRISPR-ASSOCIATED ENDORIBONUCLEASE CAS2	D<br>E<br>D<br>D	A 326 matches A 56<br>A 389 matches A 55<br>A 392 A 54<br>A 393 A 58	none<br>none<br>none<br>none	1.51 A	Submit

2dew<br>PDB<br>PDBsum	PROTEIN-ARGININE DEIMINASE TYPE IV	D<br>E<br>D<br>D	A matches X<br>326 matches 155<br>A matches X<br>389 matches 252<br>A X<br>392 179<br>A X<br>393 176	2.8 A from CA CA X 901s<br>4.4 A from CA CA X 904<br>2.3 A from CA CA X 902s<br>-3.4 A from CA CA X 901s	1.51 A	Submit
1gq6<br>PDB<br>PDBsum	PROCLAVAMINATE AMIDINO HYDROLASE	D<br>E<br>D<br>D	A matches A<br>326 matches 148<br>A matches A<br>389 matches 279<br>A A<br>392 144<br>A A<br>393 237	-2.6 A from MN MN A 350s<br>5.4 A from MN MN A 350<br>2.6 A from MN MN A 350s<br>-1.9 A from MN MN A 351s	1.51 A	Submit
5osw<br>PDB<br>PDBsum	ALBUMIN	D<br>E<br>D<br>D	A matches A<br>326 matches 255<br>A matches A 6<br>389 matches A<br>A 254<br>392 A<br>A 13<br>393	11.1 A from I2 DIU A 601<br>18.7 A from I2 DIU A 601<br>12.7 A from I2 DIU A 601<br>15.5 A from I2 DIU A 601	1.51 A	Submit
5uc2<br>PDB<br>PDBsum	DOMAIN OF UNKNOWN FUNCTION DUF1849	D<br>E<br>D<br>D	A matches A<br>326 matches 103<br>A matches A<br>389 matches 100<br>A A<br>392 101<br>A A<br>393 106	20.5 A from O2 GOL A 301<br>11.3 A from O2 GOL A 301<br>19.5 A from O1 EDO A 302<br>17.7 A from O1 EDO A 302	1.51 A	Submit
4xgq<br>PDB<br>PDBsum	RIBONUCLEASE VAPC30	D<br>E<br>D<br>D	A matches A<br>326 matches 119<br>A matches A<br>389 matches 40<br>A A<br>392 99<br>A A 4<br>393	7.1 A from MG MG A 201<br>-4.0 A from MG MG A 201s<br>-3.9 A from MG MG A 201s<br>-2.8 A from MG MG A 201s	1.52 A	Submit
5z9x<br>PDB<br>PDBsum	SMALL RNA DEGRADING NUCLEASE 1	D<br>E<br>D<br>D	A matches A<br>326 matches 228<br>A matches A<br>389 matches 146<br>A A<br>392 144<br>A A<br>393 283	-3.5 A from MG MG A 502s<br>-3.1 A from MG MG A 503s<br>2.8 A from MG MG A 502s<br>-3.0 A from MG MG A 503s	1.52 A	Submit
2d5r<br>PDB<br>PDBsum	CCR4-NOT TRANSCRIPTION COMPLEX SUBUNIT 7	D<br>E<br>D<br>D	A matches A<br>326 matches 161<br>A matches A<br>389 matches 42<br>A A<br>392 40<br>A A	none<br>none<br>none<br>none	1.52 A	Submit

			393 230			
1lw7<br>PDB<br>PDBsum	TRANSCRIPTIONAL REGULATOR NADR	D<br>E<br>D<br>D	A 326 matches A 140<br>A 389 matches A 139<br>A 392 matches A 95<br>A 393 matches A 99	9.9 A from O3D NAD A 601<br>-2.5 A from O3D NAD A 601s<br>7.6 A from O2D NAD A 601<br>7.2 A from O3D NAD A 601	1.52 A	Submit
1g71<br>PDB<br>PDBsum	DNA PRIMASE	D<br>E<br>D<br>D	A 326 matches A 55<br>A 389 matches A 60<br>A 392 matches A 58<br>A 393 matches A 61	26.4 A from CL CL A 349<br>23.3 A from O3 SO4 A 352<br>25.2 A from CL CL A 349<br>25.5 A from O3 SO4 A 352	1.53 A	Submit
3vyw<br>PDB<br>PDBsum	MNMC2	D<br>E<br>D<br>D	A 326 matches A 289<br>A 389 matches A 286<br>A 392 matches A 285<br>A 393 matches A 207	13.1 A from N6 SAM A 501<br>18.1 A from N6 SAM A 501<br>9.5 A from N6 SAM A 501<br>14.1 A from N6 SAM A 501	1.53 A	Submit
3u2g<br>PDB<br>PDBsum	S-LAYER PROTEIN MA0829	D<br>E<br>D<br>D	A 326 matches A 364<br>A 389 matches A 454<br>A 392 matches A 453<br>A 393 matches A 452	23.3 A from C3 GOL A 796<br>24.0 A from O2 GOL A 796<br>19.0 A from O1 GOL A 796<br>23.3 A from O1 GOL A 796	1.54 A	Submit
2gre<br>PDB<br>PDBsum	DEBLOCKING AMINOPEPTIDASE	D<br>E<br>D<br>D	A 326 matches A 241<br>A 389 matches A 220<br>A 392 matches A 187<br>A 393 matches A 186	24.5 A from O3 SO4 A 350<br>27.4 A from O3 SO4 A 350<br>23.9 A from O3 SO4 A 350<br>29.8 A from O3 SO4 A 350	1.54 A	Submit
5dkx<br>PDB<br>PDBsum	ALPHA GLUCOSIDASE-LIKE PROTEIN	D<br>E<br>D<br>D	A 326 matches A 867<br>A 389 matches A 405<br>A 392 matches A 865<br>A 393 matches A 864	25.8 A from C1 TRS A1001<br>23.2 A from CL CL A1003<br>27.3 A from CL CL A1003<br>21.5 A from CL CL A1003	1.54 A	Submit
1pe9<br>PDB	PECTATE LYASE A	D<br>E	A 326 matches A 188	none<br>none	1.54 A	Submit

PDBsum		D D	A 389 A 392 A 393	matches matches	A 142 A 144 A 184	none none		
2zyr PDB PDBsum	LIPASE, PUTATIVE	D E D D	A 326 A 389 A 392 A 393	matches matches matches matches	A 431 A 345 A 405 A 409	-2.4 A from MG MG A2003s 8.0 A from MG MG A2003 -3.3 A from MG MG A2003s -2.9 A from MG MG A2003s	1.54 A	Submit
4a01 PDB PDBsum	PROTON PYROPHOSPHATASE	D E D D	A 326 A 389 A 392 A 393	matches matches matches matches	A 257 A 698 A 723 A 691	-2.5 A from MG MG A1767s 6.8 A from O4 2PN A1773 3.9 A from MG MG A1769 2.7 A from MG MG A1771s	1.54 A	Submit
5hhj PDB PDBsum	RETRON-TYPE REVERSE TRANSCRIPTASE	D E D D	A 326 A 389 A 392 A 393	matches matches matches matches	A 153 A 198 A 239 A 238	6.2 A from K K A 402 9.3 A from K K A 402 -3.6 A from K K A 402s 8.4 A from K K A 402	1.54 A	Submit
4irz PDB PDBsum	INTEGRIN ALPHA4 SUBUNIT	D E D D	A 326 A 389 A 392 A 393	matches matches matches matches	A 348 A 351 A 344 A 352	-2.7 A from CA CA A2006s 8.0 A from CA CA A2005 -3.2 A from CA CA A2006s -2.0 A from CA CA A2006s	1.55 A	Submit
2dew PDB PDBsum	PROTEIN-ARGININE DEIMINASE TYPE IV	D E D D	A 326 A 389 A 392 A 393	matches matches matches matches	X 165 X 175 X 179 X 388	3.3 A from CA CA X 901s 8.5 A from CA CA X 902 2.3 A from CA CA X 902s -3.1 A from CA CA X 902s	1.55 A	Submit
5wtl PDB PDBsum	OMPA FAMILY PROTEIN	D E D D	A 326 A 389 A 392 A 393	matches matches matches matches	A 254 A 267 A 263 A 260	2.6 A from CA CA A1005s 9.6 A from CA CA A1006 2.1 A from CA CA A1006s 5.4 A from CA CA A1005	1.56 A	Submit

1xfk<br>PDB<br>PDBsum	FORMIMIDOYLGLUTAMASE	D<br>E<br>D<br>D	A 326 matches A 161<br>A 389 matches A 300<br>A 392 matches A 157<br>A 393 matches A 256	none<br>none<br>none<br>none	1.56 A	Submit
3cih<br>PDB<br>PDBsum	PUTATIVE ALPHA-RHAMNOSIDASE	D<br>E<br>D<br>D	A 326 matches A 344<br>A 389 matches A 597<br>A 392 matches A 332<br>A 393 matches A 337	-2.6 A from N TRS A 800s<br>-3.7 A from O1 TRS A 800s<br>-3.6 A from O3 TRS A 800s<br>4.3 A from O3 TRS A 800	1.56 A	Submit
3niq<br>PDB<br>PDBsum	3-GUANIDINOPROPIONASE	D<br>E<br>D<br>D	A 326 matches A 152<br>A 389 matches A 284<br>A 392 matches A 148<br>A 393 matches A 242	-2.6 A from MN MN A1601s<br>5.3 A from MN MN A1601<br>2.6 A from MN MN A1602s<br>-2.1 A from MN MN A1602s	1.56 A	Submit
2gv9<br>PDB<br>PDBsum	DNA POLYMERASE	D<br>E<br>D<br>D	A 326 matches A 471<br>A 389 matches A 370<br>A 392 matches A 368<br>A 393 matches A 581	none<br>none<br>none<br>none	1.56 A	Submit
4n0g<br>PDB<br>PDBsum	PROTEIN PHOSPHATASE 2C 37	D<br>E<br>D<br>D	A 326 matches A 380<br>A 389 matches A 118<br>A 392 matches A 119<br>A 393 matches A 142	-2.8 A from MG MG A 403s<br>5.3 A from MG MG A 404<br>5.2 A from MG MG A 403<br>2.3 A from MG MG A 403s	1.56 A	Submit
3l6u<br>PDB<br>PDBsum	ABC-TYPE SUGAR TRANSPORT SYSTEM PERIPLASMIC COMPONE	D<br>E<br>D<br>D	A 326 matches A 228<br>A 389 matches A 21<br>A 392 matches A 204<br>A 393 matches A 205	9.0 A from O2 SO4 A 294<br>8.3 A from O4 SO4 A 294<br>11.0 A from O2 SO4 A 294<br>13.1 A from O4 SO4 A 294	1.57 A	Submit
4oy2<br>PDB<br>PDBsum	TRANSCRIPTION INITIATION FACTOR TFIID SUBUNIT 1	D<br>E<br>D<br>D	A 326 matches B 282<br>A 389 matches B 213<br>A 392 matches B 260<br>A 393 matches B 214	31.9 A from ZN ZN A1001<br>38.9 A from ZN ZN A1001<br>31.8 A from ZN ZN A1001<br>35.6 A from ZN ZN	1.58 A	Submit

				A1001		
5wzf<br>PDB<br>PDBsum	23S RRNA-SPECIFIC ENDONUCLEASE VAPC20	D<br>E<br>D<br>D	A 326 matches A 119<br>A 389 matches A 43<br>A 392 matches A 98<br>A 393 matches A 5	none<br>none<br>none<br>none	1.58 A	Submit
5flk<br>PDB<br>PDBsum	DHAA101	D<br>E<br>D<br>D	A 326 matches A 78<br>A 389 matches A 208<br>A 392 matches A 83<br>A 393 matches A 82	10.1 A from C2 PEG A1299<br>10.1 A from O1SBMES A1297<br>10.2 A from C2 PEG A1299<br>14.5 A from O3SBMES A1297	1.58 A	Submit
4a7k<br>PDB<br>PDBsum	ALDOS-2-ULOSE DEHYDRATASE	D<br>E<br>D<br>D	A 326 matches A 345<br>A 389 matches A 349<br>A 392 matches A 343<br>A 393 matches A 350	-2.6 A from ZN ZN A 951s<br>-6.4 A from ZN ZN A 951s<br>-3.0 A from ZN ZN A 951s<br>6.8 A from ZN ZN A 951	1.58 A	Submit
5wrt<br>PDB<br>PDBsum	SOLUBLE INORGANIC PYROPHOSPHATASE	D<br>E<br>D<br>D	A 326 matches A 192<br>A 389 matches A 174<br>A 392 matches A 190<br>A 393 matches A 227	5.4 A from MG MG A 402<br>6.7 A from MG MG A 401<br>-3.0 A from MG MG A 401s<br>-2.8 A from MG MG A 401s	1.58 A	Submit
2oyh<br>PDB<br>PDBsum	FIBRINOGEN ALPHA CHAIN	D<br>E<br>D	A 326 matches A 174<br>A 389 matches A 179<br>A 392 matches A 177	30.0 A from O2 FUC B 5<br>21.7 A from O3 FUC B 5<br>30.4 A from O2 FUC B 5	1.59 A	Submit

Back
