# Supplementary material for: Torpedo californica acetylcholinesterase is stabilized by binding of a divalent metal ion to a novel and versatile 4D motif
Source: Protein Sci. 2021 Mar 29;30(5):966–81. doi: 10.1002/pro.4061 (PMC8040873; doi:10.1002/pro.4061)
Supplement: Supplementary file 3 — Table S3 List of all AChE and BChE sequences that contain the 4D motif. [file PRO-30-966-s001.pdf]

31 Hits with 4D - out from 531 AChE Sequences
Based on sequences from the Esther Server
http://bioweb.supagro.inra.fr/ESTHER/general?what=overallTable
Lenfant N, Hotelier T, Velluet E, Bourne Y, Marchot P, Chatonnet A (2013) ESTHER, the database of the alpha/beta-hydrolase fold superfamily of proteins: tools to explore diversity of functions. Nucleic Acids Res 41:D423-429.

9chon-ACHE	1674	1917	1920	1921	D	D	D	D
torca-ACHE	1674	1917	1920	1921	D	D	D	D
torma-ACHE	1674	1917	1920	1921	D	D	D	D
calmi-ACHE	1674	1917	1920	1921	D	D	D	D
9tele-a0a0p7y2n7	1674	1917	1920	1921	D	D	D	D
cypca-ACHE	1674	1917	1920	1921	D	D	D	D
danre-ACHE	1674	1917	1920	1921	D	D	D	D
oncmy-a0a060wae0	1674	1917	1920	1921	D	D	D	D
salsa-a0a1s3seb0	1674	1917	1920	1921	D	D	D	D
salsa-a0a1s3n9k7	1674	1917	1920	1921	D	D	D	D
9tele-a0a0s7jbw8	1674	1917	1920	1921	D	D	D	D
xipma-m4abu7	1674	1917	1920	1921	D	D	D	D
poefo-a0a087y1w2	1674	1917	1920	1921	D	D	D	D
funhe-a0a146y019	1674	1917	1920	1921	D	D	D	D
9tele-a0a1a7ydq0	1674	1917	1920	1921	D	D	D	D
notfu-a0a1a7zwa9	1674	1917	1920	1921	D	D	D	D
9tele-a0a2i4cb12	1674	1917	1920	1921	D	D	D	D
fugru-ACHE	1674	1917	1920	1921	D	D	D	D
tetng-ACHE	1674	1917	1920	1921	D	D	D	D
oryla-ACHE	1674	1917	1920	1921	D	D	D	D
lepoc-w5n9n8	1674	1917	1920	1921	D	D	D	D
brafl-ACHEB	1674	1917	1920	1921	D	D	D	D
brafl-ACHEA	1674	1917	1920	1921	D	D	D	D
brafl-ACHE2	1674	1917	1920	1921	D	D	D	D
linun-a0a1s3kf66	1674	1917	1920	1921	D	D	D	D
lotgi-ACHE2	1674	1917	1920	1921	D	D	D	D
linun-a0a1s3hd03	1674	1917	1920	1921	D	D	D	D
capte-ACHE3	1674	1917	1920	1921	D	D	D	D
9plat-a0a1i8gq29	1674	1917	1920	1921	D	D	D	D
9plat-a0a1i8hd50.1	1674	1917	1920	1921	D	D	D	D
9plat-a0a267e1q9	1674	1917	1920	1921	D	D	D	D

nhits = 31

28 Hits with 4D - out from 90 BChE Sequences
Based on sequences from the Esther Server
http://bioweb.supagro.inra.fr/ESTHER/general?what=overallTable
Lenfant N, Hotelier T, Velluet E, Bourne Y, Marchot P, Chatonnet A (2013) ESTHER, the database of the alpha/beta-hydrolase fold superfamily of proteins: tools to explore diversity of functions. Nucleic Acids Res 41:D423-429.

ailme-BCHE	449	512	515	516	D	D	D	D
canfa-BCHE	449	512	515	516	D	D	D	D
pig-BCHE	449	512	515	516	D	D	D	D
otoga-h0wqs2	449	512	515	516	D	D	D	D
crigr-a0a061ikl2	449	512	515	516	D	D	D	D
mesau-a0a1u7ql18	449	512	515	516	D	D	D	D
mouse-BCHE	449	512	515	516	D	D	D	D
allmi-a0a151nb42	449	512	515	516	D	D	D	D
pelsi-BCHE	449	512	515	516	D	D	D	D
trasc-BCHE	449	512	515	516	D	D	D	D
amaae-a0a0q3p4p0	449	512	515	516	D	D	D	D
anapl-BCHE	449	512	515	516	D	D	D	D
limla-a0a2i0u4q4	449	512	515	516	D	D	D	D
colli-r7vnu6	449	512	515	516	D	D	D	D
chick-BCHE	449	512	515	516	D	D	D	D
melga-g1ndd8	449	512	515	516	D	D	D	D
fical-u3k1z7	449	512	515	516	D	D	D	D
taegu-BCHE	449	512	515	516	D	D	D	D
eubma-a0a098lwp6	449	512	515	516	D	D	D	D
mondo-BCHE	449	512	515	516	D	D	D	D
sarha-g3wuz4	449	512	515	516	D	D	D	D
croad-a0a0f7zed7	449	512	515	516	D	D	D	D
echco-a0a0a1wch5	449	512	515	516	D	D	D	D
xenla-a0a1l8f4t7	449	512	515	516	D	D	D	D
xenla-a0a1l8fct2	449	512	515	516	D	D	D	D
xentr-BCHE1	449	512	515	516	D	D	D	D
xenla-BCHE2	449	512	515	516	D	D	D	D
xentr-BCHE2	449	512	515	516	D	D	D	D

nhits = 28
